# Chemical and Enzymatic Synthesis of Sialylated Glycoforms of Human Erythropoietin

**DOI:** 10.1002/anie.202110013

**Published:** 2021-11-02

**Authors:** Hendrik Hessefort, Angelina Gross, Simone Seeleithner, Markus Hessefort, Tanja Kirsch, Lukas Perkams, Klaus Ole Bundgaard, Karen Gottwald, David Rau, Christopher Günther Franz Graf, Elisabeth Rozanski, Sascha Weidler, Carlo Unverzagt

**Affiliations:** ^1^ University of Bayreuth Bioorganic Chemistry Universitätsstraße 30 95447 Bayreuth Germany

**Keywords:** glycopeptides, glycoproteins, native chemical ligation, oligosaccharide, receptor

## Abstract

Recombinant human erythropoietin (EPO) is the main therapeutic glycoprotein for the treatment of anemia in cancer and kidney patients. The in‐vivo activity of EPO is carbohydrate‐dependent with the number of sialic acid residues regulating its circulatory half‐life. EPO carries three *N*‐glycans and thus obtaining pure glycoforms provides a major challenge. We have developed a robust and reproducible chemoenzymatic approach to glycoforms of EPO with and without sialic acids. EPO was assembled by sequential native chemical ligation of two peptide and three glycopeptide segments. The glycopeptides were obtained by pseudoproline‐assisted Lansbury aspartylation. Enzymatic introduction of the sialic acids was readily accomplished at the level of the glycopeptide segments but even more efficiently on the refolded glycoprotein. Biological recognition of the synthetic EPOs was shown by formation of 1:1 complexes with recombinant EPO receptor.

## Graphical abstract

Two glycoforms of human erythropoietin (EPO) were synthesized by sequential native chemical ligation combining chemical and enzymatic methods. The sialylated glycoform **EPO S** was assembled stepwise from sialoglycopeptides but more rapidly by direct sialylation of the asialo glycoform **EPO A**. The native fold was evident from CD spectra, HR‐MS, and binding to the human EPO receptor (**EPOR**).

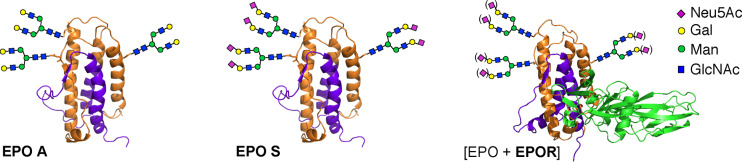

## Introduction

The hematopoietic cytokine erythropoietin (EPO) is a glycoprotein hormone with a central role in erythrocyte homeostasis. Under hypoxic conditions the expression of EPO in renal and hepatic cells is upregulated. Secreted EPO binds to the EPO receptor on progenitor cells in the bone marrow and stimulates their differentiation into erythrocytes.^[1]^ Seminal studies of the 166 amino acid glycoprotein EPO revealed that its in vivo bioactivity strongly depends on the presence of multiantennary sialylated *N*‐glycans on Asn residues 24, 38 and 83.^[2]^ The typical *N*‐glycans on EPO are tetraantennary and core fucosylated.^[3]^ The *O*‐glycan on Ser‐126 is not essential for activity but contributes to serum half‐life.^[4]^ Enzymatic desialylation of EPO resulted in increased binding to the EPO receptor, however, the in vivo activity was highly reduced.^[5]^

Due to the heterogeneity of the three *N*‐glycans the separation of recombinant EPO into pure glycoforms is not feasible even with the most advanced methods.^[6]^ Thus, synthetic approaches for analogs of EPO^[7]^ and homogeneously glycosylated EPO were established.^[8]^ The use of sequential native chemical ligation^[9]^ (NCL) has provided pure glycoforms^[10]^ of EPO with excellent control of site specific glycosylation. Sialylated *N*‐glycans can be installed via glycosyl‐asparagine derivatives bearing protected sialic acids,^[8c]^ by Lansbury aspartylation^[8a]^ or by enzymatic modification.^[11]^ Here, we describe the chemical and enzymatic synthesis of glycoforms of EPO with terminal galactose or sialic acid using a set of five building blocks. Based on a generally applicable protecting group strategy the syntheses are robust and reproducible. A straight‐forward alternative to the multi‐step enzymatic synthesis and ligation of sialylated glycopeptides was the enzymatic sialylation of refolded EPO in a single step.

**Scheme 1:**
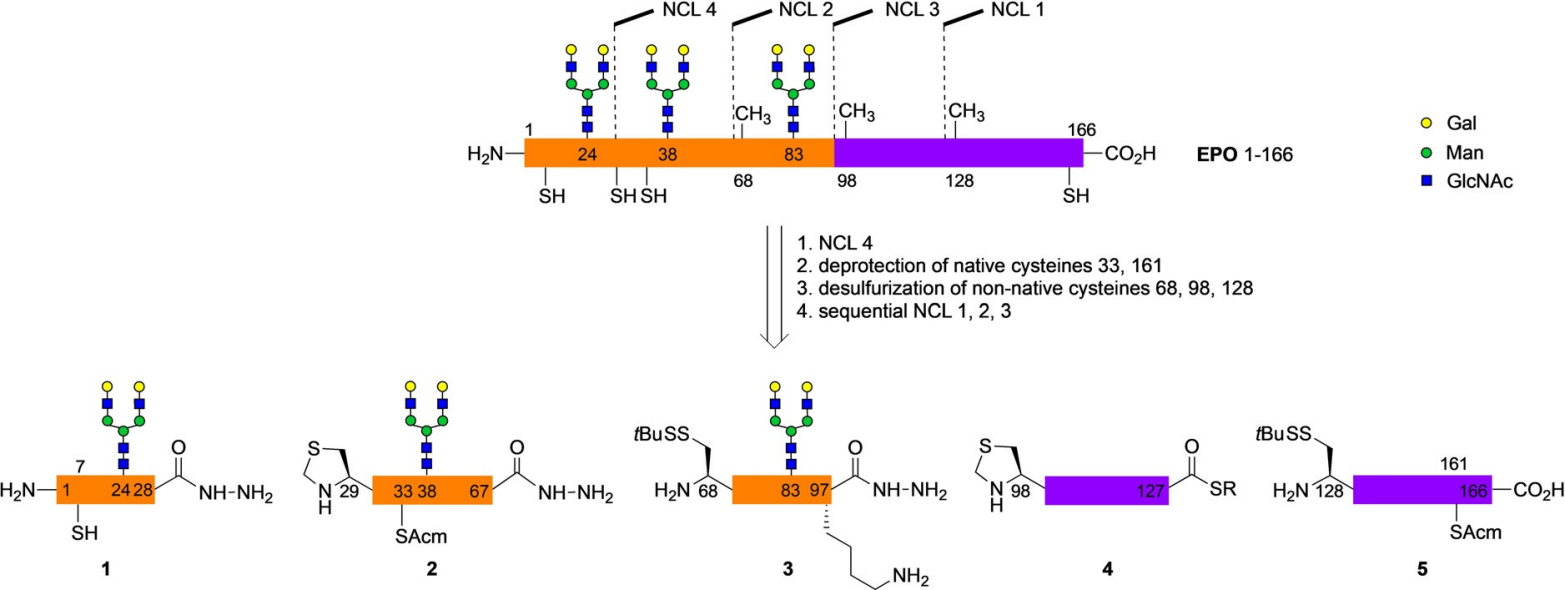
Retrosynthesis of human EPO 1–166 with three asialo *N*‐glycans leading to fragments **1**–**5**.

We intended to establish an approach to sialylated EPO by NCL using enzymatically sialylated glycopeptide segments.^[12]^ The introduction of the sialic acids at a late stage of the synthesis was expected to facilitate access to the biologically relevant sialylated glycoforms. A prerequisite for the enzymatic sialylations of EPO glycopeptides was a stable C‐terminal functionalization resistant to hydrolysis. Thus, the use of glycopeptide thioesters^[8a]^ was not considered and highly stable hydrazides^[13]^ suitable for sequential NCL were chosen instead. Due to the lack of appropriately placed cysteines in EPO artificial ligation sites at alanine 68, 98 and 128^[8a]^ were introduced leading to five segments with a length of about forty amino acids (Scheme 1). This approach requires a desulfurization step^[14]^ converting the three non‐natural cysteines to native alanines after the ligations. A key to this endeavour was to develop a reliable protecting group strategy for the native cysteines compatible with the synthesis of the sialylated glycopeptides, the sequential ligations and the desulfurization step.

## Results and Discussion

Our goal was to first synthesize an **EPO** 1–166 glycoprotein bearing three biantennary *N*‐glycans terminating with galactose via the fragments **1**–**5** and to subsequently extend the approach towards sialylated EPO glycoforms. This initial strategy was based on Acm protection of the native cysteines 33, 161, and temporary protection of the *N*‐terminal cysteines in fragments **2** and **4** by thiazolidines (Scheme 1).^[8]^ The synthesis of the three glycopeptide hydrazides **1**–**3** was envisioned by convergent pseudoproline‐assisted Lansbury aspartylation^[15]^ in solution using allyl esters at the glycosylation sites.^[16]^ An exploratory attempt to assemble the demanding EPO 29–97 sequence on the solid phase by fragment condensation^[17]^ and subsequently attach *N*‐glycans to Asp 38 and 83 was feasible for GlcNAcNH_2_ but not for larger *N*‐glycan amines (data not shown). Thus, the idea of a four‐segment approach to EPO was abandoned and the synthesis was continued with the more readily accessible glycopeptide segments **2** and **3**.

**Scheme 2:**
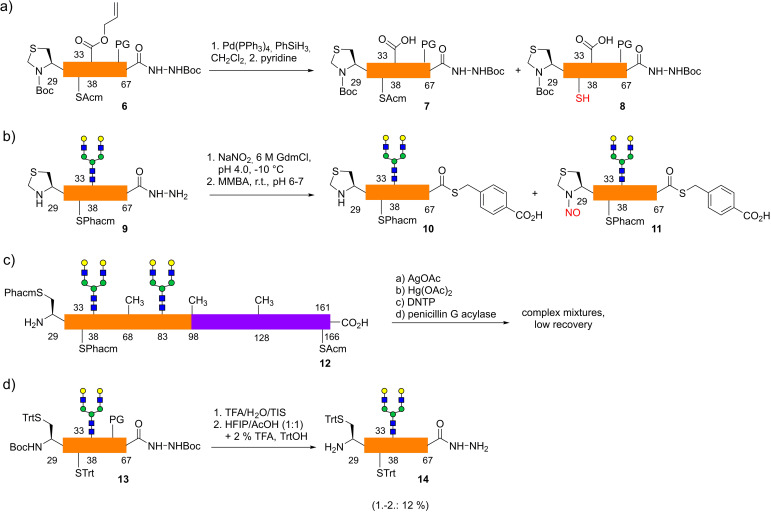
Drawbacks of the initial protecting group strategies for native cysteines: a) loss of Acm during Pd^0^‐catalyzed deallylation; b) *N*‐terminal Thz forming a stable nitroso derivative under diazotization conditions; c) difficult removal of Phacm at a late stage using various deprotection methods; d) low overall yield for *S*‐tritylation and low solubility in following transformations.

The EPO 29–67 peptide was obtained by Fmoc‐SPPS on a trityl‐ChemMatrix‐resin (Trt‐CM) using pseudoproline dipeptides^[18]^ and DMB‐glycine to reduce aggregation during synthesis. To avoid sulfoxidation Met‐54 was replaced by norleucine.^[19]^ The peptide was cleaved from the resin and converted in situ^[20]^ to hydrazide **6** (Scheme 2 a). In the course of Pd^0^‐catalyzed deallylation^[21]^ of **6** a considerable loss of the Acm group was observed (**7**:**8**=3:1). Thus, a Phacm group^[22]^ was installed at Cys‐33, which remained stable during deallylation and provided the desired 29–67 glycopeptide hydrazide **9** (Scheme 2 b). However, in the following conversion of hydrazide **9** to thioester **10** the *N*‐terminal thiazolidine gave rise to the stable side product **11** with a higher mass (M+29,^[23]^ presumably *N*‐nitroso) and resistance to ring opening with methoxyamine[^13^, ^23^, ^24^] (data not shown). Hence, Cys‐29 and Cys‐33 in EPO 29–67 were protected with a Phacm group and after three sequential ligations using fragments **3**, **4** and **5** the desulfurized EPO 29–166 glycopeptide **12** could be obtained. At this stage the removal of the Phacm and Acm groups using Ag^I^‐ or Hg^II^‐salts,^[25]^ 2,2′‐dithiobis(5‐nitropyridine)^[26]^ or penicillin G acylase^[22]^ was very troublesome and suffered from incomplete conversion and low recovery (data not shown).

To overcome the difficulties associated with Acm‐type protecting groups in the 29–67 glycopeptide *S*‐trityl protecting groups were selectively reinstalled at both cysteines^[27]^ after global deprotection of the glycopeptide **13**. However, the two proximal trityl groups of segment **14** led to low solubility and difficult purifications even after the following ligation (data not shown).

**Scheme 3:**
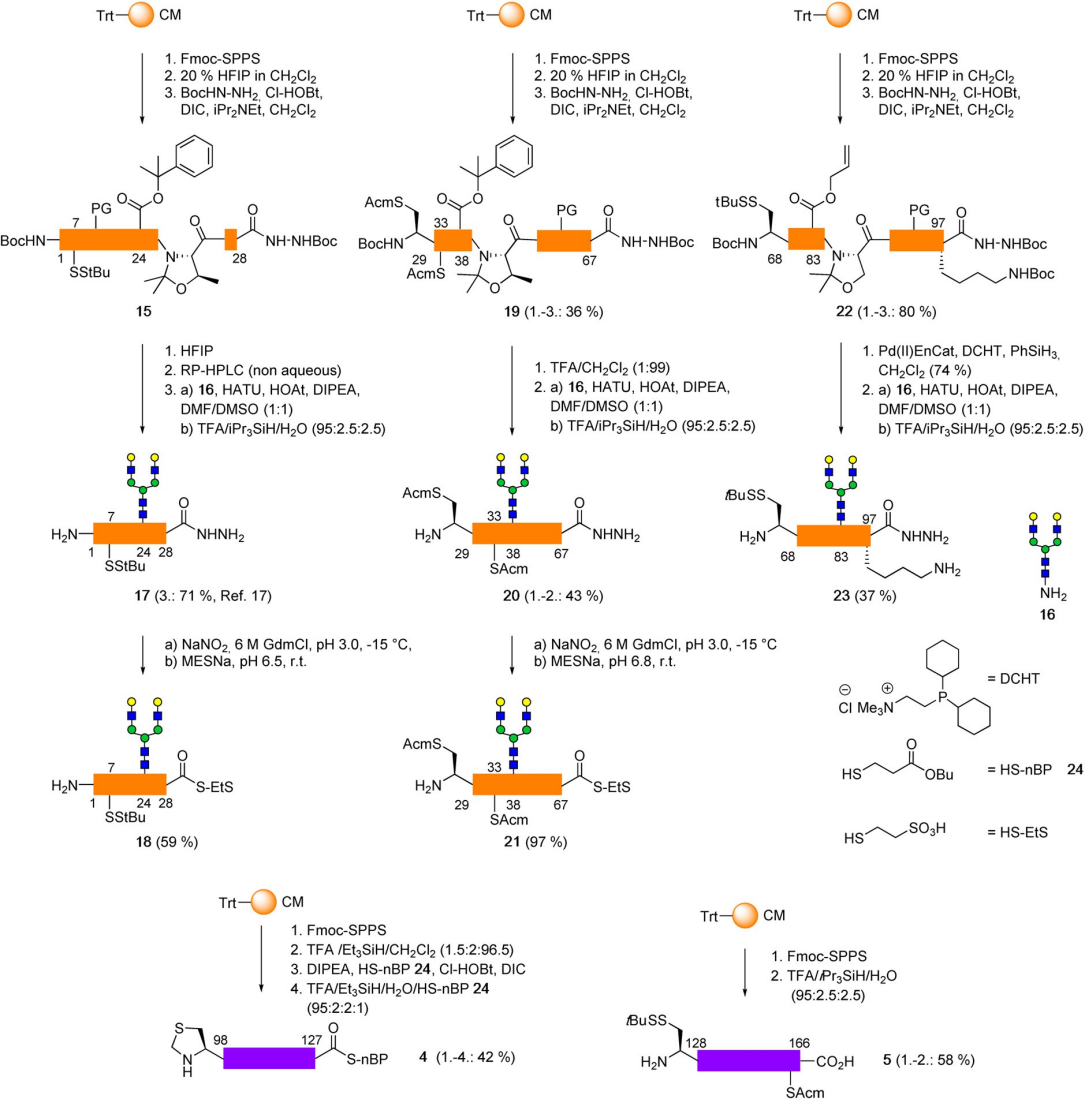
Solid phase synthesis and functionalization of the five EPO segments. The glycopeptide hydrazides **17**, **20** and **23** were obtained using pseudoproline‐assisted Lansbury aspartylation.

Since all the initially tested protecting group combinations exhibited serious drawbacks, we finally returned to using the Acm protecting group in the EPO glycopeptide 29–67 but replaced the allylester in Asp‐38 by an acid‐labile phenylisopropyl ester (PhiPr),^[28]^ thus avoiding the use of Pd^0^ in the presence of Acm groups. The EPO 29–67 peptide was synthesized on Trt‐CM resin, cleaved with 20 % HFIP in CH_2_Cl_2_ and equipped with a C‐terminal hydrazide (Scheme 3). After purification by flash chromatography the PhiPr ester was removed with 1 % TFA/CH_2_Cl_2_. The deprotection was monitored by RP‐HPLC to minimize cleavage of other acid‐sensitive protecting groups. After pseudoproline‐assisted aspartylation of the resulting EPO 29–67 aspartyl peptide (**S4**) with *N*‐glycan **16** ^[15b]^ the desired glycopeptide hydrazide **20** was obtained in 43 % yield.

Glycopeptide hydrazide **17** (EPO 1–28) was synthesized very efficiently^[29]^ from the 1–28 peptide hydrazide **15** bearing a phenylisopropyl ester^[28]^ at the glycosylation site (Scheme 3). The key to the high yield was the amenability of the selectively deprotected aspartyl peptide to purification by non‐aqueous HPLC.^[29]^

The synthesis of glycopeptide hydrazide EPO 68–97 was also examined using a PhiPr ester at Asp 83. However, cleavage of the PhiPr moiety of the 68–97 hydrazide using 1 % TFA in CH_2_Cl_2_ also opened one of the two pseudoprolines (71 or 85) according to RP‐HPLC‐MS. The extensive aspartimide formation during the subsequent aspartylation with **16** suggested that the acetonide at Ser 85 was mainly affected. Since less acidic conditions did not provide fully orthogonal cleavage of the PhiPr ester in this segment, the EPO 68–97 hydrazide **22** was assembled with an allylester at Asp 83. A modified deallylation of **22** was carried out using immobilized Pd^II^ acetate^[30]^ in combination with the water‐soluble phosphorus ligand DCHT, which improved the workup and the purity of the aspartyl peptide **S8**. Subsequent attachment of the glycan **16** provided the glycopeptide hydrazide **23** in 37 % yield. The glycopeptide hydrazides **17** and **20** were readily converted^[13]^ to the corresponding thioesters **18** and **21** by first transforming the hydrazide to an acylazide (NaNO_2_, pH 3, −15 °C) followed by thiolysis with MESNa at near‐neutral pH. The C‐terminal segments EPO 98–127 thioester **4** (42 % yield) and the Cys‐peptide EPO 128–166 **5** (58 % yield) were obtained by Fmoc‐SPPS.

**Scheme 4:**
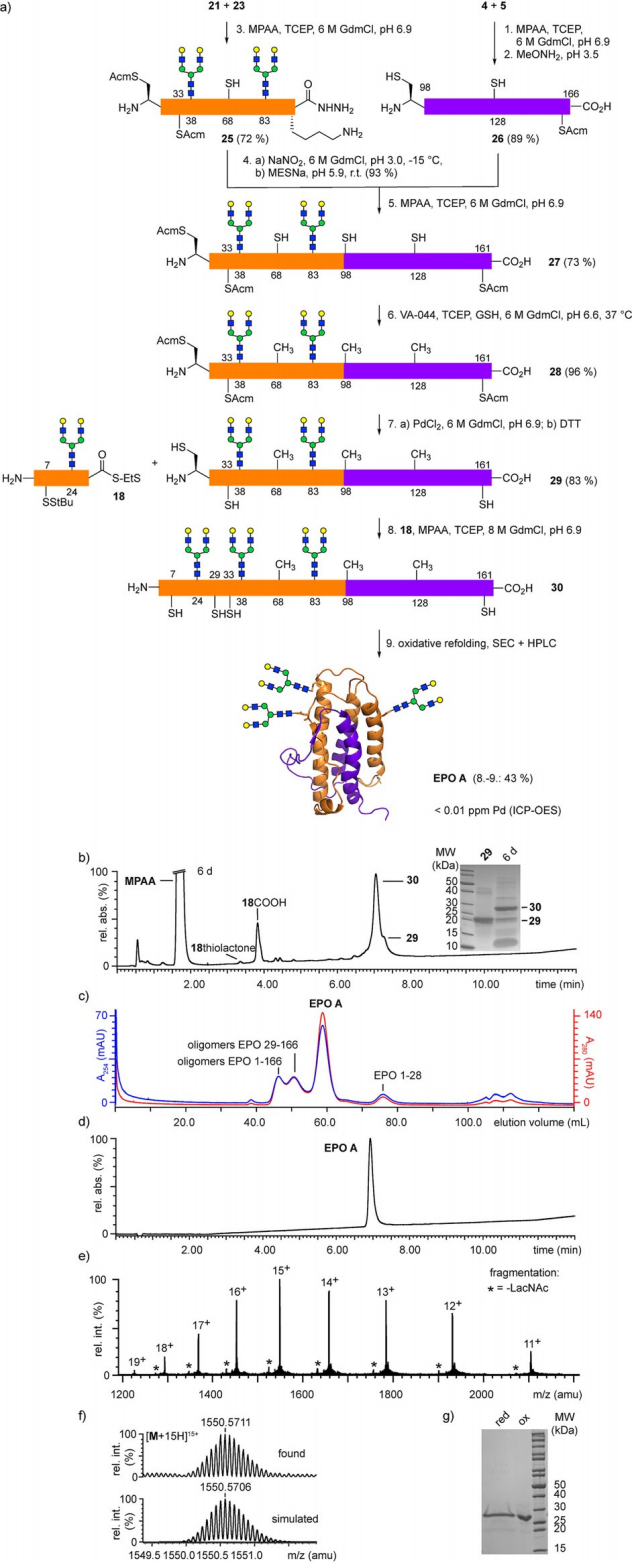
a) Synthesis of the asialoglycoform **EPO A** bearing three *N*‐glycans by sequential native chemical ligation, desulfurization, Pd‐mediated cleavage of the Acm groups and oxidative refolding; b) RP‐HPLC‐MS of the final ligation of glycopeptide thioester **18** and glycopeptide **29** to full‐length EPO 1–166 **30** after 6 d, insert shows SDS‐PAGE of crude ligation mixture; c) SEC after oxidative refolding of ligation mixture; d) RP‐HPLC‐MS of **EPO A** after RP‐HPLC purification; e) HR‐MS of purified **EPO A** (H_2_O, direct injection); f) simulated and measured isotope pattern of the [**M**+15 H]^15+^ HR‐MS peak; g) SDS‐PAGE (reduced and oxidized) of **EPO A**.

With all five EPO segments in hand the ligations and the following transformations were investigated (Scheme 4). The NCL of thioester **4** and Cys‐peptide **5** catalyzed by MPAA^[31]^ and the subsequent deprotection of thiazolidine‐98 were performed in a one‐pot approach^[32]^ furnishing the EPO 98–166 peptide **26** in 89 % yield. Glycopeptide hydrazide **23** was ligated with equimolar amounts of EPO 29–67 glycopeptide thioester **21** giving EPO 29–97 glycopeptide hydrazide **25** in 72 % yield after purification. Conversion of the glycopeptide hydrazide **25** to the corresponding thioester **S14** required strict pH‐control, to avoid the formation of an unreactive lactam at the C‐terminal lysine. The lactamization can be avoided efficiently by use of side chain protecting groups^[33]^ or by lowering the pH during thiolysis of the acyl azide.^[8d]^ Cys‐peptide **26** was ligated with the glycopeptide thioester **S14** furnishing the EPO glycopeptide 29–166 **27** in high yield (73 %). A simultaneous radical desulfurization^[14]^ of the three thiol groups using VA‐044/TCEP proceeded best with glutathione^[34]^ as a thiol additive and gave quantitative conversion to **28** within 3 h. The reaction progress was monitored by RP‐HPLC‐MS since the partially desulfurized species showed no difference in retention time on HPLC.

At this point the cleavage of the Acm groups of **28** was investigated. With respect to the experiences obtained from the inital syntheses we considered the use of aqueous Pd^II[35]^ to remove the three Acm groups as an alternative to commonly applied Ag^I^.^[8]^ To our delight the Acm groups were rapidly cleaved by PdCl_2_ in water. However, due to the poor solubility of PdCl_2_ in water the results were not consistent and the deprotected glycopeptide retained a brownish color after purification by HPLC. By adding NaCl to the suspension of PdCl_2_ in water the well‐soluble tetrachloropalladate complex formed leading to quantitative cleavage of the Acm groups with stoichiometric amounts of Pd^II^. Subsequently, the addition of a large excess of DTT was required to fully remove bound Pd^[36]^ from the thiol groups of the deprotected glycopeptide as evidenced by the formation of colored, soluble Pd‐DTT‐chelates.^[37]^ The use of other scavengers for Pd^II^ (dimethylglyoxime^[38]^ or 3‐mercaptopropionic acid^[39]^) was less efficient. Although the relative amount of Pd^II^ had to be increased to 30 equivalents when carrying out the deprotection of **28** in buffered guanidinium chloride, the subsequent removal of bound Pd with excess DTT proceeded smoothly under these conditions. Residual palladium led to poor ionization of the deprotected glycopeptide **29** under LC‐MS conditions (Figure S31‐Pd). After purification by RP‐HPLC the desired EPO 29–166 glycopeptide **29** was obtained as a white solid in 83 % yield. The buffered deprotection conditions avoid the acidic pH of aqueous solutions of palladium chloride and should thus also be compatible with acid‐sensitive sialylated EPO glycopeptides.

The EPO 29–166 glycopeptide **29** was subsequently ligated with the EPO 1–28 glycopeptide thioester **18**. After 6 d of reaction time high conversion to the full‐length glycopeptide **30** was observed, which was directly subjected to refolding by dialysis of the ligation mixture.[^7a^, ^8c^] Arginine was added to suppress the aggregation of folding intermediates.^[40]^ Monomeric folded **EPO A** was separated from misfolded and oligomeric EPO species by size exclusion chromatography (SEC) and purified further by RP‐HPLC. The purity of the refolded **EPO A** was confirmed by SDS‐PAGE, HPLC‐MS and high resolution ESI‐MS. The gaussian charge state distribution in the mass spectrum indicates conformational homogeneity of the sample. Moreover, the CD‐spectrum of **EPO A** was nearly identical to that reported for recombinant EPO.^[41]^ The residual Pd‐content of the synthetic **EPO A** was determined by inductively coupled plasma optical emission spectrometry (ICP‐OES) and remained below the detection limit of 0.01 ppm.

**Scheme 5:**
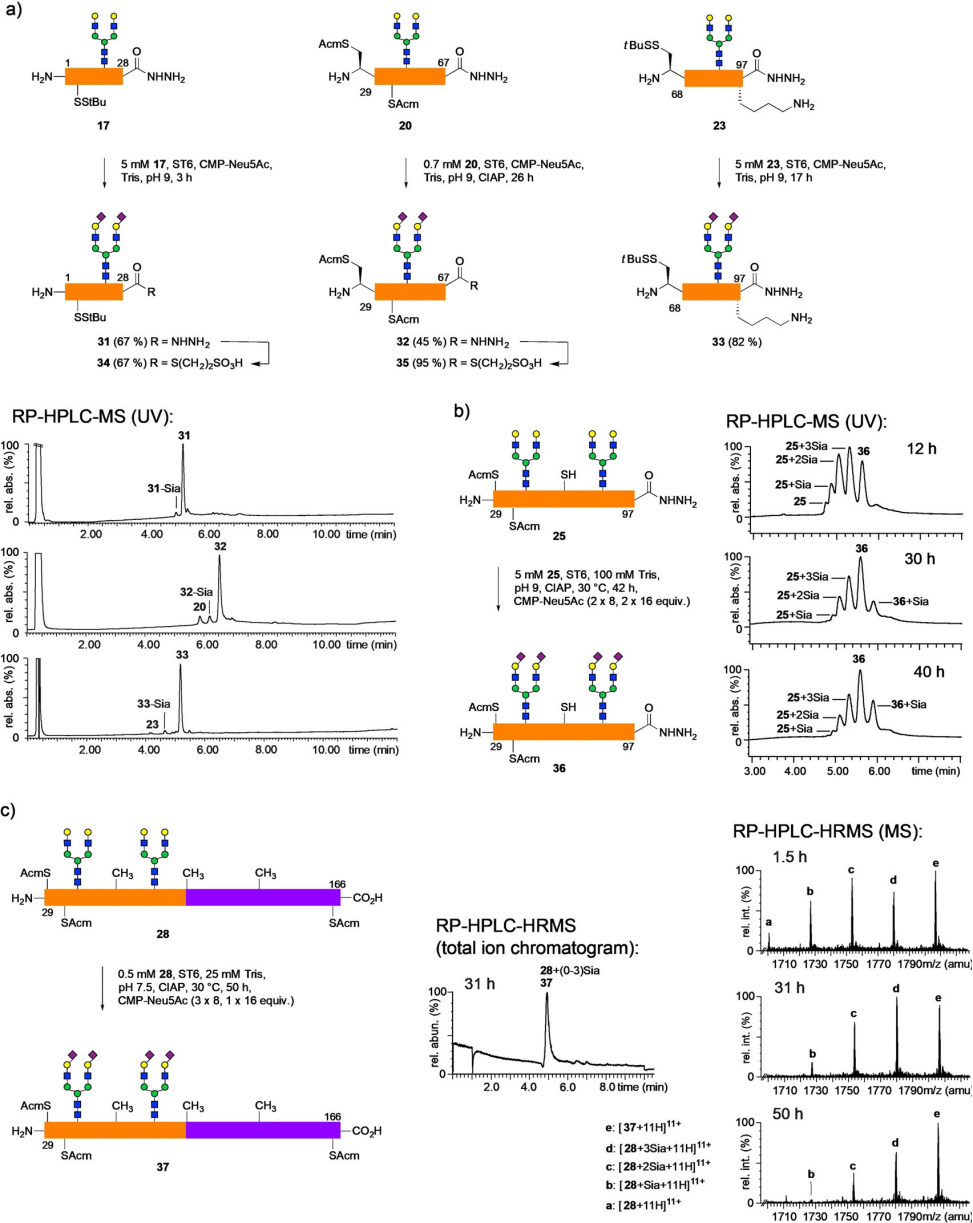
The efficiency of the enzymatic 2,6‐sialylation of EPO glycopeptides decreases with increasing chain length: a) sialylation of 30–40mer glycopeptide hydrazides **17**, **20**, **23** and RP‐HPLC‐MS of crude mixtures; b) sialylation of EPO 29–97 glycopeptide **25** and RP‐HPLC‐MS of crude mixtures; c) sialylation of EPO 29–166 glycopeptide **28**; Total Ion Chromatogram (TIC) of reaction mixture RP‐HPLC‐HRMS after 31 h; RP‐HPLC‐HRMS of reaction mixtures after different reaction times (only 11+ charged peaks of MS are shown).

We next investigated the enzymatic sialylation of the various glycopeptide hydrazides used in the synthesis of **EPO A** (Scheme 5). The α‐2,6‐sialyltransferase from *Photobacterium damsela* (ST6)^[42]^ readily catalyzed the transfer of sialic acid to the terminal galactose residues of the biantennary *N*‐glycopeptide hydrazides **17**, **20** and **23** in concentrations ranging from 0.7–5 mM. Alkaline phosphatase (CIAP) was added to reduce product inhibition^[43]^ and sialidase activity of the bacterial enzyme.^[44]^ After repeated addition of CMP‐Neu5Ac the conversions to the disialylated derivatives typically exceeded 90 %. To prevent loss of the acid‐sensitive 2,6‐sialosides in the course of preparative HPLC, TFA was replaced by formic acid as an additive and the eluates were immediately neutralized with dilute Na_2_CO_3_. After a final desalting step, the sialoglycopeptide hydrazides were obtained in yields of 67 % (**31**), 45 % (**32**) and 82 % (**33**).

Despite the presence of the same biantennary *N*‐glycan the reactivity (rate of conversion) and the overall reaction times of the three glycopeptide acceptors **17**, **20** and **23** (28–39 amino acids) varied strongly. All glycopeptides were well‐soluble in water even at a concentration of 5 mM. EPO 1–28 glycopeptide **17** already reached nearly full conversion after 3 h whereas EPO 68–97 (**23**) required 16 h of reaction time. Remarkably, EPO 29–67 (**20**, 5 mM) gave only 52 % conversion to **32** after 16 h. By varying the reaction parameters (see Table S7) it was found that at lower concentrations (0.5–1 mM **20**) the sialylation proceeded significantly better leaving less than 10 % of incompletely sialylated compounds.

It can be assumed that the reactivity of the *N*‐glycans in the three glycopeptide segments **17**, **20** and **23** is affected by several factors. The solubility in neat water and buffers is good for all three fragments. Additionally, their hydrophilicity is quite similar indicated by a close range of retention times on an RP‐18 HPLC‐column (see Scheme S39a). For more insight into the secondary structure of the glycopeptides in solution we recorded CD spectra in dilute sialylation buffer (50 μM glycopeptide in 5 mM Tris, pH 9) (Scheme S39a). The two segments **17** and **23** displaying the highest reactivity of the *N*‐glycan showed CD spectra with defined peaks suggesting a random coil.^[45]^ Glycopeptide **20** gave a similar CD spectrum, albeit less defined and with multiple small peaks, which might reflect conformational deviations from the typical random coil of segments **17** and **23**. Thus, CD spectra can provide a starting point to investigate the different reactivities of the three glycopeptides with respect to the peptide part. Most likely the local conformation of the peptide regions flanking the *N*‐glycans affect the accessibility by the enzyme. We assume that the secondary structures found in EPO should also be present in the corresponding segments to a certain extent. In the most reactive segment **17** the *N*‐glycan (Asn 24) is located at the end of the complete N‐terminal helix of the protein. In the less reactive segment **23** the *N*‐glycan (Asn 83) is placed between two incomplete α‐helices connected by a short loop (7 aa). In the least reactive segment **20** the *N*‐glycan (Asn 38) is within an extended loop region (19 aa) and directly flanked by a short β‐sheet. We assume that an unfavorable combination of local conformation and aggregation in the reaction buffer lowers the reactivity of **20**. Revealing the explicit mechanisms, which govern the different reactivities of the EPO glycopeptides **17**, **20** and **23** during enzymatic sialylation requires an in‐depth study and is beyond the scope of this paper.

The enzymatic sialylation was also tested with longer glycopeptides bearing two *N*‐glycans. Multiple sialic acid residues were transferred onto the well‐soluble glycopeptides EPO 29–97 **25** and EPO 29–166 **28**, but despite repeated additions of excess CMP‐Neu5Ac the conversions were generally sluggish and incomplete. In the case of hydrazide **25** the large excess of CMP‐Neu5Ac led to an oversialylation of the glycopeptide (**36**+Sia, Scheme 5 b), which presumably occurred at the hydrazide function. Despite the length of glycopeptide **25** the different sialylation products were chromatographically resolved (RP‐HPLC‐MS). We assumed that the free thiol group of **25** might interfere with the reaction progress and thus tested the desulfurized 29–166 glycopeptide **28**. However, the starting material **28** and the various sialylation products eluted in a single peak during LC‐MS analysis. Thus, the reaction progress was estimated from the relative intensity of the MS peaks of the various sialylation products after HPLC‐HRMS analysis (Scheme 5 c). The reactivity of **28** was marginally lower compared to **25**.

**Scheme 6:**
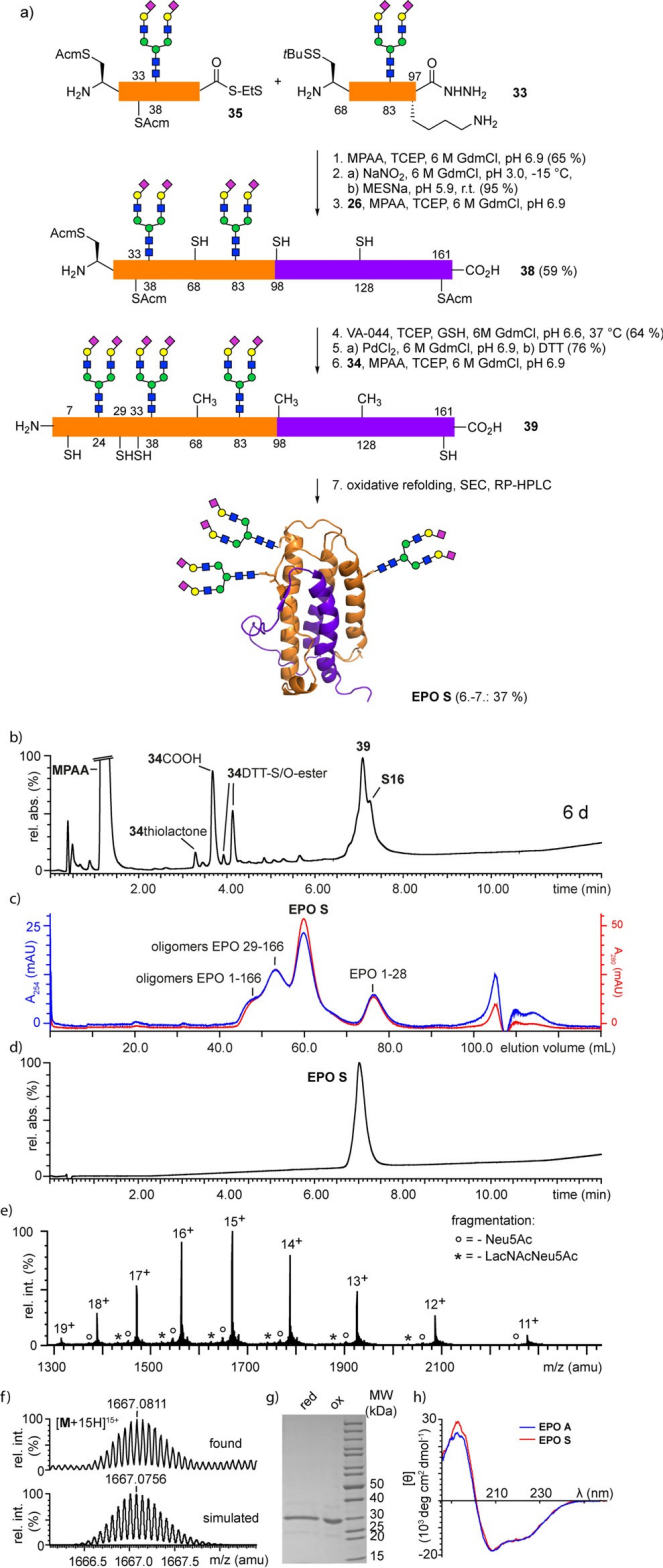
a) Synthesis of glycoform **EPO S** from sialylated glycopeptides; b) RP‐HPLC‐MS of the ligation of sialylated glycopeptide thioester **34** and Cys‐glycopeptide 29–166 **S 16** to full‐length EPO 1–166 **39** after 6 d, c) SEC after oxidative refolding; d) RP‐HPLC‐MS of **EPO S** after RP‐HPLC purification; e) HR‐MS of purified **EPO S** (H_2_O, direct injection); f) simulated and measured isotope pattern of [**M**+15 H]^15+^ HR‐MS peak; g) SDS‐PAGE (reduced and oxidized) of **EPO S**. h) CD‐spectra of **EPO A** and **EPO S**.

Since the complete enzymatic sialylation of longer EPO glycopeptides with multiple glycans was not promising, the more readily accessible 2,6‐sialylated glycopeptides **31**, **32** and **33** were used for assembling a sialylated EPO glycoform by sequential ligations following the procedures established for **EPO A** (Scheme 6). The glycopeptide hydrazides **31** and **32** were converted to the corresponding thioesters **34** and **35**. Diazotization of the glycopeptides at pH 3 (−15 °C) did not affect the sialosides. The ligation of the sialoglycopeptide thioester **35** with glycopeptide **33** proceeded smoothly (65 % yield) and the resulting hydrazide **36** was efficiently converted to the corresponding thioester **S15** maintaining pH 5.9 in the thiolysis step^[8d]^ to avoid lactamization of the C‐terminal lysine. Despite a good conversion in the following ligation with **26** the 29–166 glycopeptide **38** was difficult to purify owing to a similar retention of **38** and the hydrolysis product of thioester **35**. The subsequent desulfurization step and the removal of the Acm groups with Pd^II^ gave lower yields compared to the non‐sialylated compounds. This trend continued in the final ligation where the conversion to the sialylated full‐length EPO 1–166 glycopeptide **39** was lower than for **30**. As a consequence, the oxidative refolding of the ligation mixture resulted in a higher proportion of oligomeric EPO 29–166 relative to oligomeric EPO 1–166 in the purification of **EPO S** by size exclusion chromatography (Scheme 6 b, c). After preparative RP‐HPLC the desired sialylated glycoform **EPO S** was obtained in 37 % yield. **EPO S** was characterized by SDS‐PAGE, HPLC‐MS, HR‐ESI‐MS and CD‐spectroscopy (Scheme 6 d–h). The purity and the CD spectrum of **EPO S** were nearly identical to that of **EPO A** indicating that the synthetic approach to EPO is robust and applicable to non‐sialylated as well as sialylated glycoforms.

**Scheme 7:**
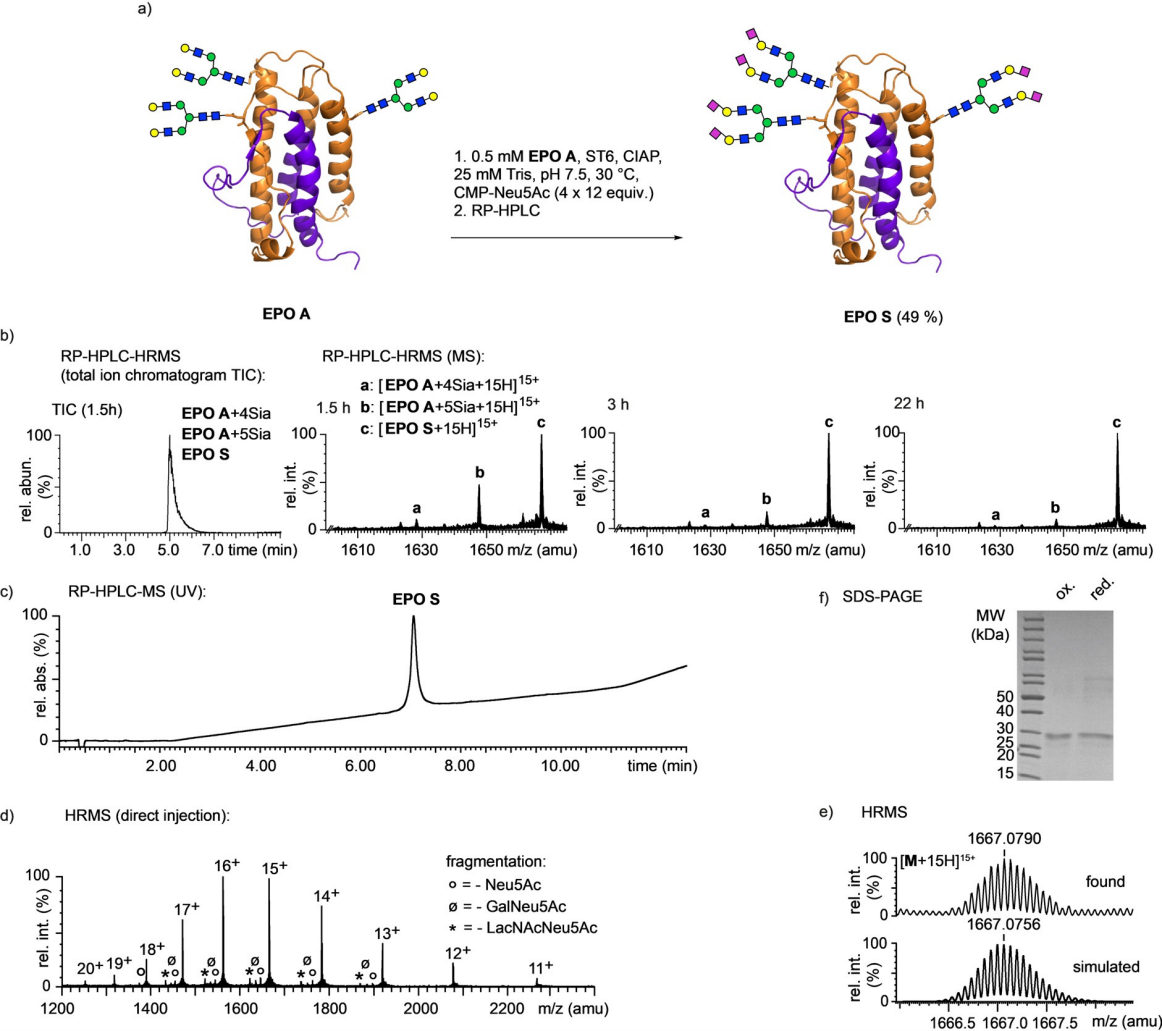
a) The enzymatic sialylation of **EPO A** yields the sialylated glycoform **EPO S** directly; b) RP‐HPLC‐HRMS of the enzymatic sialylation of **EPO A** after 22 h, sialylation intermediates coelute with product **EPO S**: TIC of LC‐MS (1.5 h) and MS after different reaction times are shown; c) RP‐HPLC‐MS of **EPO S** after RP‐HPLC purification; d) HR‐MS of purified **EPO S** (H_2_O, direct injection); e) simulated and measured isotope pattern of [**M**+15 H]^15+^ HR‐MS peak; f) SDS‐PAGE of **EPO S** (reduced and oxidized).

Finally, we also attempted an enzymatic 2,6‐sialylation of the synthetic glycoform **EPO A** (Scheme 7). In contrast to the unfavorable acceptor properties of the longer glycopeptides **25** and **28**, the folded glycoprotein **EPO A** showed fast conversion. At an acceptor concentration of 0.5 mM corresponding to 11.5 mg of **EPO A** mL^−1^ the transfer of six sialic acid units could be driven to completion by multiple additions of CMP‐Neu5Ac. The conversion could only be followed by MS (Scheme 7 b) as all sialylation intermediates of EPO coeluted on RP‐HPLC (Scheme 7 b, TIC of LC‐MS). After purification by RP‐HPLC the glycoform **EPO S** was obtained in good yield and high purity. The glycoprotein was virtually identical to **EPO S** synthesized independently from the sialylated glycopeptides. We observed the same low degree of desialylation and other fragmentations of the *N*‐glycans during HRMS analysis of **EPO S** made from sialylated glycopeptides and **EPO S** obtained by enzymatic sialylation of **EPO A** (see Schemes 6 e, 7 d). This indicates that the minor MS‐peaks corresponding to **EPO S** with loss of Neu5Ac, GalSia and LacNAcSia mainly originate from fragmentation during HRMS analysis rather than incomplete enzymatic sialylation of **EPO A**.

The unexpectedly high reactivity of the bacterial 2,6‐sialyltransferase towards the folded **EPO A** glycoprotein provides rapid access to the sialylated glycoform **EPO S** in a single step. This straight‐forward transformation bypasses the need to synthesize the three sialoglycopeptide building blocks **33**–**35** and carry them through a time‐consuming process involving multiple ligations, peptide modifications and protein refolding.

**EPO A** and the unfolded glycopeptides **25** and **28** are well‐soluble in aqueous buffers but show different reactivities with the bacterial sialyltransferase. We assume that the faster enzymatic sialylation of properly folded **EPO A** should mainly be attributed to a generally good accessibility of the three *N*‐glycans on the surface of the globular glycoprotein. In the case of the structurally less reinforced EPO fragments **25** and **28** unfavorable local conformations around each glycosylation site most likely affect the sialylation reactions and hamper full conversion.

The observed high efficiency of the late‐stage enzymatic sialylation of **EPO A** is very encouraging. Most likely other homogeneous glycoproteins can also be modified analogously, however, the feasibility and efficiency of this approach need to be evaluated on a case‐to‐case basis. The efficient enzymatic α‐1,4‐galactosylation^[46]^ of a folded *N*‐glycoprotein was recently shown using a synthetic glycoform of Saposin D containing a biantennary *N*‐glycan.^[16]^ From our experiences well‐behaved proteins are preferred substrates since they readily withstand the conditions of the enzymatic reactions and the following purifications leading to good overall yields. It is desirable that the enzymatic conversion of the homogeneous glycoprotein reaches completion unless reliable chromatographic means for the efficient removal of products resulting from incomplete reactions are available.

**Scheme 8:**
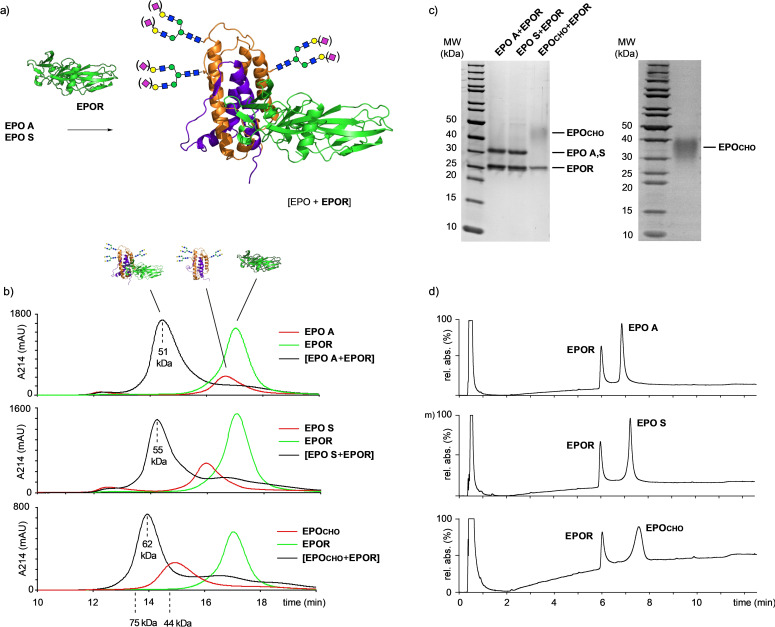
a) Cartoon of the high affinity (1:1) complexes of **EPO A** and **EPO S** with **EPOR** based on a crystal structure (PDB code: 1EER); b) complex formation of **EPO A**, **EPO S** and **EPO_CHO_** with **EPOR** monitored by SEC (apparent molecular weights of the complexes were calculated by linear approximation between the reference points of 75 and 44 kDa given by the manufacturer); c) SDS‐PAGE (non‐reducing) of the complexes of **EPO A**, **EPO S** and **EPO_CHO_** with **EPOR** isolated by SEC; d) the isolated complexes of **EPO A**, **EPO S** and **EPO_CHO_** with **EPOR** were first dissociated with 0.2 % TFA and subsequently analyzed by RP‐HPLC‐MS.

To ensure that synthetic **EPO A** and **EPO S** are biologically recognized we investigated binding to the human EPO receptor **EPOR** (Scheme 8). The extracellular domain (25–250) of the receptor^[47]^ was expressed as a His_6_‐SUMO^[48]^ fusion protein in *E. coli* and gave inclusion bodies^[49]^ (see supporting information). After purification of the fusion protein by Ni‐IMAC the SUMO domain was cleaved using the specific protease SENP2. Subsequently, **EPOR** was refolded from urea^[49]^ by dialysis and purified via gel filtration. The **EPOR** was characterized by SDS‐PAGE, HPLC‐MS, HR‐MS, and CD spectroscopy. The CD data are consistent with a previous report^[50]^ suggesting a proper fold of **EPOR**.

EPO can bind to its receptor via a high affinity and a low affinity site.^[51]^ In solution the high affinity complex between EPO and the soluble EPO receptor (1:1 ratio) is formed first. Only at relatively high receptor concentrations (≈8 mg of EPOR mL^−1^) binding of an additional EPOR occurs, leading to the 2:1 EPOR/EPO complex, which can be detected by gel filtration.^[51]^

We first probed the binding of the refolded **EPOR** (24.8 kDa) with commercially available EPO recombinantly expressed in CHO cells (**EPO_CHO_** ≈37 kDa). Both proteins were mixed under dilute conditions (≈0.3 mg **EPOR** mL^−1^) and submitted to gel filtration over an analytical superdex 75 column. A newly formed peak (apparent MW≈62 kDa) eluting earlier than the individual proteins indicated the formation of an **EPO_CHO_** +**EPOR** complex. Analysis of the peak by SDS‐PAGE clearly showed **EPOR** but **EPO_CHO_** was visible only as a broad band due to *N*‐glycan microheterogeneity.[^3^, ^52^] Thus, the **EPO_CHO_** +**EPOR** complex was dissociated using dilute TFA (0.2 %, 1 h) followed by an RP‐HPLC‐MS analysis.[^48^, ^53^] The relative intensity of the two protein peaks corresponded to the formation of the 1:1 complex. Subsequently, the complexation of the synthetic glycoforms **EPO A** and **EPO S** with **EPOR** was carried out and confirmed by gel filtration (complexes with an apparent MW of ≈51 or ≈55 kDa) and SDS‐PAGE. In both cases the formation of 1:1 EPO/**EPOR** complexes was revealed by RP‐HPLC‐MS after dissociation of the complexes with dilute TFA. These findings altogether indicate that the EPO receptor **EPOR** binds to the synthetic glycoforms **EPO A**, **EPO S** and to recombinant **EPO_CHO_** in a similar manner. Thus, synthetic **EPO A** and **EPO S** should display the biologically relevant native fold.

## Conclusion

In summary, we developed a robust and flexible chemical synthesis for EPO bearing three biantennary *N*‐glycans based on sequential native chemical ligation. Out of several protecting group schemes only one strategy rendered EPO reproducibly and in high purity. For glycopeptide segments containing Acm‐protected cysteines the use of a PhiPr ester instead of an allyl ester at the glycosylation site was instrumental. The final cleavage of Acm groups with Pd^II^ was found to be compatible with glycopeptides bearing multiple *N*‐glycans. Despite the use of excess Pd^II^ the residual content of Pd in the final glycoprotein **EPO A** was below the detection limit of 0.01 ppm. Sialic acids were conveniently introduced at the level of the glycopeptide hydrazide building blocks, which could be elaborated to the sialylated glycoform **EPO S**. A surprisingly efficient alternative approach to sialylated EPO was found in the enzymatic sialylation of **EPO A** furnishing the sialylated glycoform **EPO S** in a single step. The enzymatic modification of the *N*‐glycans of folded EPO should provide rapid access to multiple glycoforms from the same precursor. Biological recognition of synthetic **EPO A** and **EPO S** was shown to be similar to commercially available **EPO_CHO_** by complexation with recombinant human EPO receptor **EPOR**. The chemoenzymatic routes presented herein should facilitate the synthetic access to even more complex glycoforms of EPO, thus providing the well‐defined tools needed to evaluate how carbohydrates affect the critical plasma lifetime of this circulatory cytokine therapeutic.

## Conflict of interest

The authors declare no conflict of interest.

## Supporting information

As a service to our authors and readers, this journal provides supporting information supplied by the authors. Such materials are peer reviewed and may be re‐organized for online delivery, but are not copy‐edited or typeset. Technical support issues arising from supporting information (other than missing files) should be addressed to the authors.

Supporting Information
