# Supplementary material for: Chemical and Enzymatic Synthesis of Sialylated Glycoforms of Human Erythropoietin
Source: Angew Chem Int Ed Engl. 2021 Nov 2;60(49):25922–32. doi: 10.1002/anie.202110013 (PMC9297946; doi:10.1002/anie.202110013)
Supplement: Supplementary file 1 — Supporting Information [file ANIE-60-25922-s001.pdf]

Angewandte Chemie
Eine Zeitschrift der Gesellschaft Deutscher Chemiker

Supporting Information

Chemical and Enzymatic Synthesis of Sialylated Glycoforms of Human Erythropoietin

*Hendrik Hessefort, Angelina Gross, Simone Seeleithner, Markus Hessefort, Tanja Kirsch, Lukas Perkams, Klaus Ole Bundgaard, Karen Gottwald, David Rau, Christopher Günther Franz Graf, Elisabeth Rozanski, Sascha Weidler, and Carlo Unverzagt**

anie_202110013_sm_miscellaneous_information.pdf

1. General Methods
2. General Synthesis Procedures
3. Synthesis of Glycopeptide Hydrazides
3.1 Synthesis of EPO 29-67 Hydrazide **19**
3.2 Selective Deprotection of EPO 29-67 Hydrazide **19**
3.3 Synthesis of EPO 68-97 Hydrazide **22**
3.4 Selective Deprotection of EPO 68-97 Hydrazide **22**
3.5 Synthesis of EPO 29-67 Glycopeptide Hydrazide **20**
3.6 Synthesis of EPO 68-97 Glycopeptide Hydrazide **23**
4. Synthesis of the C-terminal Fragments
4.1 Synthesis of EPO 98-127 Thioester **4**
4.2 Synthesis of EPO 128-166 Peptidyl Acid **5**
5. Synthesis of EPO A by Sequential NCL
5.1 Synthesis of EPO 29-67 Glycopeptide Thioester **21**
5.2 Synthesis of EPO 29-97 Glycopeptide Hydrazide **25**
5.3 Synthesis of EPO 29-97 Glycopeptide Thioester **S14**
5.4 Synthesis of EPO 98-166 **26**
5.5 Synthesis of EPO 29-166 Glycopeptide **27**
5.6 Desulfurization of EPO 29-166 Glycopeptide **27**
5.7 Cleavage of Acm groups from EPO 29-166 Glycopeptide **28**
5.8 Synthesis of EPO 1-28 Glycopeptide Thioester **18**
5.9 Synthesis and Refolding of **EPO A**
6. Synthesis of Sialylated EPO S by Sequential NCL
6.1 Synthesis of Sialylated EPO 1-28 Glycopeptide Hydrazide **31**
6.2 Synthesis of Sialylated EPO 29-67 Glycopeptide Hydrazide **32**
6.3 Synthesis of Sialylated EPO 68-97 Glycopeptide Hydrazide **33**
6.4 Synthesis of EPO 1-28 Sialoglycopeptide Thioester **34**
6.5 Synthesis of EPO 29-67 Sialoglycopeptide Thioester **35**
6.6 Synthesis of EPO 29-97 Sialoglycopeptide Hydrazide **36**
6.7 Synthesis of EPO 29-97 Sialoglycopeptide Thioester **S15**
6.8 Synthesis of EPO 29-166 Sialoglycopeptide **38**
6.9 Desulfurization of EPO 29-166 Sialoglycopeptide **38**
6.10 Cleavage of Acm groups from EPO 29-166 Sialoglycopeptide **37**
6.11 Synthesis and Refolding of **EPO S**
7. Enzymatic Sialylation of Glycopeptides
7.1 Sialylation of EPO 29-97 Glycopeptide Hydrazide **25**
7.2 Sialylation of EPO 29-166 Glycopeptide **28**
7.3 Preparative Sialylation of **EPO A**
8. Expression of EPOR and Complexations with EPO Glycoforms
8.1 Overexpression and Purification of **EPOR**
8.2 Refolding and Purification of **EPOR**
8.3 Complexation of EPO glycoforms with **EPOR**

1. General Methods

Solvents were dried according to standard methods. Automated Fmoc-SPPS was performed in 45-mL reaction vessels in a PTI Tribute peptide synthesizer with UV-monitoring and feedback control system. Manual SPPS steps were performed in 2-mL, 5-mL, 10-mL or 20-mL polypropylene syringes equipped with a 25 µm polyethylene filter (Multisyntech, Germany). Fmoc or Boc protected amino acids and coupling reagents were obtained from Novabiochem (Läufelfingen, Switzerland), Iris Biotech (Marktredwitz, Germany) and Sigma Aldrich (Taufkirchen, Germany). 4-(Mercaptomethyl)-benzoic acid (MMBA),[1] and GlcNAc-NH_2[2] were synthesized according to published procedures. Trityl-ChemMatrix resin (0.30 mmol/g loading, 100-200 mesh dry) was obtained from PCAS BioMatrix Inc., Canada).

ESI-TOF mass spectra were recorded on a Micromass LCT instrument coupled to an Agilent 1100 HPLC or a Waters ACQUITY UPLC H Class System with a photodiode array detector using solvent A (H_2O + 0.1 % HCOOH) and B (MeCN + 0.1 % HCOOH). HR-ESI mass spectra were recorded on a Thermo Q Exactive Orbitrap mass spectrometer. Separations were carried out using [A] YMC-UltraHT Hydrosphere C18 (30 x 2 mm, 2 µm, 120 Å), [B] YMC-UltraHT Hydrosphere C18 (50 x 2 mm, 2 µm, 120 Å), [C] YMC-Triart C8 (30 x 2 mm, 1.9 µm, 120 Å), [D] Supelco Discovery BIO Wide Pore C5 (50 x 2.1 mm, 3 µm, 300 Å), [E] Supelco Discovery BIO Wide Pore C5 (50 × 2.1 mm, 5 µm, 300 Å).

Preparative RP-HPLC was performed on an Äkta Basic HPLC with UV-detection at 214, 254 and 280 nm using solvent C (H_2O + 0.1 % TFA) and D (MeCN + 0.1 % TFA). The following columns were used: [F] YMC Pack Pro C8 (250 × 20 mm, 5 µm, 120 Å), [G] Supelco Ascentis C18 (150 × 10 mm, 5 µm, 100 Å), [H] Supelco Discovery BIO Wide Pore C5 (150 × 4.6 mm, 5 µm, 300 Å), [I] Supelco Discovery BIO Wide Pore C5 (150 × 10 mm, 5 µm, 300 Å), [J] YMC Pack Protein RP C4 (150 × 10 mm, 5 µm, 200 Å), [K] YMC Triart C8 (150 × 10 mm, 5 µm, 120 Å), [L] YMC Hydrosphere C18 (150 × 10 mm, 5 µm, 120 Å).

Preparative size exclusion chromatography was performed on an Äkta Purifier HPLC with conductivity detection and UV-detection at 214, 254 and 280 nm using the following columns: [M] Sephadex G-25 (164 × 15 mm, 20 – 50 µm), [N] Sephadex G-25 (155 × 25 mm, 20 – 50 µm), [O] GE Healthcare Superdex Peptide 10/300 GL (310 × 10 mm, 13 µm), [P] GE Healthcare Superdex 75 prep grade 16/600 (600 × 16 mm, 34 µm).

Flash chromatography was performed on a GRACE Reveleris® iES-flash chromatography system with ELS-detector. Thin layer chromatography was performed on coated aluminum plates (silica gel 60 GF_{254}, Merck Darmstadt). Spots were detected by UV light or by charring with a 1:1 mixture of 2 N H_2SO_4/0.2 % resorcinol monomethyl ether in ethanol. Dialysis tubing

Zellutrans V Serie (MW cutoff: 5000, flat width 40 mm) was obtained from Roth (Germany).
CD spectra were recorded on a Jasco J-715 spectropolarimeter. A 1 mm cuvette (110-QS, Hellma Analytics, fused silica Suprasil) was used. The ellipticities ΔΘ were corrected by a matrix blank and were normalized as residual molar ellipticities $[\Theta]_{MRW}$: $[\Theta]_{MRW}$ (deg cm^2 $dmol^{-1}$) = ΔΘ (mdeg) / (l (mm) × [**EPO A**] (mol/L) × (N_{aa} - 1)). l is the layer thickness of the cuvette and N_{aa} is the number of amino acid residues in the protein.
ICP-OES was measured on an Optima 7300 DV from PerkinElmer with a Meinhard atomizer.
CMP-Neu5Ac was kindly provided by Roche Diagnostics GmbH as sodium salt.
α-2,6-Sialyltransferase from Photobacterium damsela (Δ15PD2,6ST)[3] was obtained from Sigma Aldrich. Alkaline phosphatase from calf intestine was purchased from Sigma Aldrich (10.3 mg/mL with 2145 U/mg, Buffer: 30 mM triethanolamine, pH 7.6, 3 M NaCl, 1 mM $MgCl_2$, 0.1 mM $ZnCl_2$).
The degree of loading of resins was determined by Fmoc cleavage of the penultimate amino acid.[4,5] Absorbance measurements were performed on a Specord 2000 spectrophotometer from Analytik Jena. Analytical TFA deprotections were performed by adding 0.5 mg of resin or 0.2 mg of protected peptide to 100 µL of TFA/TIS/H_2O (95:2.5:2.5) for 60 min. The mixture was dried in high vacuum, the residue was dissolved in MeCN/H_2O + 0.1 % HCOOH and analyzed by HPLC-MS.

2. General Synthesis Procedures

Automated Fmoc-SPPS

Solid-phase synthesis following the Fmoc strategy[6] was performed automatically in 45 mL reaction vessels on a Tribute peptide synthesizer with IntelliSynth UV Monitoring and Feedback Control System from Protein Technologies Inc. All reactions were performed at ambient temperature under a nitrogen atmosphere and mechanical shaking. Nitrogen was passed through the reaction suspension for additional mixing. Before starting the synthesis, the resin was swollen by washing with CH_2Cl_2 (5 x 30 s) and DMF (5 x 30 s).

Automated Removal of N^{α}-Fmoc

To remove *N*-terminal Fmoc protection a piperidine solution (20 % in DMF) was added to the resin and shaken for 0.5 min. The coupled UV monitoring system of the peptide synthesizer initiated further Fmoc cleavage cycles if the absorbance (A_{301}) of the filtrate indicated incomplete cleavage. After complete Fmoc removal, the resin was washed with DMF (5 x 30 s).

Automated Coupling of Fmoc amino acid derivatives

The amino acid building block and the activation reagent were taken up in a defined volume of DIPEA in DMF (for molarity see specific synthesis) before coupling, mixed for 2 min, and added to the deprotected peptidyl resin. Cysteine derivatives were activated as symmetric anhydrides and coupled manually. At the end of each coupling the resin was washed with DMF (5 x 30 s). The exact coupling conditions are given in the specific synthesis protocols.

Manual coupling of cysteine derivatives[7]

In dried glassware under argon, the cysteine building block (10 equiv.) was dissolved in dry DMF/CH_2Cl_2 1:2.5 (*c* Fmoc/Boc-Cys(PG)-OH = 180 mM) and *N*,*N*'-diisopropylcarbodiimide (5 equiv.) was added at 0 °C. The reaction mixture was stirred at 0 °C for 5 min and for 30 min at ambient temperature. Subsequently, the CH_2Cl_2 was removed under vacuum. The suspension was drawn up into a syringe with and added to the deprotected peptidyl resin swollen in DMF. The residue and the vessel were washed three times with an appropriate volume of DMF to give a concentration of 105 mM for the anhydride in the coupling solution. After 3 - 5 h of shaking at ambient temperature, the coupling solution was removed and the resin was washed with CH_2Cl_2 and DMF (five times each).

Mild acidic cleavage of protected peptidyl acids from trityl resin

To cleave protected peptidyl acids from *p*-carboxytrityl-ChemMatrix resin the peptidyl resin was swelled in CH_2Cl_2 in a syringe reactor for 20 min. Subsequently 20 % HFIP in CH_2Cl_2 (approx. 8 mL/g resin) was added and the suspension was shaken at room temperature for 1 min. The resin was treated seven times for 1 min each with the same volume of 20 % HFIP solution and finally washed five more times with CH_2Cl_2. All the cleavage and washing solutions were collected in a cooled flask (0 °C) containing 25 times the volume of CH_2Cl_2 used for a single cleavage. The combined cleavage solutions were concentrated under vacuum at 4 °C bath temperature. HFIP was azeotropically removed by adding CH_2Cl_2 several times. The residue was dried *in vacuo* and lyophilized from dioxane.

Conversion of glycopeptide hydrazides to thioesters

The glycopeptide hydrazide (1.0 equiv.) was dissolved in GdmCl buffer (6 M GdmCl, 0.2 M NaH_2PO_4, pH 3.0) to a final concentration of 3.5 mM and stirred for 15 min at -15 °C. Subsequently, 0.5 M $NaNO_2$ (6.5 eq) was added under stirring. After 1 h at -15 °C MESNa (65 equiv., 1.0 M in 6 M GdmCl/0.2 M NaH_2PO_4) was added. The pH value of the MESNa solutions

was set to 6.6 for EPO 29-67 glycopeptides **21** and **35** and to pH 6.0 for EPO 29-97 glycopeptides **S14** and **S15**. The concentration of the acyl azide in the thiolysis mixture was 2.75 mM. Once the reaction mixture reached ambient temperature a microelectrode was used to readjust the pH using 6 M and 1 M NaOH. At the desired pH the reaction mixture was stirred for 1 h with the microelectrode immersed. Subsequently, the microelectrode and the magnetic stir bar were rinsed with 100 µL of phosphate buffer (0.2 M NaH_2PO_4/6 M GdmCl/pH 3.0). For sialylated compounds 0.2 M NaH_2PO_4/6 M GdmCl/pH 6.6 (EPO 29-67 glycopeptide **35**) or pH 6.0 (EPO29-97 glycopeptide **S15**) were used in the rinsing step. The combined reaction mixtures were desalted by gel filtration.

3. Synthesis of Glycopeptide Hydrazides

3.1 Synthesis of EPO 29-67 Hydrazide 19

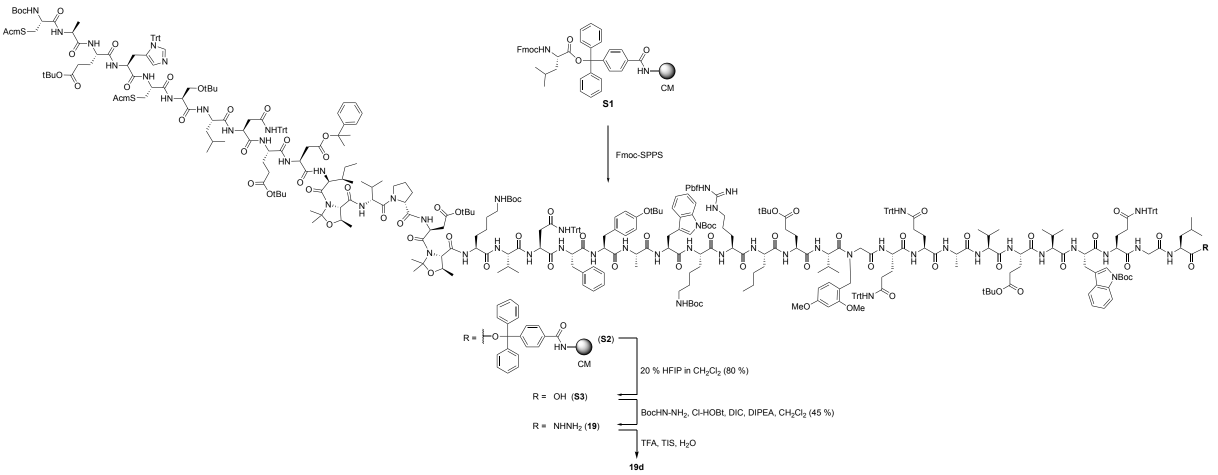

Figure S1: Synthesis of EPO 29-67 hydrazide **19**

Fmoc-Leu-Trt-CM resin **S1** (loading = 276.6 µmol/g) was synthesized according to Ref.[8]. 462.8 mg (128 µmol, 1 eq) of resin **S1** were placed in a 45 mL peptide synthesizer reaction vessel and the peptide chain was elongated automatically. For cleavage of the Fmoc group the general procedure was applied and the amino acid building blocks were coupled under the conditions denoted in table S1 and the general procedures section. After complete elongation the resin **S2** was washed with DMF (5 x) and CH_2Cl_2 (5x) and was dried *in vacuo*.

Table S1:

Position	Building Block	*m* (mg)	*V* (mL) 0.19 M DIPEA in DMF	Coupling time (min)
Gly-66	Fmoc-Gly-OH[a]	190.3	7.0	45
Gln-65	Fmoc-Gln(Trt)-OH[a]	390.9	7.0	45

Trp-64	Fmoc-Ala-OH[a]	337.0	7.0	45
Val-63	Fmoc-Val-OH[a]	217.2	7.0	45
Glu-62	Fmoc-Glu(OtBu)-OH[a]	272.3	7.0	45
Val-61	Fmoc-Val-OH[a]	217.2	7.0	45
Ala-60	Fmoc-Ala-OH[a]	199.3	7.0	45
Gln-59	Fmoc-Gln(Trt)-OH[a]	390.9	7.0	45
Gln-58	Fmoc-Gln(Trt)-OH[a]	390.9	7.0	45
Gly-57	Fmoc-(Dmb)Gly-OH[b]	171.8	7.0	90
Val-56	Fmoc-Val-OH[c]	434.4	14.0	90
Glu-55	Fmoc-Glu(OtBu)-OH[a]	272.3	7.0	45
Nle-54	Fmoc-Nle-OH[a]	226.2	7.0	45
Arg-53	Fmoc-Arg(Pbf)-OH[a]	415.2	7.0	45
Lys-52	Fmoc-Lys(Boc)-OH[a]	299.9	7.0	45
Trp-51	Fmoc-Trp(Boc)-OH[a]	337.0	7.0	45
Ala-50	Fmoc-Ala-OH[a]	199.3	7.0	45
Tyr-49	Fmoc-Tyr(tBu)-OH[a]	294.1	7.0	45
Phe-48	Fmoc-Phe-OH[a]	248.0	7.0	45
Asn-47	Fmoc-Asn(Trt)-OH[a]	381.9	7.0	45
Val-46	Fmoc-Val-OH[a]	217.2	7.0	45
Lys-45	Fmoc-Lys(Boc)-OH[a]	299.9	7.0	45
Thr-44, Asp-43	Fmoc-Asp(OtBu)-Thr($\psi^{Me,Me}$pro)-OH[b]	212.2	4.5 + 2.5 mL DMF	90
Pro-42	Fmoc-Pro-OH[a]	215.9	7.0	45
Val-41	Fmoc-Val-OH[a]	217.2	7.0	45
Thr-40, Ile-39	Fmoc-Ile-Thr($\psi^{Me,Me}$pro)-OH[b]	189.9	4.5 + 2.5 mL DMF	90
Asp-38	Fmoc-Asp(OPhiPr)-OH[b]	181.8	4.5 + 2.5 mL DMF	90
Glu-37	Fmoc-Glu(OtBu)-OH[a]	272.3	7.0	45
Asn-36	Fmoc-Asn(Trt)-OH[a]	381.9	7.0	45
Leu-35	Fmoc-Leu-OH[a]	226.2	7.0	45
Ser-34	Fmoc-Ser(tBu)-OH[a]	245.4	7.0	45
Cys-33	Fmoc-Cys(Acm)-OH[d]	530.5	-	210
His-32	Fmoc-His(Trt)-OH[a]	396.6	7.0	45
Glu-31	Fmoc-Glu(OtBu)-OH[a]	272.3	7.0	45
Ala-30	Fmoc-Ala-OH[a]	199.3	7.0	45
Cys-29	Boc-Cys(Acm)-OH[d]	374.2	-	210

[a] Activation of this amino acid (640.0 µmol, 5 equiv.) was performed with HCTU (254.2 mg, 614.4 µmol, 4.8 equiv.)

[b] Activation of this amino acid (384.0 µmol, 3 equiv.) was performed with PyBOP (193.2 mg, 371.2 µmol, 2.9 equiv.)

[c] Activation of this amino acid (1.28 mmol, 10 equiv.) was performed with HCTU (518.9 mg, 1.25 mmol, 9.8 equiv.)

[d] Activation and coupling of cysteine derivatives (1.28 mmol, 10 equiv.) was performed following the general procedure (anhydride formation with DIC (99.1 µL, 640.0 µmol, 5 equiv.) in 7.1 mL DMF/CH_2Cl_2 (1:2.5), coupling in 6.1 mL DMF).

Peptidyl resin **S2** (863.2 mg, loading = 91.9 µmol/g, 79.3 µmol) was treated with dilute HFIP according to the general procedure (yield: 478.9 mg crude peptidyl acid **S3**, 63.3 µmol, 79.8 %, TLC: CH_2Cl_2/MeOH 10:1; R_f (**S3**) = 0.40). Under argon, 300 mg of the cleaved peptidyl acid (39.6 µmol, 1 equiv.) were dissolved in absolute CH_2Cl_2 (3.3 mL, 12 mM **S3**) and BocHN-NH_2 (31.4 mg, 237. 8 µmol, 6 equiv.), Cl-HOBt (26.9 mg, 158.5 µmol, 4 equiv.) followed by addition of DIPEA (37.1 µL, 217.9 µmol, 5.5 equiv.) at 0 °C. DIC (24.5 µL, 158.5 µmol, 4 equiv.) was added and the mixture was kept at 0 °C until complete conversion (TLC, 10:1 CH_2Cl_2/MeOH). The reaction mixture was diluted with 150 mL of CH_2Cl_2 and extracted with 150 mL of 2 M $KHCO_3$. The aqueous phase was extracted twice with 150 mL of CH_2Cl_2. The organic phases were combined and dried over $MgSO_4$. The filtrate was adsorbed onto silica gel 60 (63 - 200 µm) and purified by flash chromatography (Reveleris iES, FlashPure cartridge 40 g (35 - 45 µm), 0-10 % MeOH in CH_2Cl_2 over 40 min, 40 mL/min). The fractions were analyzed by TLC, dried and lyophilized from dioxane.

Yield of **19**: 137.4 mg (17.9 µmol, 36.1 % over 3 steps). R_f (**19**) = 0.53 (CH_2Cl_2/MeOH 10:1). RP-HPLC-MS: column [C], 95 % MeCN/H_2O for 1.25 min, then 95-100 % MeCN/H_2O + 0.1 % HCOOH over 9 min, 0.5 mL/min, injection volume: 4 µL of 5 mg **19**/mL DMF. ESI-MS of **19**: *m/z* (average isotopes) $C_{414}H_{547}N_{57}O_{79}S_3$ (7682.43); calculated: 3853.21 $[M+H+Na]^{2+}$, 3842.22 $[M+2H]^{2+}$, 2561.82 $[M+3H]^{3+}$; found: 3853.92 $[M+H+Na]^{2+}$, 3842.83 $[M+2H]^{2+}$, 2563.06 $[M+3H]^{3+}$.

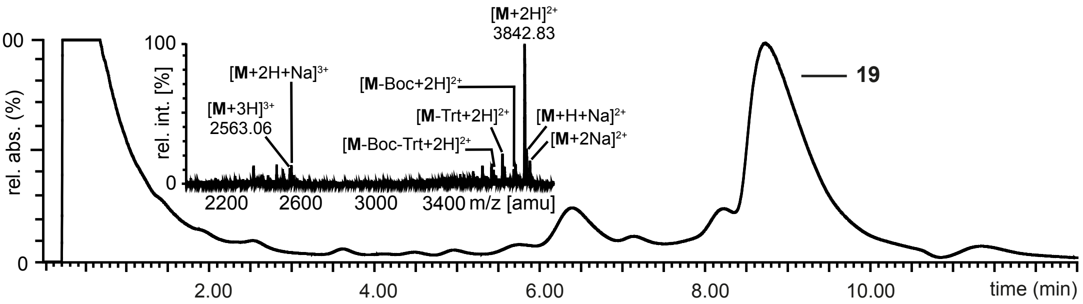

Figure S2: RP-HPLC-MS of protected EPO 29-67 hydrazide **19**.

Deprotection of **19** with TFA/TIS/H_2O (95:2.5:2.5): RP-HPLC-MS: column [A], 10-40 % MeCN/H_2O + 0.1 % HCOOH. ESI-MS of **19d** *m/z* (average isotopes) $C_{205}H_{315}N_{57}O_{62}S_2$ (4634.23); calculated: 2318.12 $[M+2H]^{2+}$, 1545.75 $[M+3H]^{3+}$, 1159.57 $[M+4H]^{4+}$; found: 2318.37 $[M+2H]^{2+}$, 1545.68 $[M+3H]^{3+}$, 1159.45 $[M+4H]^{4+}$.

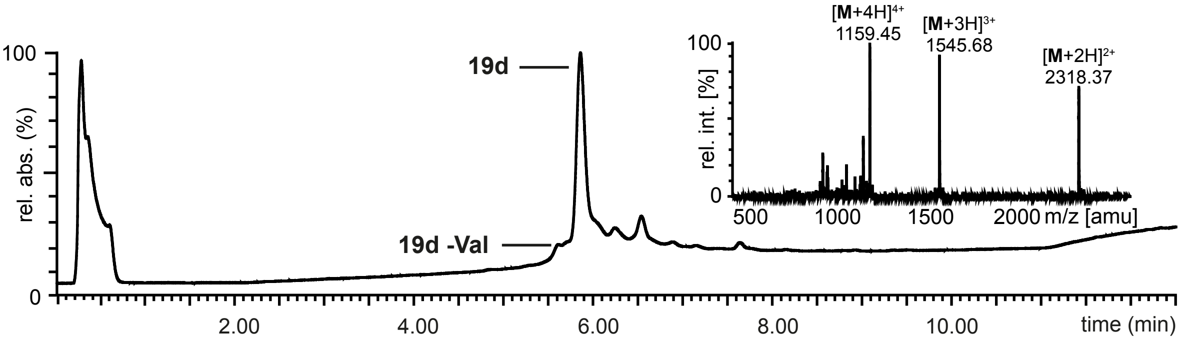

Figure S3: RP-HPLC-MS of deprotected EPO 29-67 hydrazide **19d**.

3.2 Selective Deprotection of EPO 29-67 Hydrazide 19

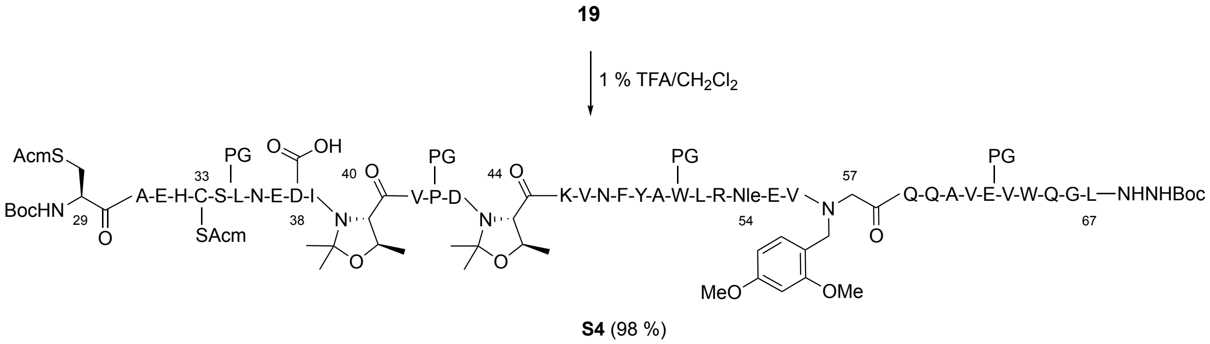

Figure S4: Synthesis of EPO 29-67 hydrazide **S4**

The peptide hydrazide **19** (130.1 mg, 16.9 µmol) was dissolved in 8.5 mL of 1 % TFA in CH_2Cl_2 (*c* **19** = 2 mM). After shaking for 10 min, 85 mL of cold Et_2O (-24 °C) were added and the suspesion was kept for 20 min in a cooling bath at –24 °C followed by centrifugation (4000 g, 4 °C, 10 min). The supernatant was decantated and the precipitate was resuspended in cold Et_2O. After centrifugation the hydrazide **S4** was dried *in vacuo* and subsequently lyophilized from dioxane. Yield of **S4**: 124.9 mg (16.5 µmol, 97.6 %). RP-HPLC-MS: column [C], 95 % for 1.25 min, then 95-100 % MeCN/H_2O + 0.1 % HCOOH in 9 min, 0.5 mL/min, injection volume 4 µL of 5 mg **S4**/mL DMF. ESI-MS of **S4**: *m/z* (average isotopes) $C_{405}H_{537}N_{57}O_{79}S_3$ (7564.25) calculated: 3783.13 $[M+2H]^{2+}$, 2522.42 $[M+3H]^{3+}$; found: 3782.80 $[M+2H]^{2+}$, 2522.02 $[M+3H]^{3+}$.

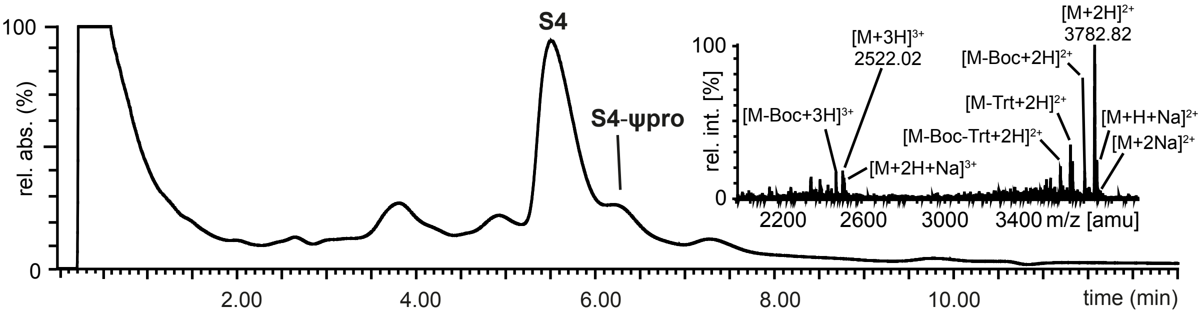

Figure S5: RP-HPLC-MS of protected EPO 29-67 hydrazide **S4**.

3.3 Synthesis of EPO 68-97 Hydrazide 22

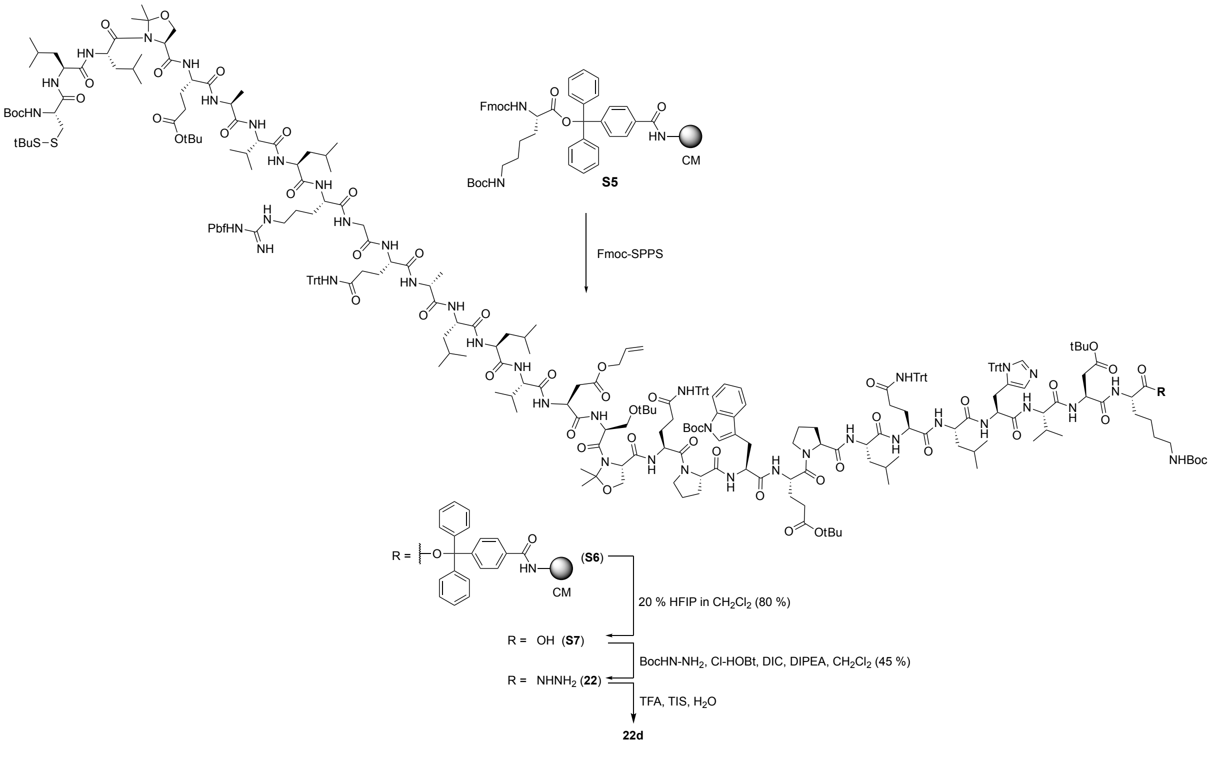

Figure S6: Synthesis of EPO 68-97 hydrazide **22**

Fmoc-Lys(Boc)-Trt-CM resin **S5** (loading = 264.3 µmol/g) was synthesized according to Ref.[8]. 462.8 mg (129.9 µmol, 1 eq) of resin **S5** were placed in a 45 mL peptide synthesizer reaction vessel and the peptide chain was elongated automatically. For cleavage of the Fmoc group the general procedure was applied and the amino acid building blocks were coupled under the conditions denoted in table S2 and the general procedure section. After complete elongation the resin **S6** was washed with DMF (5x) and CH_2Cl_2 (5x) and was dried *in vacuo*.

Table S2:

Position	Building Block	m (mg)	V (mL) 0.19 M DIPEA in DMF	Coupling duration (min)
Asp-96	Fmoc-Asp(OtBu)-OH[a]	267.4	6.5 + 0.5 mL DMF	45
Val-95	Fmoc-Val-OH[a]	220.6	6.5 + 0.5 mL DMF	45
His-94	Fmoc-His(Trt)-OH[a]	402.8	6.5 + 0.5 mL DMF	45
Leu-93	Fmoc-Leu-OH[a]	229.7	6.5 + 0.5 mL DMF	45
Gln-92	Fmoc-Gln(Trt)-OH[a]	397.0	6.5 + 0.5 mL DMF	45
Leu-91	Fmoc-Leu-OH[a]	229.7	6.5 + 0.5 mL DMF	45
Pro-90	Fmoc-Pro-OH[a]	219.3	6.5 + 0.5 mL DMF	45
Glu-89	Fmoc-Glu(OtBu)-OH[a]	276.6	6.5 + 0.5 mL DMF	45
Trp-88	Fmoc-Trp(Boc)-OH[a]	342.3	6.5 + 0.5 mL DMF	45
Pro-87	Fmoc-Pro-OH[a]	219.3	6.5 + 0.5 mL DMF	45
Gln-86	Fmoc-Gln(Trt)-OH[a]	397.0	6.5 + 0.5 mL DMF	45
Ser-85, Ser-84	Fmoc-Ser(tBu)-Ser($\psi^{Me,Me}$pro)-OH[b]	199.1	4.0 + 3.0 mL DMF	90
Asp-83	Fmoc-Asp(OAll)-OH[a]	257.0	6.5 + 0.5 mL DMF	45
Val-82	Fmoc-Val-OH[a]	220.6	6.5 + 0.5 mL DMF	45
Leu-81	Fmoc-Leu-OH[a]	229.7	6.5 + 0.5 mL DMF	45
Leu-80	Fmoc-Leu-OH[a]	229.7	6.5 + 0.5 mL DMF	45
Ala-79	Fmoc-Ala-OH[a]	202.4	6.5 + 0.5 mL DMF	45
Gln-78	Fmoc-Gln(Trt)-OH[a]	397.0	6.5 + 0.5 mL DMF	45
Gly-77	Fmoc-Gly-OH[a]	193.3	6.5 + 0.5 mL DMF	45
Arg-76	Fmoc-Arg(Pbf)-OH[a]	421.7	6.5 + 0.5 mL DMF	45
Leu-75	Fmoc-Leu-OH[a]	229.7	6.5 + 0.5 mL DMF	45
Val-74	Fmoc-Val-OH[a]	220.6	6.5 + 0.5 mL DMF	45
Ala-73	Fmoc-Ala-OH[a]	202.4	6.5 + 0.5 mL DMF	45
Glu-72	Fmoc-Glu(OtBu)-OH[a]	276.6	6.5 + 0.5 mL DMF	45
Ser-71, Leu-70	Fmoc-Leu-Ser($\psi^{Me,Me}$pro)-OH[b]	187.4	4.0 + 3.0 mL DMF	90
Leu-69	Fmoc-Leu-OH[a]	229.7	6.5 + 0.5 mL DMF	45
Cys-68	Boc-Cys(StBu)-OH[c]	402.3	-	180

[a] Activation of this amino acid (649.5 μmol, 5 equiv.) was performed with HCTU (258.1 mg, 623.5 μmol, 4.8 equiv.)

[b] Activation of this amino acid (389.7 μmol, 3 equiv.) was performed with PyBOP (196.2 mg, 376.7 μmol, 2.9 equiv.)

[c] Activation and coupling of cysteine derivatives (1.30 mmol, 10 equiv.) was performed following the general procedure (anhydride formation with DIC (100.7 μL, 649.5 μmol, 5 equiv.) in 7.2 mL DMF/CH_2Cl_2 (1:2.5), coupling in 3.8 mL DMF).

Then 483.7 mg of the peptidyl resin **S6** (loading = 116.1 μmol/g, 56.2 μmol) was treated with dilute HFIP according to the general procedure. Yield of peptidyl acid **S7**: 276.9 mg, 52.2 μmol,

92.9 %, R_f (**S7**) = 0.41 (CH_2Cl_2/MeOH 10:1).

The cleaved peptidyl acid **S7** (186.6 mg, 35.2 µmol, 1 equiv.) was dissolved in 1.9 mL of absolute CH_2Cl_2 followed by addition of BocHN-NH_2 (27.9 mg, 211.2 µmol, 6 equiv.), Cl-HOBt (23.9 mg, 140.8 µmol, 4 equiv.), and DIPEA (32.9 µL, 193.6 µmol, 5.5 equiv.) at 0 °C. DIC (21.8 µL, 140.8 µmol, 4 equiv.) was added and the mixture was kept at 0 °C until complete conversion (TLC, CH_2Cl_2/MeOH 10:1). The reaction mixture was diluted with 150 mL of CH_2Cl_2 and washed once with 150 mL of 2 M $KHCO_3$. The aqueous phase was extracted twice with 100 mL of CH_2Cl_2. The organic phases were combined and dried over $MgSO_4$. The filtrate was adsorbed onto silica gel 60 (63 - 200 µm) and purified by flash chromatography (Reveleris iES, FlashPure cartridge 40 g (35 - 45 µm), 0-10 % MeOH in CH_2Cl_2 over 40 min, 40 mL/min). The fractions were analyzed by TLC, dried and lyophilized from dioxane. Yield: 163.7 mg (30.2 µmol, 79.9 % over 3 steps). R_f (**22**) = 0.53 (CH_2Cl_2/MeOH 10:1). $C_{287}H_{400}N_{42}O_{55}S_3$ (5414.78).

Deprotection of **22** with TFA/TIS/H_2O (95:2.5:2.5): RP-HPLC-MS: column [A], 10-50 % MeCN/H_2O + 0.1 % HCOOH. ESI-MS of **22d** *m/z* (average isotopes) $C_{156}H_{256}N_{42}O_{44}S_2$ (3488.13) calculated: 3489.14 $[M+H]^+$, 1745.07 $[M+2H]^{2+}$, 1163.72 $[M+3H]^{3+}$, 873.04 $[M+4H]^{4+}$; found: 3488.98 $[M+H]^+$, 1744.47 $[M+2H]^{2+}$, 1163.32 $[M+3H]^{3+}$, 872.98 $[M+4H]^{4+}$.

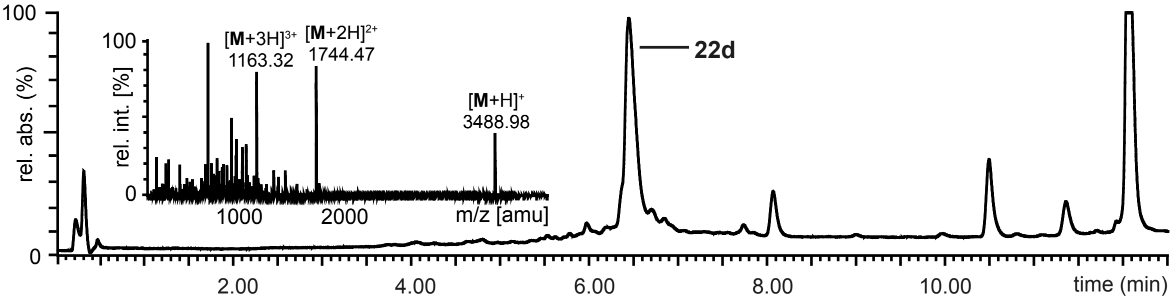

Figure S7: RP-HPLC-MS of deprotected EPO 68-97 hydrazide **22d**.

3.4 Selective Deprotection of EPO 68-97 Hydrazide 22

22

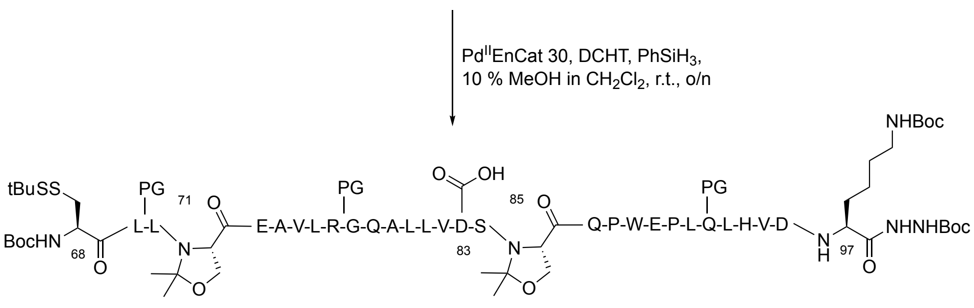

S8

Figure S8: Synthesis of deallylated EPO 68-97 hydrazide **S8**.

To a solution of peptide hydrazide **22** (82.8 mg, 15.3 µmol, 1 equiv.) in 510 µL of 10 % MeOH in CH_2Cl_2 were added phenylsilane (28.3 µL, 229.5 µmol, 15 eq), 2-[(dicyclohexylphosphino)ethyl]trimethyl-ammonium chloride (DCHT) (8.81 mg, 27.5 µmol, 1.8 eq) and Pd^{II}EnCat 30 (11.48 mg, loading: 0.4 mmol/g, 0.3 eq) under an argon atmosphere and exclusion of light. The suspension was stirred slowly until complete conversion of the allyl ester **22** (TLC: CH_2Cl_2/MeOH 15:1; R_f = 0.38 (**22**); 0.32 (**S8**)). The reaction mixture was filtered and the residue was washed with CH_2Cl_2. The filtrate was extracted twice with H_2O and the combined aqueous phases were extracted twice with CH_2Cl_2. The combined organic phases were dried over $MgSO_4$ and the filtrate was adsorbed to silica gel 60 (63 - 200 µm) and purified by flash chromatography (Reveleris iES, FlashPure cartridge 12 g (35 - 45 µm), 0-10 % MeOH in CH_2Cl_2 over 30 min, 28 mL/min). The fractions containing **S8** were combined and concentrated. The peptide hydrazide **S8** was taken up in 500 µL of CH_2Cl_2 and precipitated with cold Et_2O (10 mL) for 30 min at -24 °C. After centrifugation the supernatant was decanted and the residue was suspended in cold Et_2O (-24 °C) and centrifuged. The precipitate was dried *in vacuo* and lyophilized from dioxane. Yield: 60.7 mg (11.3 µmol, 73.9 %). R_f (**S8**) = 0.32 (CH_2Cl_2/MeOH 15:1). $C_{284}H_{396}N_{42}O_{55}S_3$ (5374.71).

Deprotection of **S8** with TFA/TIS/H_2O (95:2.5:2.5): RP-HPLC-MS: column [A], 10 % for 1.25 min, then 10-50 % MeCN/H_2O + 0.1 % HCOOH over 9 min, 0.5 mL/min. ESI-MS of **S8d** *m/z* (average isotopes) $C_{153}H_{252}N_{42}O_{44}S_2$ (3448.07) calculated: 3449.08 $[M+H]^+$, 1725.04 $[M+2H]^{2+}$, 1150.36 $[M+3H]^{3+}$, 863.02 $[M+4H]^{4+}$; found: 3445.75 $[M+H]^+$, 1722.80 $[M+2H]^{2+}$, 1148.86 $[M+3H]^{3+}$, 862.17 $[M+4H]^{4+}$.

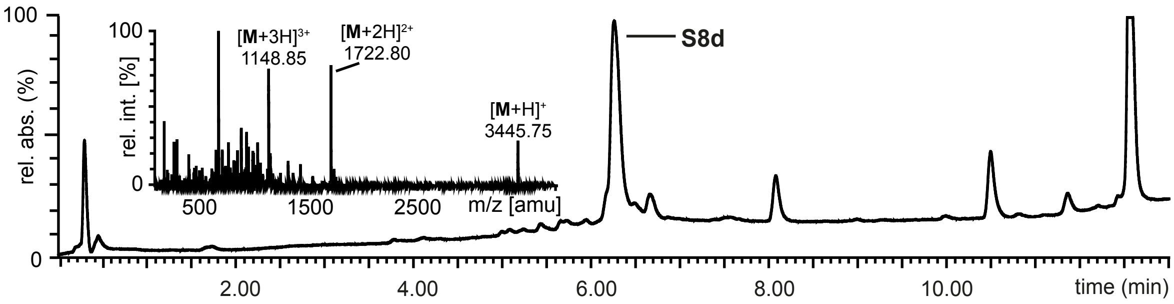

Figure S9: RP-HPLC-MS of deprotected EPO 68-97 hydrazide **S8d**.

3.5 Synthesis of EPO 29-67 Glycopeptide Hydrazide 20

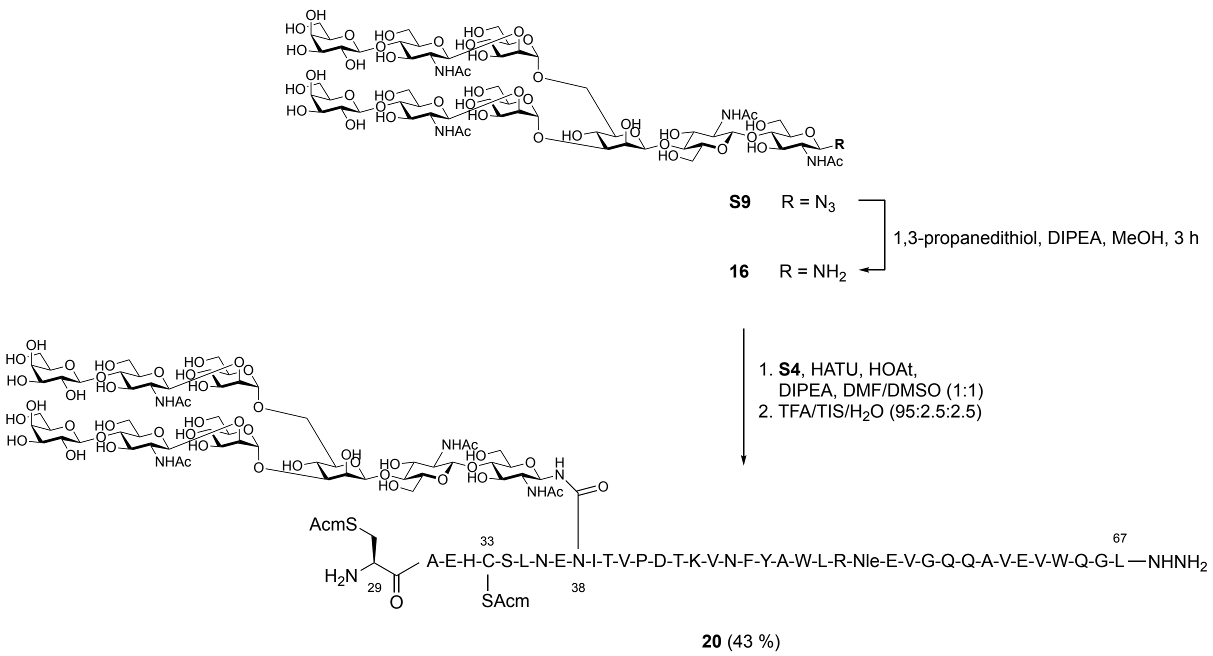

Figure S10: Synthesis of EPO 29-67 glycopeptide hydrazide **20**

Nonasaccharide azide **S9** [9] was taken up in 100 - 200 µL H_2O and lyophilized prior to reduction. Under an argon atmosphere the glycan azide **S9** (9.08 mg, 5.45 µmol, 1 equiv.) was dissolved in absolute MeOH (606 µL, 9 mM **S9**) followed by addition of DIPEA (abs., 18.54 µL, 109.0 µmol, 20 equiv.) and 1,3-propanedithiol (65.5 µL, 654.0 µmol, 120 equiv.). The solution was shaken at ambient temperature. After complete conversion (TLC: *i*PrOH/1 M NH_4OAc 2:1; R_f **S9** = 0.47; R_f **16** = 0.20), the solvent was removed *in vacuo*. The residue was suspended in 1.5 mL of cold Et_2O (-24 °C) and the glycosylamine was precipitated for 10 min at -24 °C in a cooling bath. After centrifugation (20 000g, 3 min), the supernatant was discarded

and the residue was triturated with cold Et_2O twice in an analogous manner. Finally, the glycosylamine **16** was dried *in vacuo* and used directly for aspartylation.

The aspartyl peptide **S4** (51.54 mg, 6.81 µmol, 1.25 equiv.) was dissolved in 151.6 µL of an activation mixture (5.18 mg of HATU (13.63 µmol, 2.5 equiv.), 1.85 mg of HOAt (13.63 µmol, 2.5 equiv.) and 7.42 µL of DIPEA (43.60 µmol, 8 equiv.) in DMF/DMSO (abs, 1:1)). After 10 min, the activated peptide was added to the glycosylamine **16** and the vial was rinsed three times with 63 µL each of DMF/DMSO (abs., 1:1). The reaction mixture was shaken at ambient temperature for 17 h. Subsequently, 19.5 mL of precooled TFA/TIS/H_2O (95:2.5:2.5) were added. The solution was shaken for 2.5 h, concentrated *in vacuo* to 2 mL followed by addition of cold Et_2O (30 mL, -24 °C) for 20 min in a cooling bath at -24 °C. After centrifugation (5000 g, 3 min), the supernatant was discarded. The residue was triturated again with cold Et_2O, dried *in vacuo*, taken up in 25 % MeCN/H_2O + 0.1 % TFA and lyophilized. The lyophilizate was taken up in 1.5 mL of 25 % MeCN/H_2O + 0.1 % TFA, and the glycopeptide hydrazide **20** was purified by RP-HPLC (column [F], 25-35 % MeCN/H_2O + 0.1 % TFA, 8 mL/min) and lyophilized. Yield of **20**: 14.51 mg (2.32 µmol, 42.6 %). RP-HPLC-MS: column [A], 10-30 % MeCN/H_2O + 0.1 % HCOOH. ESI-MS of **20** *m/z* (average isotopes) $C_{267}H_{418}N_{62}O_{106}S_2$ (6256.73) calculated: 3129.37 $[M+2H]^{2+}$, 2086.58 $[M+3H]^{3+}$, 1565.19 $[M+4H]^{4+}$; found: 3129.88 $[M+2H]^{2+}$, 2086.77 $[M+3H]^{3+}$, 1565.26 $[M+4H]^{4+}$.

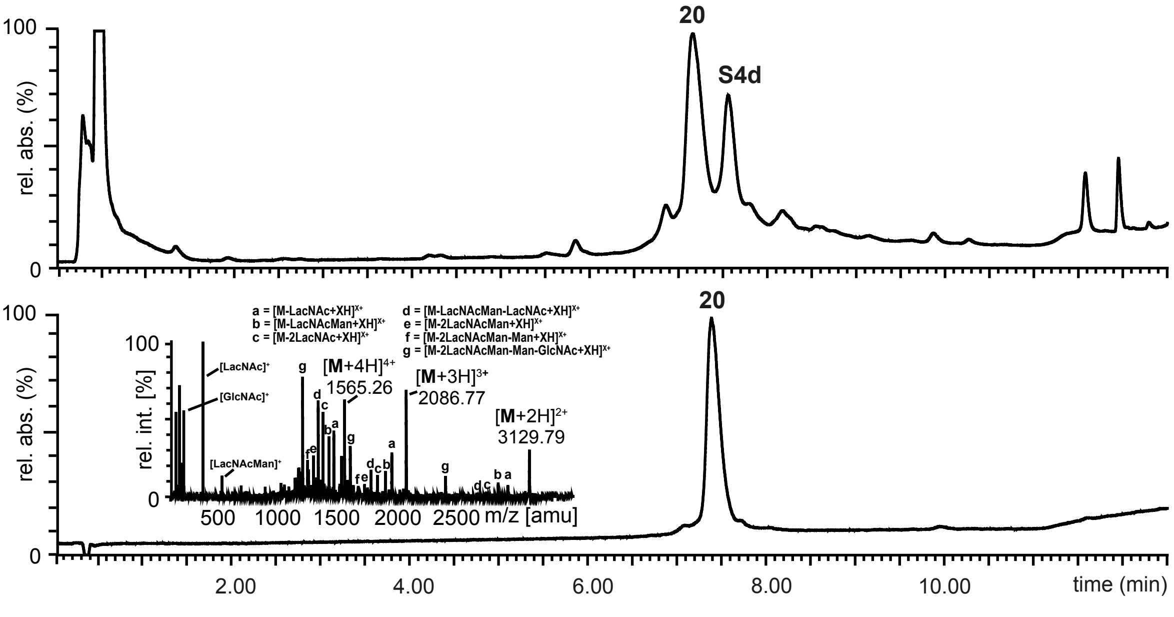

Figure S11: RP-HPLC-MS of EPO 29-67 glycopeptide hydrazide **20** a) crude and b) after purification by RP-HPLC.

3.6 Synthesis of EPO 68-97 Glycopeptide Hydrazide 23

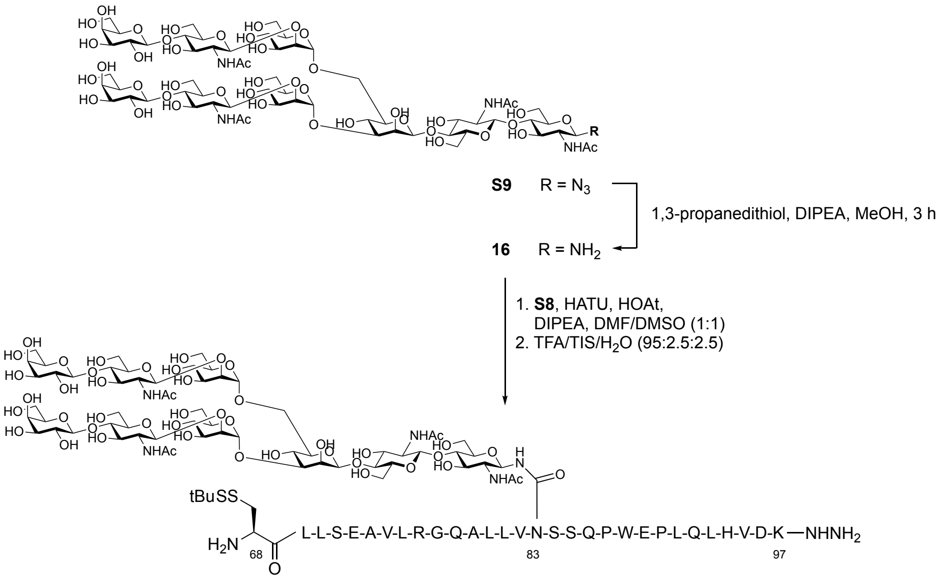

Figure S12: Synthesis of EPO 68-97 glycopeptide hydrazide **23**

Nonasaccharide azide **S9** [9] was taken up in 100 - 200 µL H_2O and lyophilized prior to reduction. Under an argon atmosphere the glycan azide **S9** (9.23 mg, 5.54 µmol, 1 equiv.) was dissolved in absolute MeOH (615 µL, 9 mM **S9**) followed by addition of DIPEA (abs., 18.84 µL, 110.8 µmol, 20 equiv.) and 1,3-propanedithiol (66.6 µL, 664.7 µmol, 120 equiv.). The solution was shaken at ambient temperature. After complete conversion (TLC: *i*PrOH/1 M NH_4OAc 2:1; R_f **S9** = 0.47; R_f **16** = 0.20), the solvent was removed *in vacuo*. The residue was suspended in 1.5 mL of cold Et_2O (-24 °C) and the glycosylamine was precipitated for 10 min at -24 °C in a cooling bath. After centrifugation (20 000 g, 3 min), the supernatant was discarded and the residue was triturated with cold Et_2O twice in in an analogous manner. Finally, the glycosylamine **16** was dried *in vacuo* and used directly for aspartylation.

The aspartyl peptide **S8** (37.21 mg, 6.92 µmol, 1.25 equiv.) was dissolved in 153.9 µL of an activation mixture (5.27 mg of HATU (13.85 µmol, 2.5 equiv.), 1.89 mg of HOAt (13.85 µmol, 2.5 equiv.) and 4.71 µL of DIPEA (27.70 µmol, 5 equiv.) in DMF/DMSO (abs, 1:1)). After 10 min, the activated peptide was added to the glycosylamine **16** and the vial was rinsed three times with 25.6 µL each of DMF/DMSO (abs., 1:1). The reaction mixture was shaken at ambient temperature for 17 h. Subsequently, 19.8 mL of precooled TFA/TIS/H_2O (95:2.5:2.5) were added to the aspartylation solution. The solution was shaken for 2.5 h, concentrated *in vacuo* to 2 mL followed by addition of cold Et_2O (30 mL, -24 °C) for 20 min in a cooling bath at -24 °C. After centrifugation (5000 g, 3 min), the supernatant was discarded. The residue was

triturated with cold Et_2O, dried *in vacuo*, taken up in taken up in 25 % $MeCN/H_2O$ + 0.1 % TFA and lyophilized. The lyophilizate was taken up in 3.0 mL of 25 % $MeCN/H_2O$ + 0.1 % TFA, and the glycopeptide hydrazide **23** was purified by RP-HPLC (column [F], 25-40 % $MeCN/H_2O$ + 0.1 % TFA, 8 mL/min) and lyophilized. Yield of **23**: 10.38 mg (2.05 µmol, 37.0 %). RP-HPLC-MS: column [A], 10-50 % $MeCN/H_2O$ + 0.1 % HCOOH. ESI-MS of **23** *m/z* (average isotopes) $C_{215}H_{355}N_{47}O_{88}S_2$ (5070.57 g/mol) calculated: 2536.29 $[M+2H]^{2+}$, 1691.20 $[M+3H]^{3+}$, 1268.65 $[M+4H]^{4+}$; found: 2534.65 $[M+2H]^{2+}$, 1689.85 $[M+3H]^{3+}$, 1267.92 $[M+4H]^{4+}$.

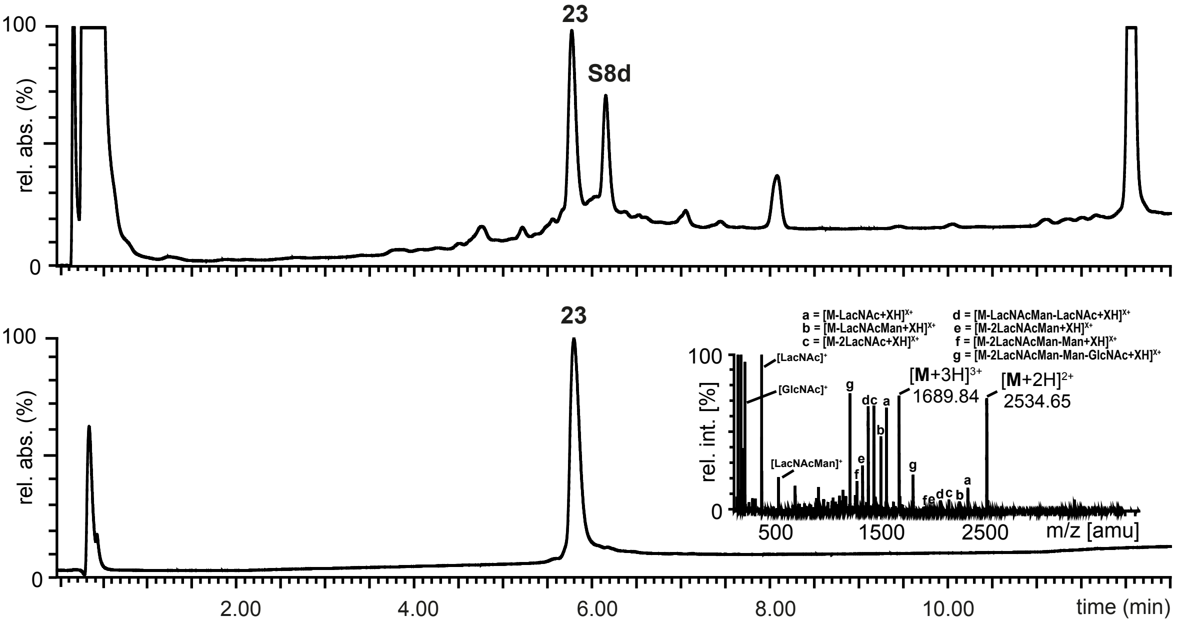

Figure S13: RP-HPLC-MS of EPO 68-97 glycopeptide hydrazide **23** a) crude and b) after purification by RP-HPLC.

4. Synthesis of the C-terminal Fragments

4.1 Synthesis of EPO 98-127 Thioester 4

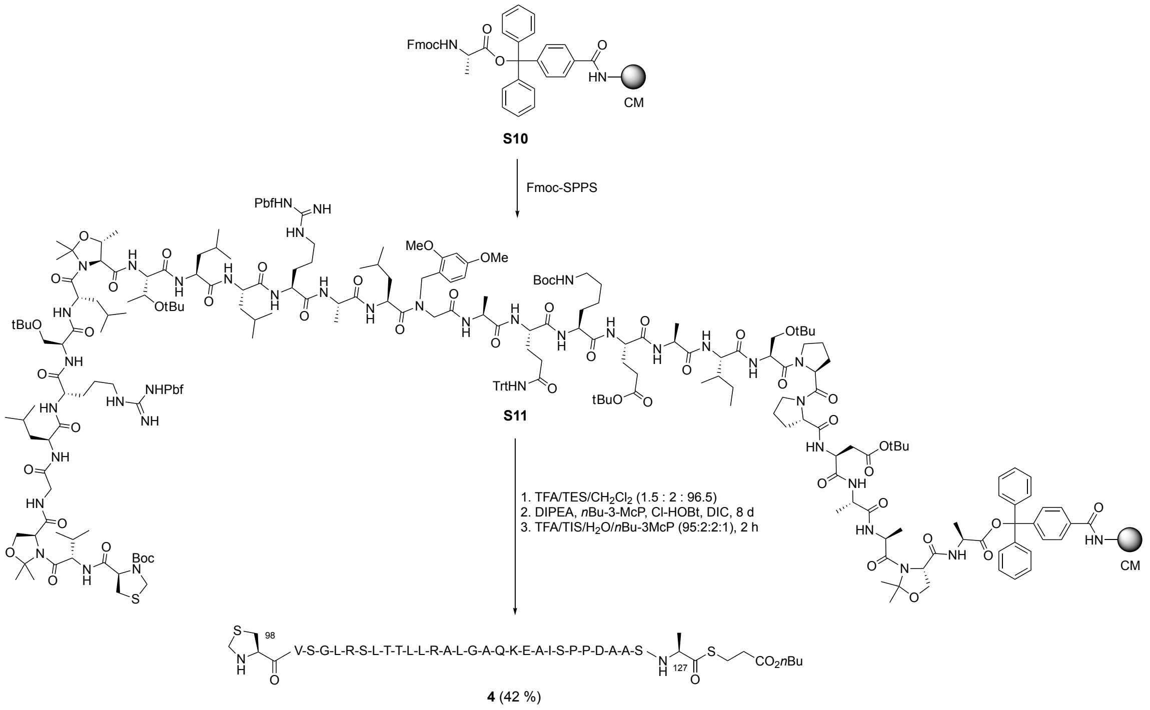

Figure S14: Synthesis of EPO 98-127 thioester **4**.

Fmoc-Ala-Trt-CM resin **S10** (loading = 370 µmol/g) was synthesized according to Ref.[8]. 351.4 mg (130 µmol, 1 eq) of resin **S10** were placed in a 45 mL peptide synthesizer reaction vessel and the peptide chain was elongated automatically. For cleavage of the Fmoc group the general procedure was applied and the amino acid building blocks were coupled under the conditions listed in table S3 and the general procedure section. After complete elongation the resin **S11** was washed with DMF (5x) and CH_2Cl_2 (5x) and was dried *in vacuo*.

Table S3:

Position	Building Block	*m* (mg)	*V* (mL) 0.4 M DIPEA in DMF	Coupling duration (min)
Ser-126, Ala-125	Fmoc-Ala-Ser(ψ^{Me,Me}pro)-OH[b]	144.7	1.5 + 1.5 mL DMF	60
Ala-124	Fmoc-Ala-OH[a]	171.2	3.0	45
Asp-123	Fmoc-Asp(OtBu)-OH[a]	226.3	3.0	45
Pro-122	Fmoc-Pro-OH[a]	185.6	3.0	45
Pro-121	Fmoc-Pro-OH[a]	185.6	3.0	45
Ser-120	Fmoc-Ser(tBu)-OH[a]	210.9	3.0	45

Ile-119	Fmoc-Ile-OH[a]	194.4	3.0	45
Ala-118	Fmoc-Ala-OH[a]	171.2	3.0	45
Glu-117	Fmoc-Glu(OtBu)-OH[a]	234.0	3.0	45
Lys-116	Fmoc-Lys(Boc)-OH[a]	257.7	3.0	45
Gln-115	Fmoc-Gln(Dmcp)-OH[b]	148.7	1.5 + 1.5 mL DMF	60
Ala-114	Fmoc-Ala-OH[a]	171.2	3.0	45
Gly-113	Fmoc-(Dmb)Gly-OH[b]	147.7	1.5 + 1.5 mL DMF	60
Leu-112	Fmoc-Leu-OH[c]	388.7	5.5	90
Ala-111	Fmoc-Ala-OH[a]	171.2	3.0	45
Arg-110	Fmoc-Arg(Pbf)-OH[a]	356.8	3.0	45
Leu-109	Fmoc-Leu-OH[a]	194.4	3.0	45
Leu-108	Fmoc-Leu-OH[a]	194.4	3.0	45
Thr-107	Fmoc-Thr(tBu)-OH[a]	218.6	3.0	45
Thr-106, Leu-105	Fmoc-Leu-Thr($\psi^{Me,Me}$pro)-OH[b]	421.7	1.5 + 1.5 mL DMF	60
Ser-104	Fmoc-Ser(tBu)-OH[a]	210.9	3.0	45
Arg-103	Fmoc-Arg(Pbf)-OH[a]	356.8	3.0	45
Leu-102	Fmoc-Leu-OH[a]	194.4	3.0	45
Gly-101	Fmoc-Gly-OH[a]	163.5	3.0	45
Ser-100, Val-99	Fmoc-Val-Ser($\psi^{Me,Me}$pro)-OH[b]	153.9	1.5 + 1.5 mL DMF	60
Cys-98	Boc-Thz-OH[b]	77.0	1.5 + 1.5 mL DMF	60

[a] Activation of this amino acid (550 µmol, 5 equiv.) was performed with HCTU (227.5 mg, 550 µmol, 5 equiv.)
[b] Activation of this amino acid (330 µmol, 3 equiv.) was performed with PyBOP (171.7 mg, 330 µmol, 3 equiv.)
[c] Activation of this amino acid (1.10 mmol, 10 equiv.) was performed with HCTU (455.1 mg, 1.10 mmol, 10 equiv.)

Peptidyl resin **S11** (247.6 mg, loading = 110 µmol/g, 27.2 µmol, 1 eq) was swelled with 5 mL of CH_2Cl_2 for 5 min and then treated with 7 mL of 1.5 % TFA in CH_2Cl_2 containing 2 % Et_3SiH (10 x, 2 min each). Subsequently, the resin was washed with CH_2Cl_2 (6 x, 7 mL each). All solutions were combined in a flask containing 2.0 mL of DIPEA in 20 mL of CH_2Cl_2 (11.84 mmol, 1.1 eq based on TFA). Further 2.0 mL of DIPEA were added and the solution was concentrated *in vacuo* to a final volume of 6 mL. *n*-Butyl 3-mercaptopropanoate (132.7 mg, 817.2 µmol, 30 eq), Cl-HOBt (92.4 mg, 544.8 µmol, 20 eq) and DIC (84.9 µL, 544.8 µmol, 20 eq) were added. After 8 d 25 mL of TFA/Et_3SiH/H_2O/*n*Bu-3-McP (95:2:2:1) were added. After 2 h, the reaction mixture was concentrated *in vacuo* to 4 mL and precipitated with cold Et_2O (40 mL) in a cooling bath at –24 °C for 24 h. After centrifugation (5000 g, 3 min), the supernatant was discarded. The residue was triturated twice with Et_2O (40 mL each, –24 °C, 30 min), dried *in vacuo* and lyophilized from 50 % MeCN/H_2O + 0.1 % HCOOH. The crude product was dissolved in 6 mL of 50 % MeCN/H_2O + 0.1 % HCOOH and **4** was isolated by

two consecutive RP-HPLC purifications (1. Column [F], 20-50 % MeCN/H_2O + 0.1 % TFA, 8 mL/min; 2. Column [L], 20-50 % MeCN/H_2O + 0.1 % TFA, 4 mL/min) and was lyophilized. Yield of **4**: 36.2 mg (11.48 µmol, 42.2 %). RP-HPLC-MS: column [A], 20-60 % MeCN/H_2O + 0.1 % HCOOH. ESI-MS of **4**: *m/z* (average isotopes) $C_{136}H_{234}N_{38}O_{43}S_2$ (M = 3153.71) calculated: 3154.71 $[M+H]^+$, 1577.86 $[M+2H]^{2+}$, 1052.24 $[M+3H]^{3}$; found: 3154.79 $[M+H]^+$, 1577.39 $[M+2H]^{2+}$, 1051.91 $[M+3H]^{3+}$.

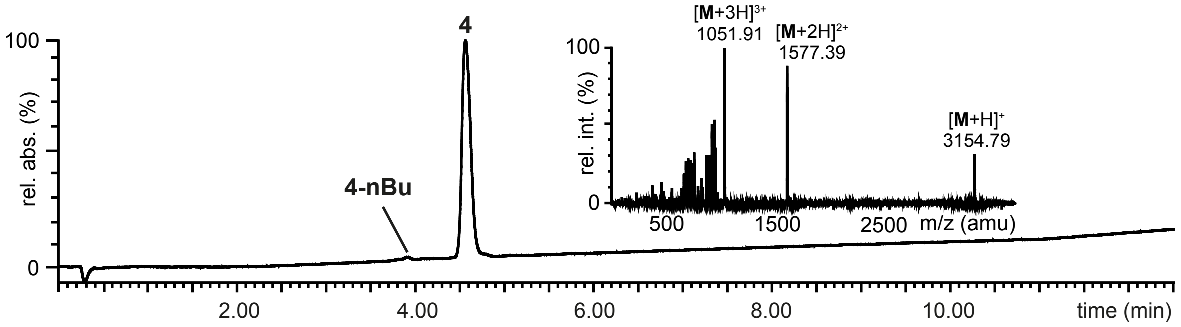

Figure S15: RP-HPLC-MS of purified EPO 98-127 Thioester **4**.

4.2 Synthesis of EPO 128-166 Peptidyl Acid 5

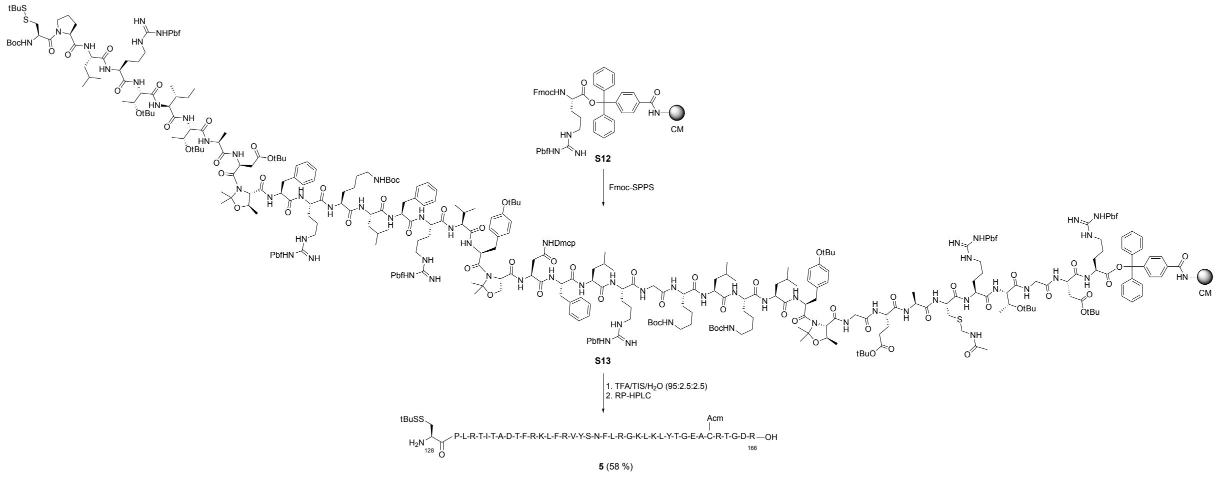

Figure S16: Synthesis of EPO 128-166 **5**.

Fmoc-Arg(Pbf)-Trt-CM resin **S12** (loading = 250 µmol/g) was synthesized according to Ref.[8]. 300.0 mg (75 µmol, 1 eq) of resin **S12** were placed in a 45 mL peptide synthesizer reaction vessel and the peptide chain was elongated automatically. For cleavage of the Fmoc group the general procedure was applied and the amino acid building blocks were coupled under the conditions denoted in table S4 and the general procedure section. After complete elongation the resin S**13** was washed with DMF (5 x) and CH_2Cl_2 (5 x) and was dried *in vacuo* (yield: 533.4 mg, loading = 50.3 µmol, 67.1 %).

Table S4:

Position	Building Block	*m* (mg)	*V* (mL) 0.4 M DIPEA in DMF	Coupling duration (min)
Asp-165	Fmoc-Asp(OtBu)-OH[a]	154.3	2.0	45
Gly-164	Fmoc-Gly-OH[a]	111.5	2.0	45
Thr-163	Fmoc-Thr(tBu)-OH[a]	149.1	2.0	45
Arg-162	Fmoc-Arg(Pbf)-OH[a]	243.3	2.0	45
Cys-161	Fmoc-Cys(Acm)-OH[c]	310.7	-	180
Ala-160	Fmoc-Ala-OH[a]	116.7	2.0	45
Glu-159	Fmoc-Glu(OtBu)-OH[a]	159.6	2.0	45
Gly-158	Fmoc-Gly-OH[a]	111.5	2.0	45
Thr-157, Tyr-156	Fmoc-Tyr(tBu)-Thr($\psi^{Me,Me}$pro)-OH[b]	225.3	2.0	45
Leu-155	Fmoc-Leu-OH[a]	132.5	2.0	45
Lys-154	Fmoc-Lys(Boc)-OH[a]	175.7	2.0	45
Leu-153	Fmoc-Leu-OH[a]	132.5	2.0	45
Lys-152	Fmoc-Lys(Boc)-OH[a]	175.7	2.0	45
Gly-151	Fmoc-Gly-OH[a]	111.5	2.0	45
Arg-150	Fmoc-Arg(Pbf)-OH[a]	243.3	2.0	45
Leu-149	Fmoc-Leu-OH[a]	132.5	2.0	45
Phe-148	Fmoc-Phe-OH[a]	145.3	2.0	45
Asn-147	Fmoc-Asn(Trt)-OH[a]	223.8	2.0	45
Ser-146, Tyr-145	Fmoc-Tyr(tBu)-Ser($\psi^{Me,Me}$pro)-OH[b]	220.0	2.0	45
Val-144	Fmoc-Val-OH[a]	127.3	2.0	45
Arg-143	Fmoc-Arg(Pbf)-OH[a]	243.3	2.0	45
Phe-142	Fmoc-Phe-OH[a]	145.3	2.0	45
Leu-141	Fmoc-Leu-OH[a]	132.5	2.0	45
Lys-140	Fmoc-Lys(Boc)-OH[a]	175.7	2.0	45
Arg-139	Fmoc-Arg(Pbf)-OH[a]	243.3	2.0	45
Phe-138	Fmoc-Phe-OH[a]	145.3	2.0	45
Thr-137, Asp-136	Fmoc-Asp(OtBu)-Thr($\psi^{Me,Me}$pro)-OH[b]	207.1	2.0	45
Ala-135	Fmoc-Ala-OH[a]	116.7	2.0	45
Thr-134	Fmoc-Thr(tBu)-OH[a]	149.1	2.0	45
Ile-133	Fmoc-Ile-OH[a]	132.5	2.0	45
Thr-132	Fmoc-Thr(tBu)-OH[a]	149.1	2.0	45
Arg-131	Fmoc-Arg(Pbf)-OH[a]	243.3	2.0	45
Leu-130	Fmoc-Leu-OH[a]	132.5	2.0	45
Pro-129	Fmoc-Pro-OH[a]	126.5	2.0	45
Cys-128	Boc-Cys(StBu)-OH[c]	232.1	-	180

[a] Activation of this amino acid (374.9 µmol, 5 equiv.) was performed with HCTU (155.1 mg, 374.9 µmol, 5 equiv.)

[b] Activation of this amino acid (374.9 µmol, 5 equiv.) was performed with PyBOP (195.1 mg, 374.9 µmol, 5 equiv.)

[c] Activation and coupling of cysteine derivatives (749.8 µmol, 10 equiv.) was performed following the general procedure (anhydride formation with DIC (58.4 µL, 374.9 µmol, 5 equiv.) in 4.2 mL DMF/CH_2Cl_2 (1:2.5), coupling in 3.6 mL DMF).

A portion of peptidyl resin **S13** (364.8 mg, loading = 94.3 µmol/g, 34.4 µmol) was suspended in 4.3 mL of TFA/TIS/H_2O (95:2.5:2.5) and shaken for 2.5 h. The cleavage solution was drained and the resin was washed three times for 10 min with TFA/TIS/H_2O (95:2.5:2.5, 1.5 mL each). The filtrates were combined, concentrated *in vacuo* to 2.0 mL and precipitated with Et_2O (20 mL) in a cooling bath at –24 °C for 20 min, followed by centrifugation (5000 g, 3 min). The precipitate was treated analogously with Et_2O (20 mL each) twice. The residue was dried *in vacuo*, taken up in 25 % MeCN/H_2O + 0.1 % TFA, filtered through a PTFE filter (0.2 µm) and lyophilized. The crude product (162.8 mg) was dissolved in 16.2 mL of 20 % MeCN/H_2O + 0.1 % TFA and purified by RP-HPLC (column [F], 20-50 % MeCN/H_2O + 0.1 % TFA, 8 mL/min) and lyophilized.

Yield of **5**: 94.3 mg (19.9 µmol, 57.8 %). RP-HPLC-MS: column [A], 10-50 % MeCN/H_2O + 0.1 % HCOOH. ESI-MS of **5**: *m/z* (average isotopes) $C_{210}H_{342}N_{62}O_{56}S_3$ (M = 4727.60 g/mol, M_{mi} = 4724.4982 Da) calculated: 946.53 $[M+5H]^{5+}$, 788.94 $[M+6H]^{6+}$, 676.38 $[M+7H]^{7+}$; found: 2364.55 946.29 $[M+5H]^{5+}$, 788.64 $[M+6H]^{6+}$, 676.08 $[M+7H]^{7+}$.

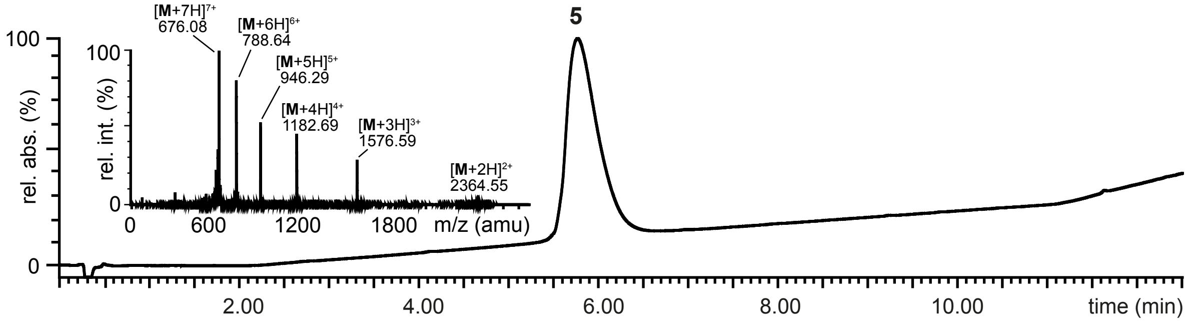

Figure S17: RP-HPLC-MS of purified EPO 128-166 **5**.

5. Synthesis of EPO A by Sequential NCL

5.1 Synthesis of EPO 29-67 Glycopeptide Thioester 21

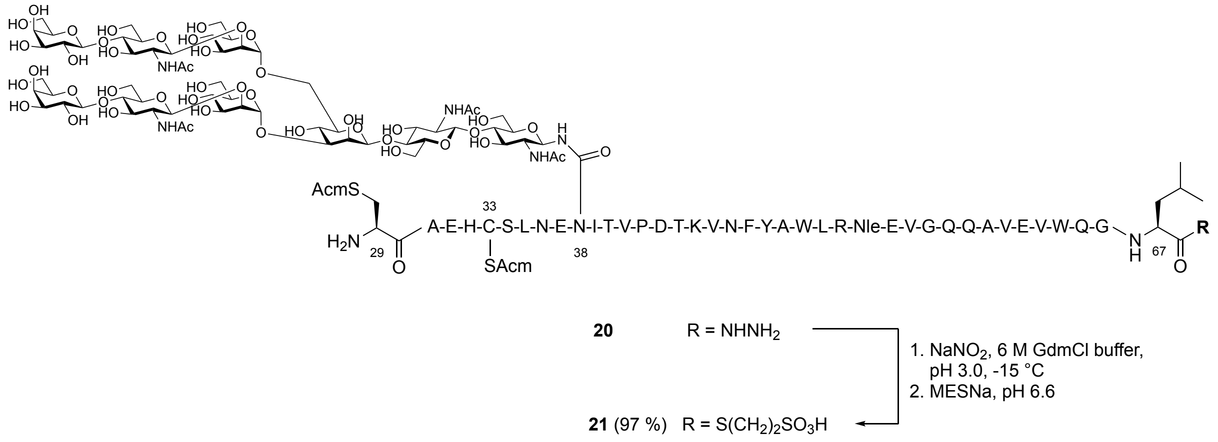

Figure S18: Synthesis of EPO 29-67 glycopeptide thioester **21**.

According to the general procedure for thioester formation, the glycopeptide hydrazide **20** (4.00 mg, 639.3 nmol, 1 equiv.) was dissolved in 182.7 µL of 6 M GdmCl buffer and reacted with 8.3 µL of 0.5 M $NaNO_2$ (4.16 µmol, 6.5 equiv.). After 1 h, 41.6 µL of the MESNa stock solution (41.56 µmol, 65 equiv.) were added. The pH value was adjusted to pH 6.6 and the reaction mixture was desalted after 1 h by gel filtration (column [O], 10 % MeCN/H_2O + 0.1 % TFA, 0.45 mL/min). The target fraction was lyophilized.

Yield of **21**: 3.94 mg (618.8 nmol, 96.8 %). RP-HPLC-MS: column [A], 10-30 % MeCN/H_2O + 0.1 % HCOOH. ESI-MS of **21**: *m/z* (average isotopes) $C_{269}H_{420}N_{60}O_{109}S_4$ (6366.87) calculated: 3184.44 $[M+2H]^{2+}$, 2123.30 $[M+3H]^{3+}$, 1592.72 $[M+4H]^{4+}$, 1274.38 $[M+5H]^{5+}$; found: 3182.67 $[M+2H]^{2+}$, 2122.02 $[M+3H]^{3+}$, 1591.74 $[M+4H]^{4+}$, 1273.69 $[M+5H]^{5+}$.

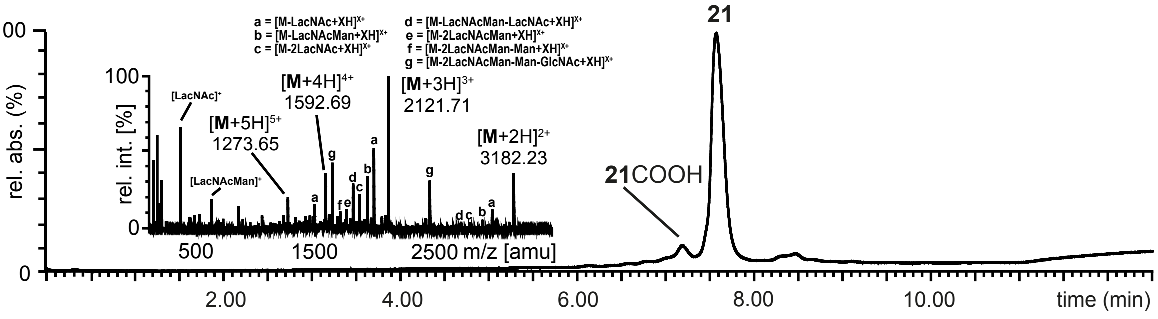

Figure S19: RP-HPLC-MS of EPO 29-67 glycopeptide thioester **21**.

5.2 Synthesis of EPO 29-97 Glycopeptide Hydrazide 25

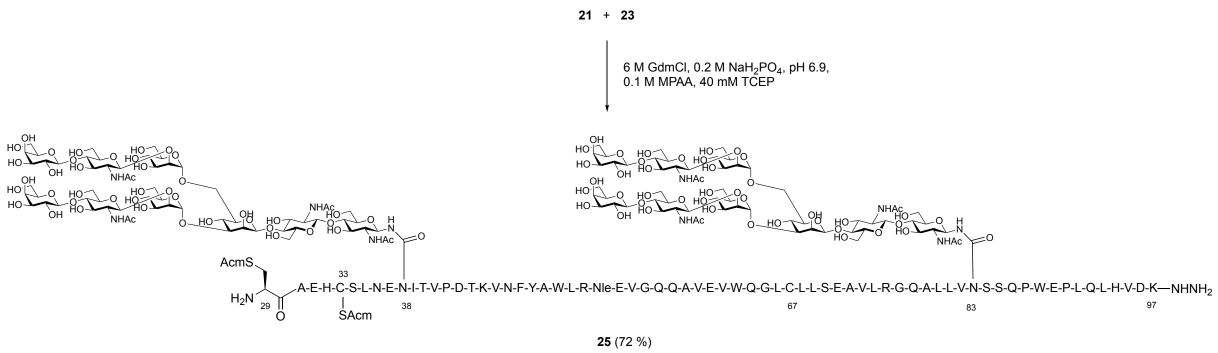

Figure S20: Synthesis of EPO 29-97 hydrazide **25**

The ligation buffer (6 M GdmCl, 0.2 M NaH_2PO_4, 40 mM TCEP, 0.1 M MPAA) was mixed under anaerobic conditions and adjusted to pH = 6.9. Glycopeptide hydrazide **23** (6.29 mg, 1.24 µmol, 1 equiv.) was dissolved in 620 µL of ligation buffer and added to the thioester **21** (7. 89 mg, 1.24 µmol, 1 equiv.). The mixture was incubated under anaerobic conditions until complete conversion of the thioester (28 h, LC-MS control, LC-MS samples were prepared with 20 mM TCEP in water). Prior to purification, the mixture was treated with TCEP x HCl (7.11 mg, 24.8 µmol, 20 equiv.) for 30 min. The glycopeptide hydrazide **25** was purified by RP-HPLC (column [G], 20-60 % MeCN/H_2O + 0.1 % TFA 3 mL/min).

Yield of **25**: 10.01 mg (893.2 nmol, 72.0 %) $C_{478}H_{761}N_{107}O_{194}S_3$ (11207.08). RP-HPLC: column [A], 20-60 % MeCN/H_2O + 0.1 % HCOOH. HR-MS of **25**: *m/z* (exact mass) $C_{478}H_{761}N_{107}O_{194}S_3$ (11200.2134) calculated: 1601.0378 $[M+7H]^{7+}$, 1401.0340 $[M+8H]^{8+}$, 1245.4754 $[M+9H]^{9+}$, 1121.0286 $[M+10H]^{10+}$, found: 1601.0373 $[M+7H]^{7+}$, 1401.0343 $[M+8H]^{8+}$, 1245.4735 $[M+9H]^{9+}$, 1121.0275 $[M+10H]^{10+}$.

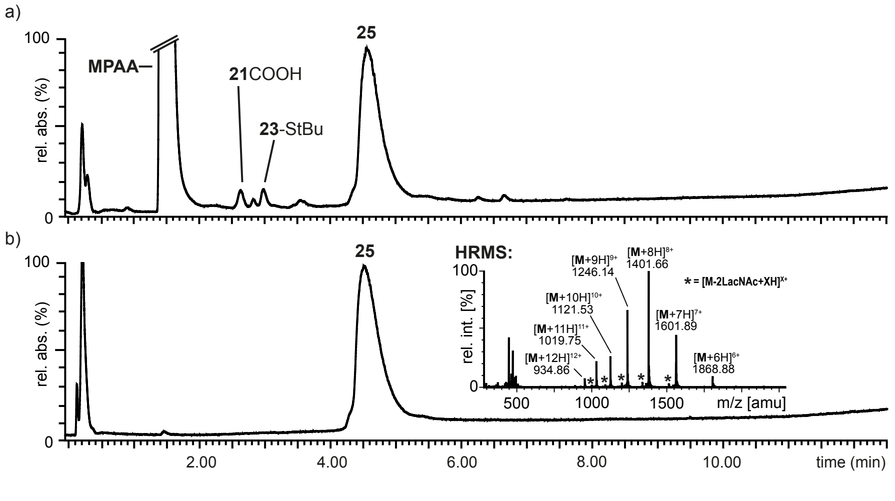

Figure S21: RP-HPLC-MS of EPO 29-97 glycopeptide **25** a) crude after 28 h and b) after RP-HPLC.

5.3 Synthesis of EPO 29-97 Glycopeptide Thioester S14

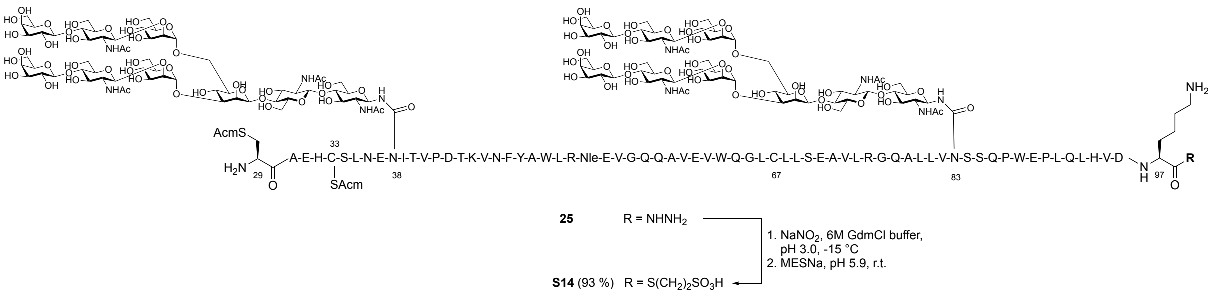

Figure S22: Synthesis of EPO 29-97 thioester **S14**

According to the general procedure for thioester formation, the glycopeptide hydrazide **25** (10.01 mg, 893.2 nmol, 1 equiv.) was dissolved in 255.2 µL of 6 M GdmCl buffer and reacted with 11.62 µL of 0.5 M $NaNO_2$ (5.81 µmol, 6.5 equiv.). After 1 h, 58.1 µL of the MESNa stock solution (58.06 µmol, 65 equiv.) were added. The pH was adjusted to a value of 6 and the reaction mixture was desalted after 1 h by gel filtration (column [M], 10 % MeCN/H_2O + 0.1 % TFA, 0.5 mL/min). The fraction containing **S14** was lyophilized.

Yield of **S14**: 9.42 mg (832.4 nmol, 93.2 %) $C_{480}H_{763}N_{105}O_{197}S_5$ (11317.22). RP-HPLC-MS: column [A], 20-60 % MeCN/H_2O + 0.1 % HCOOH.

HR-MS of **S14**: *m/z* (exact mass) $C_{480}H_{763}N_{105}O_{197}S_5$ (11310.1518 Da) calculated: 1886.0326 $[M+6H]^{6+}$, 1616.7432 $[M+7H]^{7+}$, 1414.7763 $[M+8H]^{8+}$, found: 1886.0195 $[M+6H]^{6+}$, 1616.7414 $[M+7H]^{7+}$, 1414.7638 $[M+8H]^{8+}$.

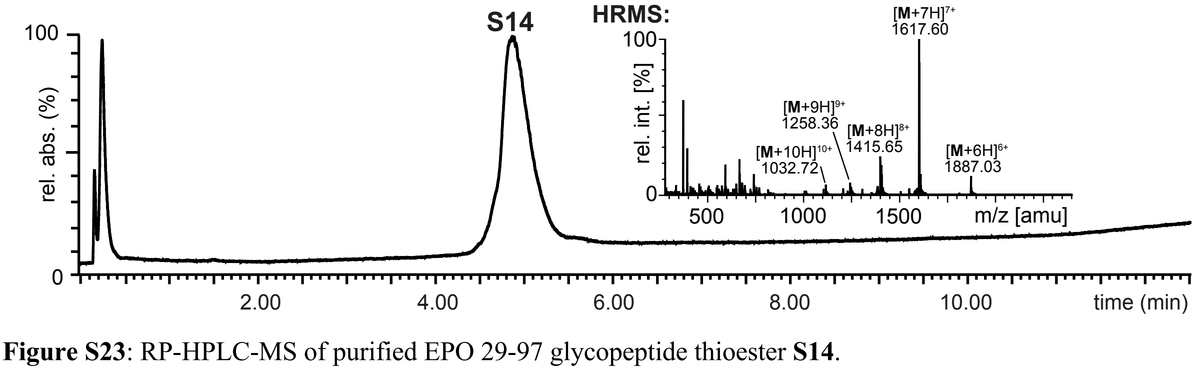

Figure S23: RP-HPLC-MS of purified EPO 29-97 glycopeptide thioester **S14**.

5.4 Synthesis of EPO 98-166 26

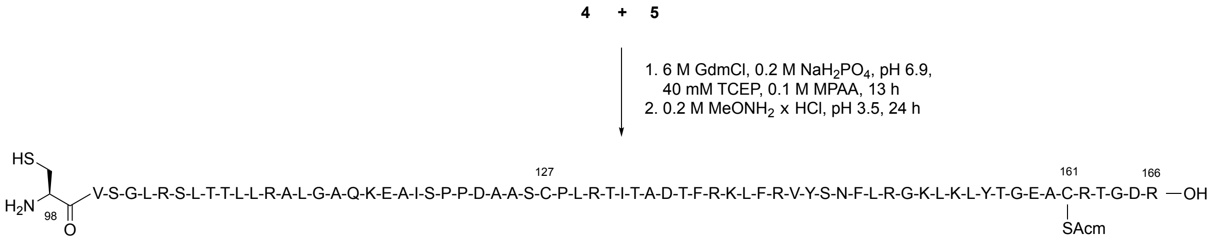

26 (89 %)

Figure S24: Synthesis of EPO 98-166 **26** by NCL.

The ligation buffer (6 M GdmCl, 0.2 M NaH_2PO_4, 40 mM TCEP, 0.1 M MPAA) was mixed under anaerobic conditions and adjusted to pH = 6.9. Peptide **5** (15.00 mg, 3.17 µmol, 1.2 equiv.) was dissolved in 1.59 mL of ligation buffer and added to the thioester **4** (8. 34 mg, 2.64 µmol, 1 equiv.). The mixture was incubated under anaerobic conditions until complete conversion of the thioester (13 h, LC-MS control). Subsequently, $MeONH_2$xHCl (26.51 mg, 317.4 µmol) was added and the pH was adjusted to pH = 3.5 using a microelectrode. Ring opening of the thiazolidine was complete after 24 h according to LC-MS (LC-MS samples were prepared with 20 mM TCEP in 20 % MeCN/H_2O). Prior to purification, the mixture was treated with TCEP x HCl (45.5 mg, 158.7 µmol, 50 equiv.) for 30 min. Peptide **26** was purified by RP-HPLC (column [F], 25-45 % MeCN/H_2O + 0.1 % TFA, 8 mL/min.

Yield of **26**: 17.9 mg (2.35 µmol, 89.0 %). RP-HPLC-MS: column [A], 20-50 % MeCN/H_2O + 0.1 % HCOOH. ESI-MS of **26**: *m/z* (average isotopes) $C_{334}H_{554}N_{100}O_{97}S_3$ (7618.89) calculated: 1089.42 $[M+7H]^{7+}$, 953.37 $[M+8H]^{8+}$, 847.55 $[M+9H]^{9+}$, found: 1089.47 $[M+7H]^{7+}$, 953.34 $[M+8H]^{8+}$, 847.52 $[M+9H]^{9+}$.

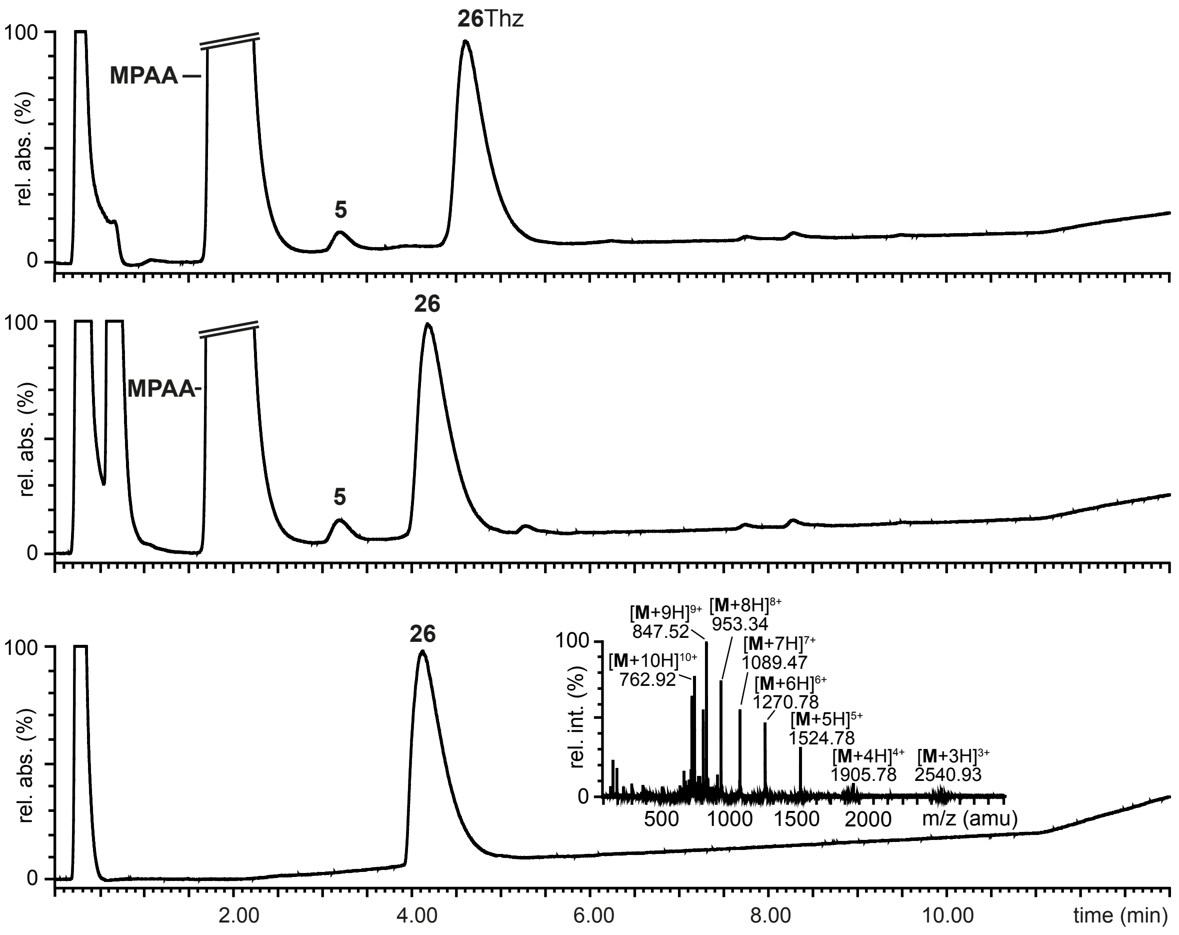

Figure S25: RP-HPLC-MS of EPO 98-166 **26**: a) reaction mixture after NCL, b) Thz opening and c) after purification by RP-HPLC.

5.5 Synthesis of EPO 29-166 Glycopeptide 27

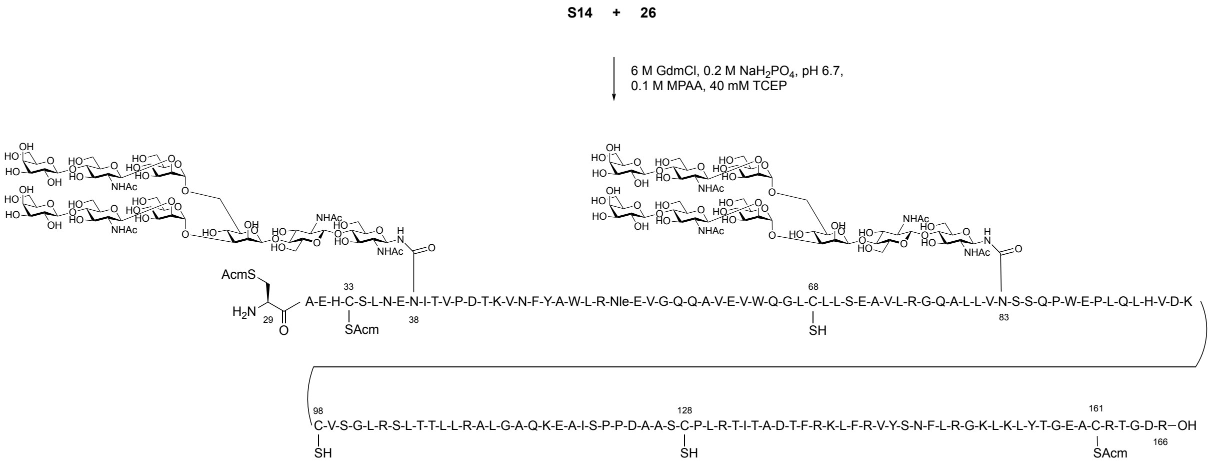

27 (73 %)

Figure S26: Synthesis of EPO 29-166 glycopeptide **27**.

The ligation buffer (6 M GdmCl, 0.2 M NaH_2PO_4, 40 mM TCEP, 0.1 M MPAA) was mixed under anaerobic conditions and adjusted to pH = 6.7. Peptide **26** (3.96 mg, 519.8 nmol, 1.2 equiv.) was dissolved in 217 µL of ligation buffer and added to the thioester **S14** (4.90 mg, 433.0 nmol, 1 equiv.). The mixture was incubated under anaerobic conditions until complete conversion of the thioester (27 h, LC-MS control, LC-MS samples were prepared with 100 mM TCEP in water).

Prior to purification, the mixture was treated with TCEP x HCl (6.2 mg, 21.6 µmol, 50 equiv.) for 30 min. Peptide **27** was purified by RP-HPLC (column [J], 30-50 % MeCN/H_2O + 0.1 % TFA, 3 mL/min) and lyophilized.

Yield of **27**: 5.93 mg (315.5 nmol, 72.9 %). RP-HPLC-MS: column [D], 20-60 % MeCN/H_2O + 0.1 % HCOOH. HR-MS of **27**: *m/z* (exact mass) $C_{812}H_{1311}N_{205}O_{291}S_6$ (18782.2414) calculated: 1879.2314 $[M+10H]^{10+}$, 1708.4838 $[M+11H]^{11+}$, 1566.1941 $[M+12H]^{12+}$, found: 1879.2380 $[M+10H]^{10+}$, 1708.4827 $[M+11H]^{11+}$, 1566.1968 $[M+12H]^{12+}$.

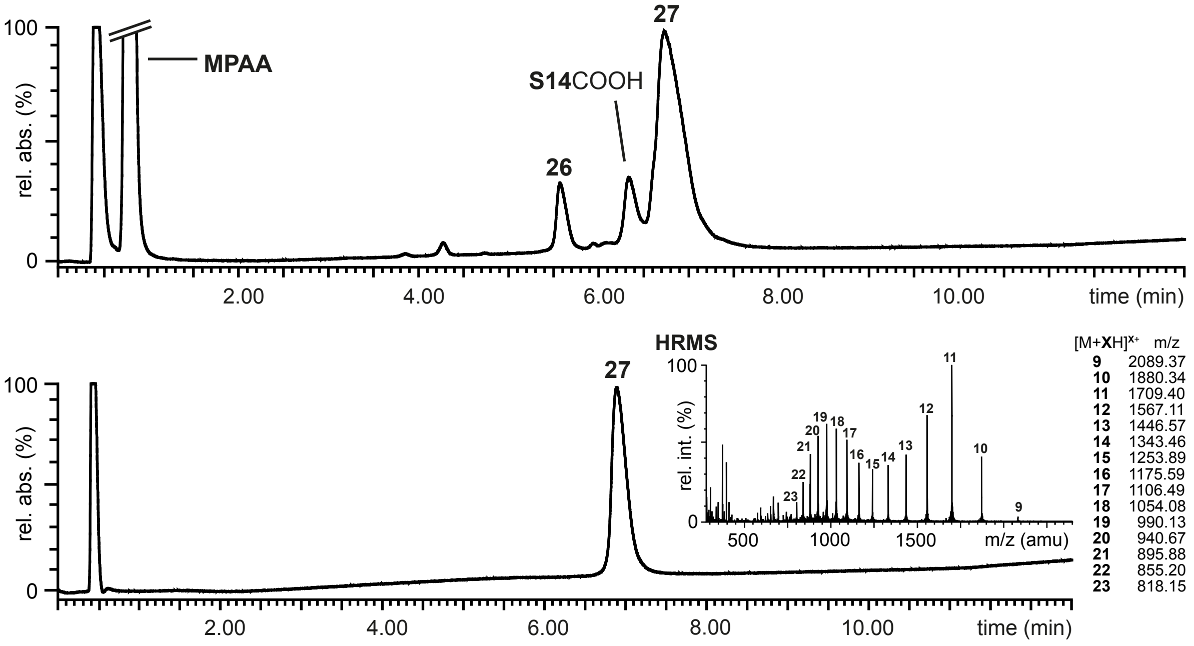

Figure S27: RP-HPLC-MS of EPO 29-166 glycopeptide **27**: a) reaction mixture after NCL and b) purified.

5.6 Desulfurization of EPO 29-166 Glycopeptide 27

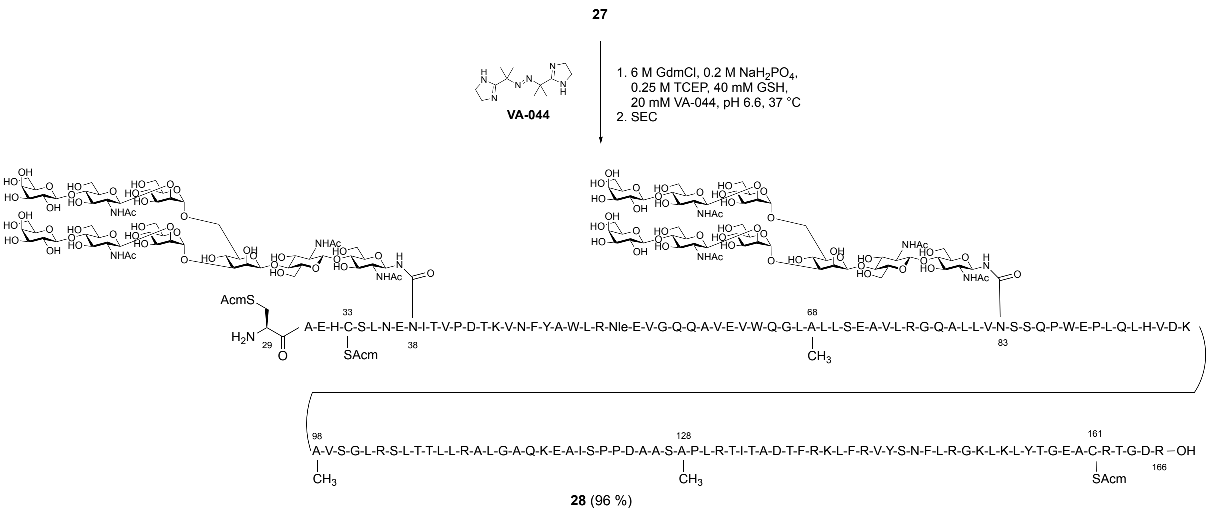

Figure S28: Synthesis of EPO 29-166 glycopeptide **28**.

Under anaerobic conditions, a buffer (6 M GdmCl, 0.2 M NaH_2PO_4) was prepared and adjusted to pH = 7.0. A TCEP stock solution was prepared with this buffer (0.5 M TCEP x HCl) and the pH was adjusted to pH 7.0. Furthermore, a glutathione stock solution (160 mM glutathione, giving pH 4.7) and a VA-044 stock solution (80 mM VA-044, giving pH 6.9) were prepared with the GdmCl buffer. The glycopeptide **27** (8.47 mg, 450.7 nmol, 1 equiv.) was dissolved in

the 450.7 µL of the TCEP stock solution (500 equiv.) followed by the glutathione and VA-044 stock solutions (225.3 µL each). The reaction mixture (final pH 6.6) was shaken under anaerobic conditions at 37 °C until completion (3 h according to HRMS). Subsequently, the reaction mixture was desalted by gel filtration (column [M], 10 % MeCN/H_2O + 0.1 % TFA, 1.5 mL/min) and lyophilized.

Yield of **28**: 8.07 mg (431.6 nmol, 95.8 %). RP-HPLC-MS: column [D], 20-60 % MeCN/H_2O + 0.1 % HCOOH. HR-MS of **28**: *m/z* (exact mass) $C_{812}H_{1311}N_{205}O_{291}S_3$ (18686.3252) calculated: 1869.6398 $[M+10H]^{10+}$, 1699.7641 $[M+11H]^{11+}$, 1558.2010 $[M+12H]^{12+}$, found: 1869.6413 $[M+10H]^{10+}$, 1699.7632 $[M+11H]^{11+}$, 1558.2028 $[M+12H]^{12+}$.

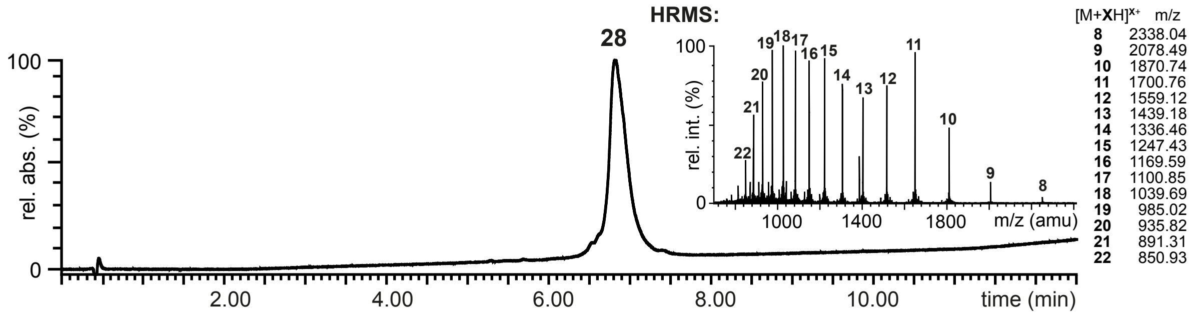

Figure S29: RP-HPLC-MS of EPO 29-166 glycopeptide **28** after SEC.

5.7 Cleavage of Acm groups from EPO 29-166 Glycopeptide 28

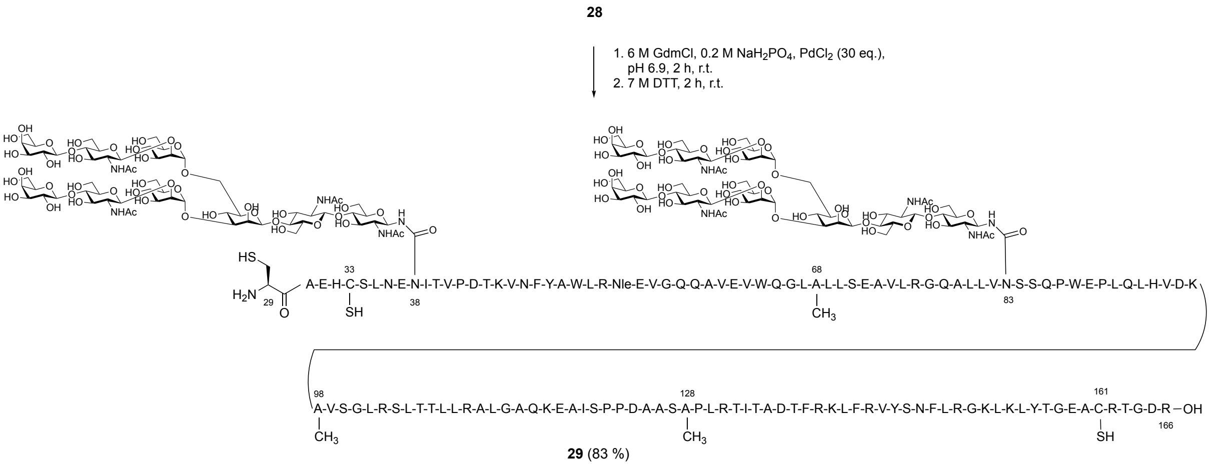

Figure S30: Synthesis of EPO 29-166 **29**.

A buffer (6 M GdmCl, 0.2 M NaH_2PO_4) was prepared and adjusted to pH = 6.9. The buffer was degassed with argon for 30 min. Subsequently, anhydrous $PdCl_2$ (2.25 mg, 12.69 µmol) was

suspended in 940 µL of this buffer and shaken for 1 h under argon for complete dissolution (13.5 mM Pd^{II}). 181 µL of the Pd^{II} stock solution (284.5 µg $PdCl_2$, 2.44 µmol, 30 equiv.) were added to the Acm-protected glycopeptide **28** (1.52 mg, 81.3 nmol, 1 equiv.) under argon. Under the exclusion of light the mixture was incubated for 2h (HPLC-MS control after adding 100 equiv. of DTT/ equiv. of Pd to an aliquot). Subsequently, DTT (195 mg) was added to the reaction mixture. The mixture was shaken for 2 h and purified by RP-HPLC (column [H], 10-60 % MeCN/H_2O + 0.1 % HCOOH, 1 mL/min). The target fractions were lyophilized. The samples for HPLC-MS of the purified glycopeptide **29** were prepared with 100 mM DTT in water.

Yield of **29**: 1.25 mg (67.62 nmol, 83.2 %). RP-HPLC-MS: column [D], 20-60 % MeCN/H_2O + 0.1 % HCOOH. HR-MS of **29**: *m/z* (exact mass) $C_{803}H_{1296}N_{202}O_{288}S_3$ (18473.2138) calculated: 1848.3287 $[M+10H]^{10+}$, 1680.3904 $[M+11H]^{11+}$, 1540.4418 $[M+12H]^{12+}$, found: 1848.3244 $[M+10H]^{10+}$, 1680.3842 $[M+11H]^{11+}$, 1540.4391 $[M+12H]^{12+}$.

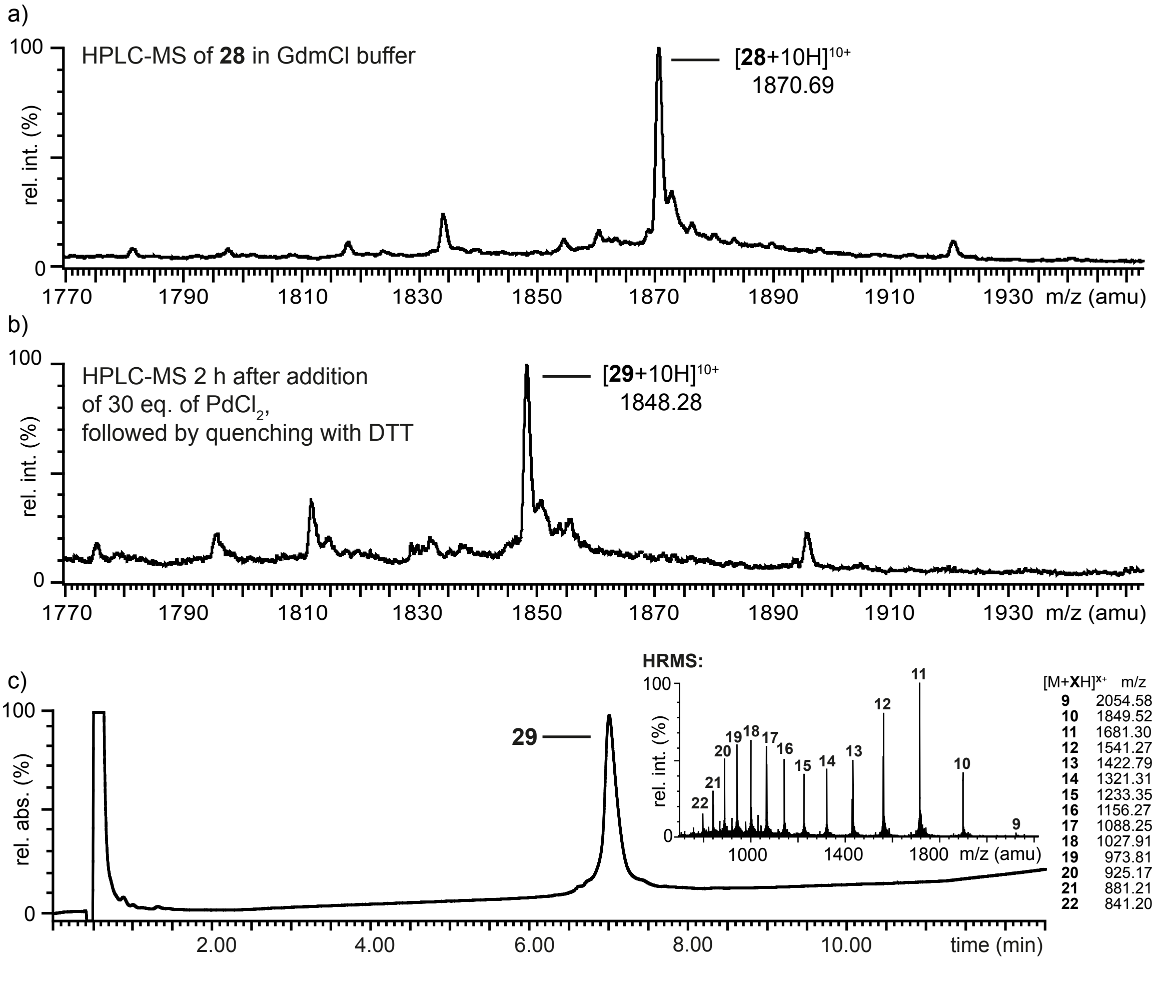

Figure S31: Synthesis of EPO 29-166 glycopeptide **29**: zoom of mass spectra (RP-HPLC-MS) a) before and b) 2 h after addition of $PdCl_2$, c) RP-HPLC-MS of **29** after RP-HPLC.

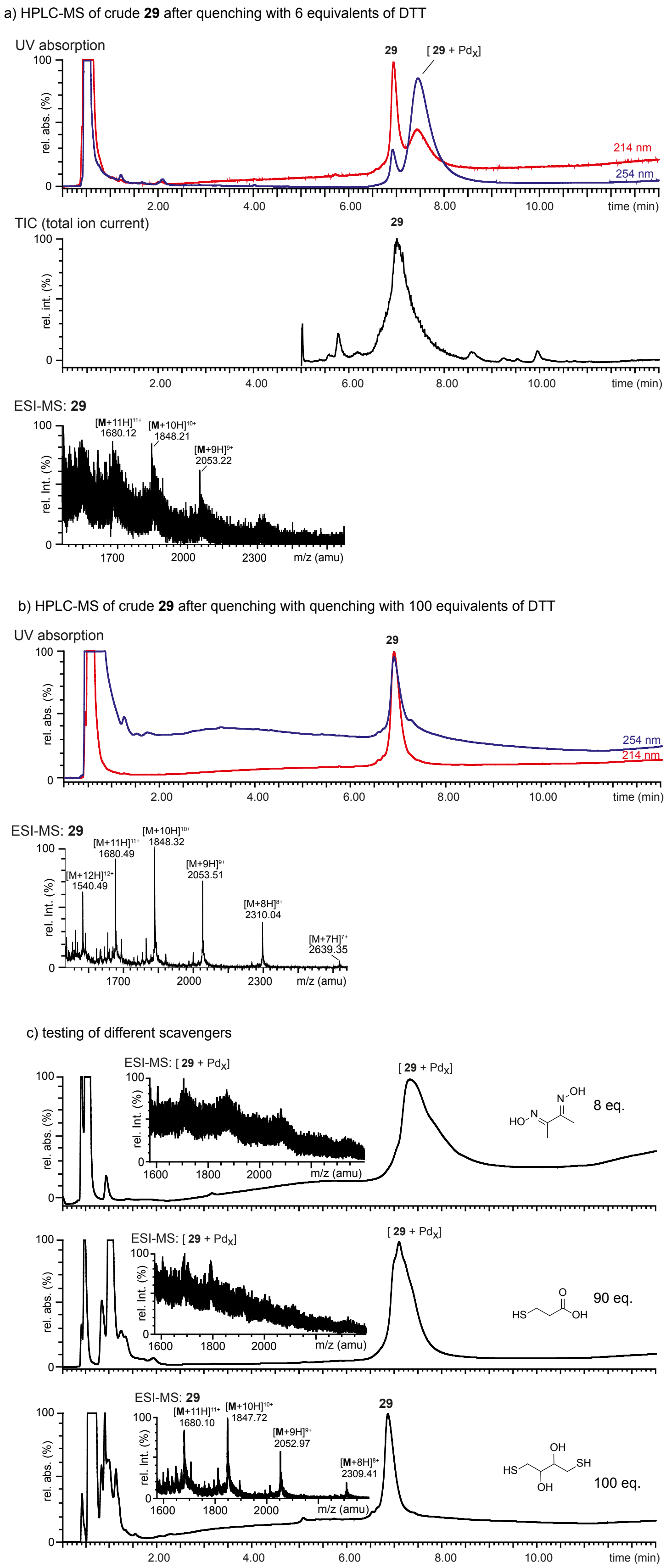

Figure S31-Pd: Deprotection of **28** with Pd(II) in GdmCl: a) analytical RP-HPLC-MS after quenching an aliquot with insufficient amounts of DTT (6 equiv. of DTT / equiv. of Pd); b) RP-HPLC-MS after quenching an aliquot with excess DTT (100 equiv. of DTT / equiv. of Pd); c) RP-HPLC-MS comparison of tested scavengers.

5.8 Synthesis of EPO 1-28 Glycopeptide Thioester 18

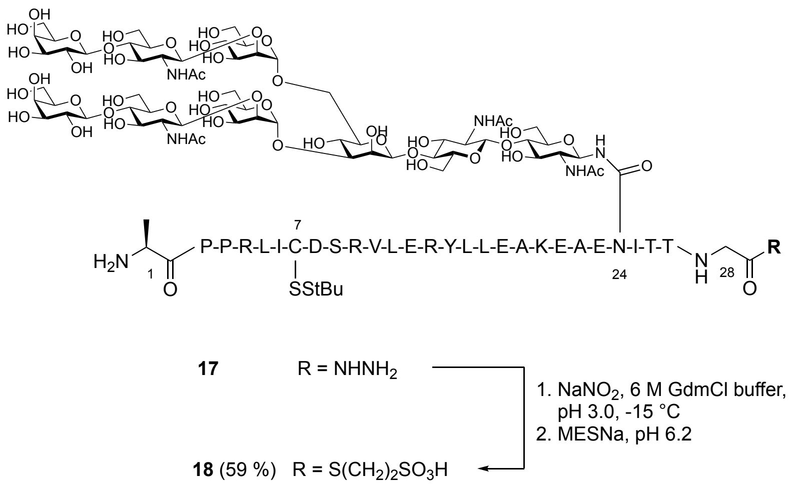

Figure S32: Synthesis of EPO 1-28 glycopeptide thioester **18**.

According to the general procedure for thioester formation, the glycopeptide hydrazide **17**[10] (5.00 mg, 1.02 µmol, 1 eq) was dissolved in 292.6 µL of 6 M GdmCl buffer and reacted with 13.31 µL of 0.5 M $NaNO_2$ (6.66 µmol, 6.5 equiv.). After 1 h, 66.5 µL of the MESNa stock solution (66.56 µmol, 65 equiv.) were added. The pH was adjusted to a value of 6.2 and the reaction mixture was purified after 1 h by RP-HPLC (column [G], 20-35 % MeCN/H_2O + 0.1 % TFA 3 mL/min). The target fractions were lyophilized.

Yield of **18**: 3.02 mg (604.6 µmol, 59.3 %). RP-HPLC-MS: column [A], 0-50 % MeCN/H_2O + 0.1 % HCOOH. ESI-MS of **18**: *m/z* (average isotopes) $C_{205}H_{343}N_{43}O_{91}S_4$ (4994.45) calculated: 2498.23 $[M+2H]^{2+}$, 1665.82 $[M+3H]^{3+}$, found: 2498.94 $[M+2H]^{2+}$, 1666.42 $[M+3H]^{3+}$.

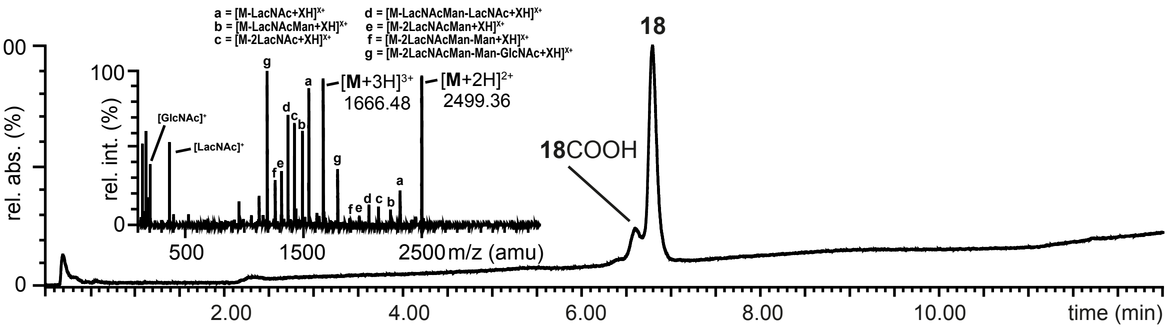

Figure S33: RP-HPLC-MS of purified EPO 1-28 glycopeptide Thioester **18**.

5.9 Final Ligation and Folding of EPO 1-166 (EPO A)

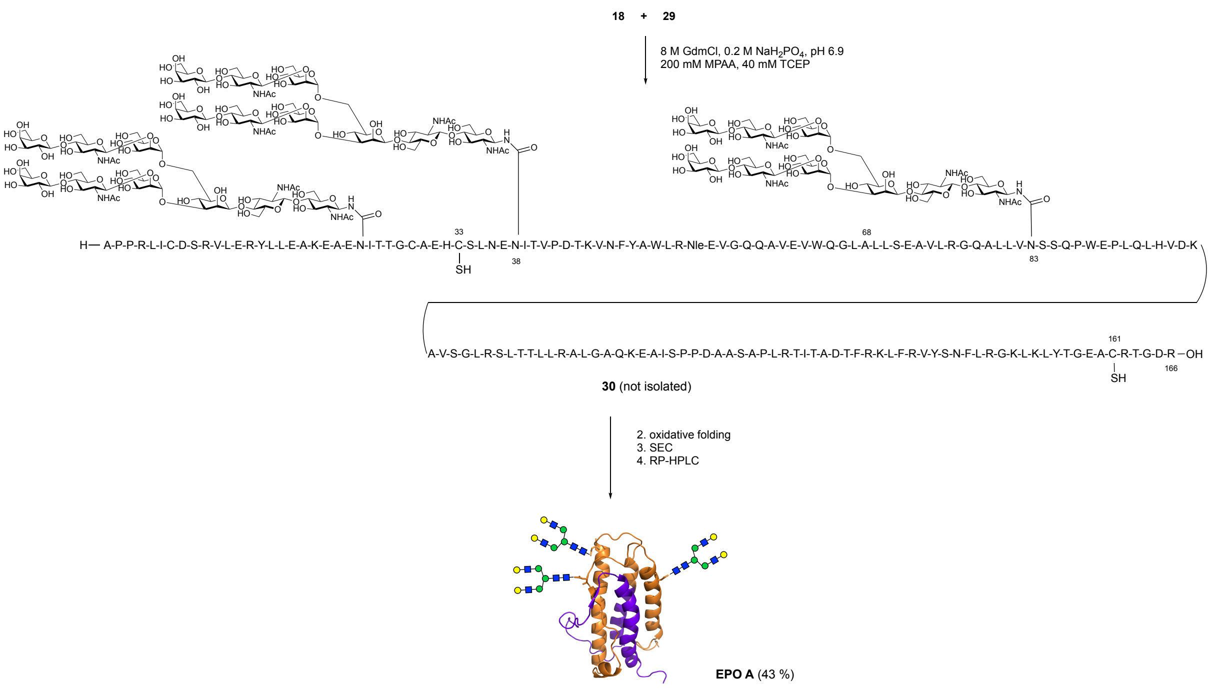

Figure S34: Synthesis and refolding of **EPO A**

The ligation buffer (8 M GdmCl, 0.2 M NaH_2PO_4, 40 mM TCEP, 0.1 M MPAA) was mixed under anaerobic conditions and adjusted to pH = 6.9. The glycopeptide thioester **18** (0.67 mg, 133.96 nmol, 2.5 equiv.) was dissolved in 27 µL of ligation buffer and added to glycopeptide **29** (1.01 mg, 53.59 nmol, 1 equiv.). The mixture was incubated under anaerobic conditions until complete conversion of the thioester (6 d, LC-MS control). The ligation mixture was diluted with 12.46 mL of the dilution buffer (Table S5) and transferred to a tubular dialysis membrane (ZelluTrans/Roth V series, cutoff = 5 kDa, 40 mm flat width, thickness 20-30 µm, regenerated cellulose), and then dialyzed sequentially against the buffers shown in Table S5 (500 mL each) with constant agitation. The dialysis conditions are listed in Table S6.

Table S5:

Buffer	Components				
Dilution Buffer (degassed, pH 7.5)	8 M GdmCl (98 %)	0.1 M NaH_2PO_4	0.5 M L-Arg (98.5 %)	0.1 M DTT	
Reduction Buffer (degassed, pH 8.5)	4 M GdmCl (98 %)	0.1 M NaH_2PO_4	0.5 M L-Arg (98.5 %)	0.01 M DTT	
Oxidation Buffer 4 M (pH 8.5)	4 M GdmCl (98 %)	0.1 M NaH_2PO_4	0.5 M L-Arg (98.5 %)	4 mM L-Cys	0.5 mM L-Cystine

Oxidation Buffer 2 M (pH 8.5)	2 M GdmCl (98 %)	0.1 M NaH_2PO_4	0.5 M L-Arg (98.5 %)	4 mM L-Cys	0.5 mM L-Cystine
Folding Buffer 1 (pH 8.0)	1 M GdmCl (>99.5 %)	0.1 M NaH_2PO_4	0.5 M L-Arg (98.5 %)		
Folding Buffer 2 (pH 7.5)	0.15 M NaCl	0.05 M NaH_2PO_4	0.5 M L-Arg (98.5 %)		

Table S6:

Buffer	Conditions	Dialysis Duration
Reduction Buffer	anaerobic (≤ 5 ppm O_2), 26 °C	12 h
Oxidation Buffer 4 M	air, 4 °C	12 h
Oxidation Buffer 2 M	air, 4 °C	12 h
Folding Buffer 1	air, 4 °C	8 h
Folding Buffer 2	air, 4 °C	8 h
Folding Buffer 2	air, 4 °C	8 h
Folding Buffer 2	air, 4 °C	8 h

After completion of the dialysis the protein solution was concentrated to a volume of 1.25 mL (Amicon 8010 stirred cell with membrane UP010 P from MICRODYN-NADIR, cutoff = 10 kDa). Monomeric **EPO A** was isolated by size exclusion chromatography (column [P], 0.15 M NaCl, 50 mM NaH_2PO_4, 0.5 M L-arginine, pH 7.5, 1 mL/min). The eluate was concentrated by ultrafiltration to a volume of 1.25 mL, purified by RP-HPLC (column [H], 30-50 % MeCN/H_2O + 0.1 % TFA, 1 mL/min) and lyophilized.
Yield of **EPO A**: 0.54 mg (23.2 nmol, 43.3 %).
ICP-OES (λ (nm) = 340.458, 363.470, 324.270, 248.892): Pd ≤ 0.01 ppm.
RP-HPLC-MS: column [D], 20-60 % MeCN/H_2O + 0.1 % HCOOH.
HR-MS of **EPO A**: *m/z* (exact mass) $C_{1002}H_{1621}N_{245}O_{376}S_4$ (23230.4137) calculated: 1660.3225 $[M+14H]^{14+}$, 1549.7015 $[M+15H]^{15+}$, 1452.9081 $[M+16H]^{16+}$, found: 1660.3261 $[M+14H]^{14+}$, 1549.7033 $[M+15H]^{15+}$, 1452.9103 $[M+16H]^{16+}$.

The CD spectrum was recorded with 250 µL of **EPO A** (0.22 mg **EPO A**/mL) in ultrapure water at 23 °C.

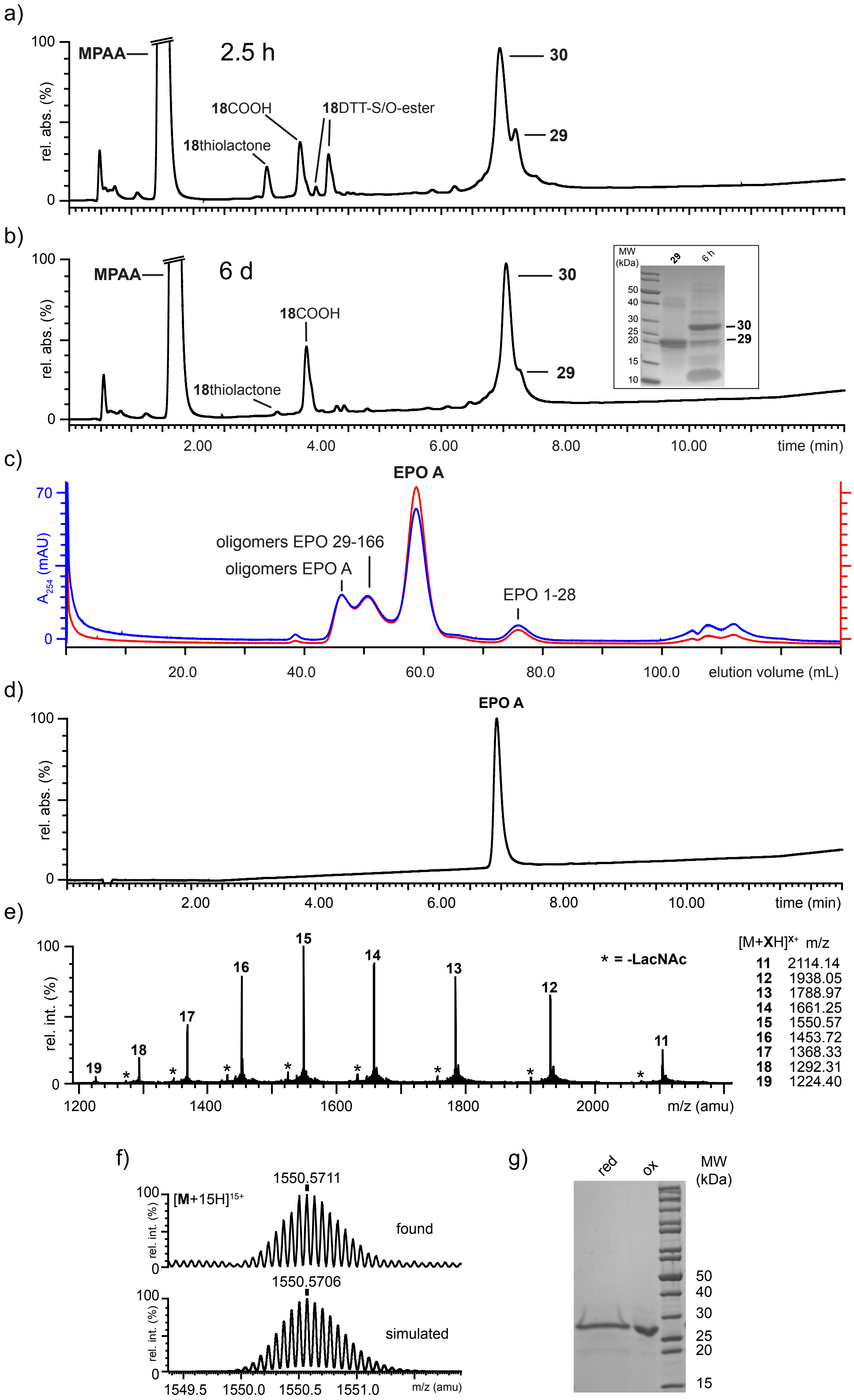

Figure S35: Synthesis and purification of **EPO A**: a) RP-HPLC-MS of crude NCL mixture after 2.5 h, b) RP-HPLC-MS and SDS-PAGE of crude NCL mixture after 6 d, c) Separation of mono- and oligomers by gelfiltration (Superdex 75), d) RP-HPLC-MS of **EPO A**, e) HRMS of **EPO A**, f) found and simulated HR-isotope patterns of $[M+15H]^{15+}$ charge state, g) SDS-PAGE of **EPO A** reduced and oxidized.

6. Synthesis of Sialylated EPO S by Sequential NCL

6.1 Synthesis of Sialylated EPO 1-28 Glycopeptide Hydrazide 31

17

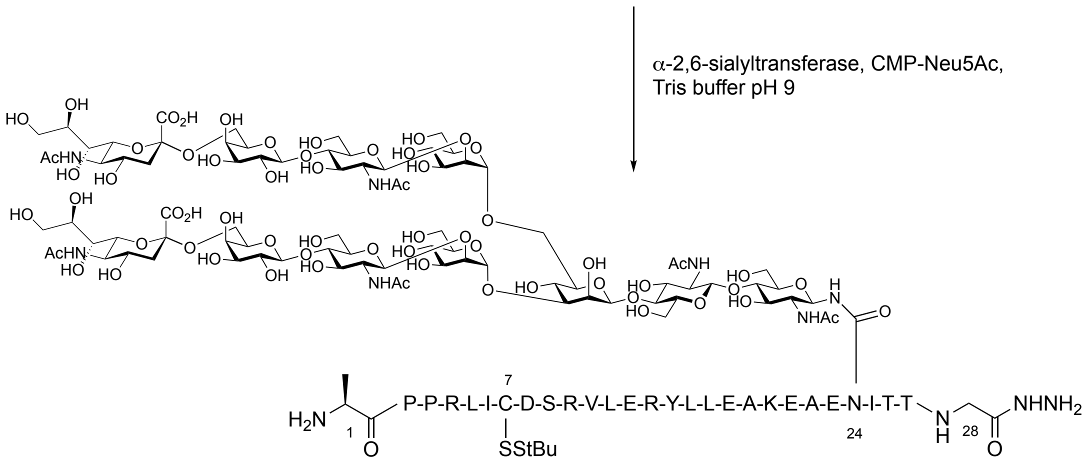

31 (67 %)

Figure S36: Synthesis of sialylated EPO 1-28 glycopeptide hydrazide **31**

The glycopeptide hydrazide **17** (3.0 mg, 0.61 μmol, 1.0 equiv.) was dissolved in 36.6 μL of water and 49.2 μL of a stock solution containing 1.5 mg CMP-Neu5Ac (2.4 μmol, 3.9 eq) in 250 mM Tris, pH 9 was added. Subsequently, 37.1 μL of α-2,6-sialyltransferase (107.59 μg) from *P. damselae* were added. The solution was incubated at 30 °C. After 2 h 30.7 μL of the CMP-Neu5Ac stock solution were added. After incubation for 1h at 30 °C, **31** was isolated from the mixture by RP-HPLC (column [G], 5-30 % MeCN/H_2O + 0.1 % HCOOH, 3 mL/min). The target fractions were neutralized with Na_2CO_3 (1 M, approximately 20 μL/mL of eluate) and lyophilized. The residue was dissolved in 1.0 mL of 5 % EtOH/water and desalted (column [N], 5 % EtOH/water, 2 mL/min). The target fraction was lyophilized.

Yield of **31**: 2.25 mg (0.41 μmol, 67.2 %). RP-HPLC-MS: column [A], 10-50 % MeCN/H_2O + 0.1 % HCOOH. HR-MS of **31**: *m/z* (exact mass) $C_{225}H_{375}N_{47}O_{104}S_2$ (5463.4941) calculated: 1364.8663 $[M{-}4H]^{4-}$, 1820.1574 $[M{-}3H]^{3-}$, 2730.7398 $[M{-}2H]^{2-}$; found: 1364.8630 $[M{-}4H]^{4-}$, 1820.1544 $[M{-}3H]^{3-}$, 2730.7117 $[M{-}2H]^{2-}$, 5462.4869 $[M{-}H]^{-}$;

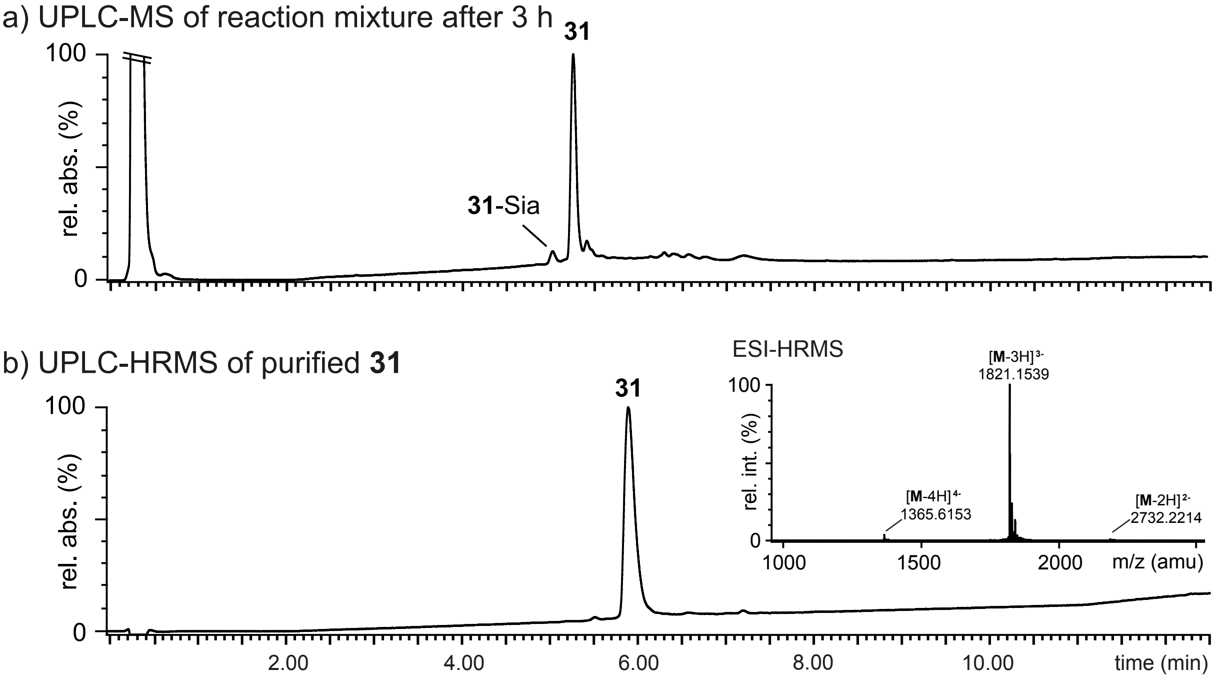

Figure S37: Enzymatic Sialylation of EPO 1-28 hydrazide **17**: RP-HPLC-MS of a) crude reaction mixture after 3h and b) purified EPO 1-28 sialoglycopeptide **31**.

6.2 Synthesis of Sialylated EPO 29-67 Glycopeptide Hydrazide 32

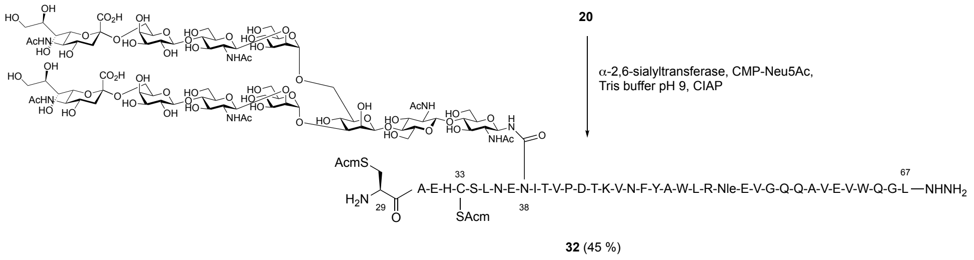

Figure S38: Synthesis of sialylated EPO 29-67 glycopeptide hydrazide **32**

The glycopeptide hydrazide **20** (8.00 mg, 1.28 µmol, 1 equiv.) was dissolved in 1.017 mL of Tris buffer (250 mM, pH 9) and 701.4 µL of a stock solution containing CMP-Neu5Ac (23.0 mg, 30.7 µmol, 24 equiv.). Subsequently, 11.58 µL of calf intestinal alkaline phosphatase (100 mU/µL) and 57.33 µL of α-2,6-sialyltransferase (166.3 µg) from *P. damselae* were added. The solution was incubated at 30 °C. After 26 h the reaction mixture was desalted (column [M], 10 % MeCN/H_2O, 0.5 mL/min), lyophilized, and the sialoglycopeptide **32** was purified by RP-HPLC (column [G], 10-30 % MeCN/H_2O + 0.1 % HCOOH, 3 mL/min). The target fractions were neutralized with Na_2CO_3 (1 M, approximately 20 µL/mL of eluate) and lyophilized. The residue was dissolved in 1.5 mL of 10 % MeCN/H_2O and desalted (column [M], 10 % MeCN/H_2O, 0.5 mL/min). The target fraction was lyophilized.

Yield of **32**: 3.97 mg (0.58 µmol, 45.3 %). RP-HPLC-MS: column [A], 10-40 % MeCN/H_2O + 0.1 % HCOOH. ESI-MS of **32**: *m/z* (average isotopes) $C_{289}H_{452}N_{64}O_{122}S_2$ (6839.24) calculated: 2278.74 $[M-3H]^{3-}$, 1708.80 $[M-4H]^{4-}$, 1366.84 $[M-5H]^{5-}$; found: 2281.07 $[M-3H]^{3-}$, 1710.54 $[M-4H]^{4-}$, 1368.14 $[M-5H]^{5-}$.

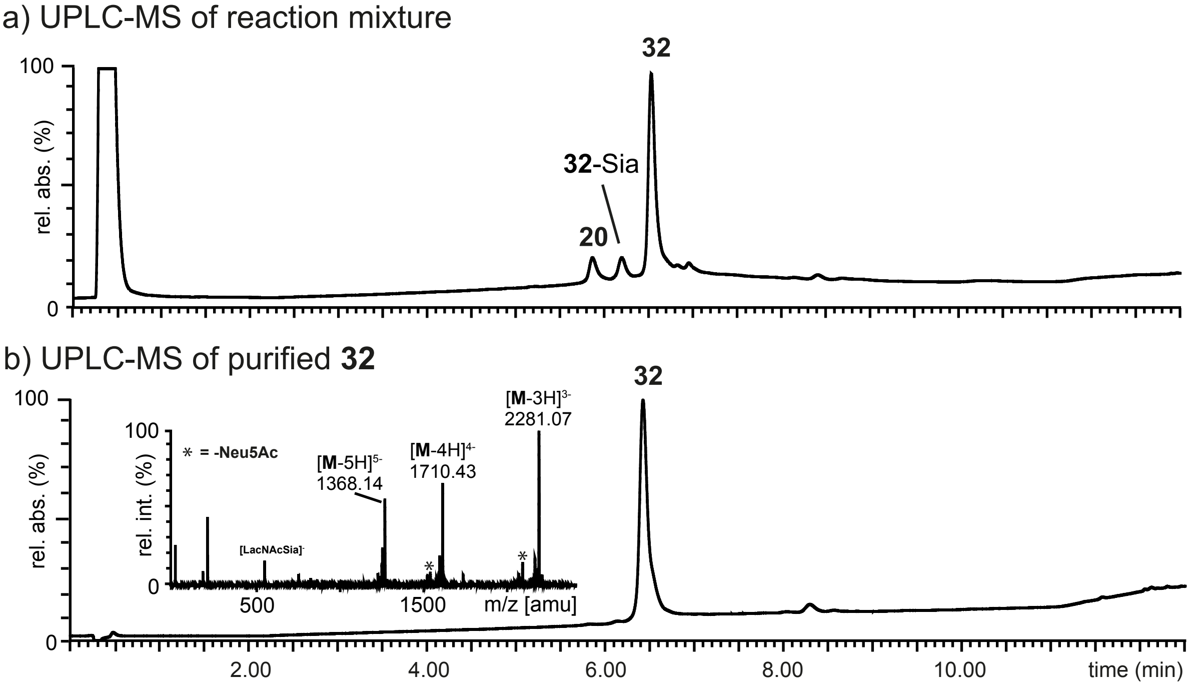

Figure S39: Enzymatic Sialylation of EPO 29-67 glycopeptide hydrazide **20**: RP-HPLC-MS of a) crude reaction mixture and b) EPO 29-67 sialoglycopeptide **32** after RP-HPLC.

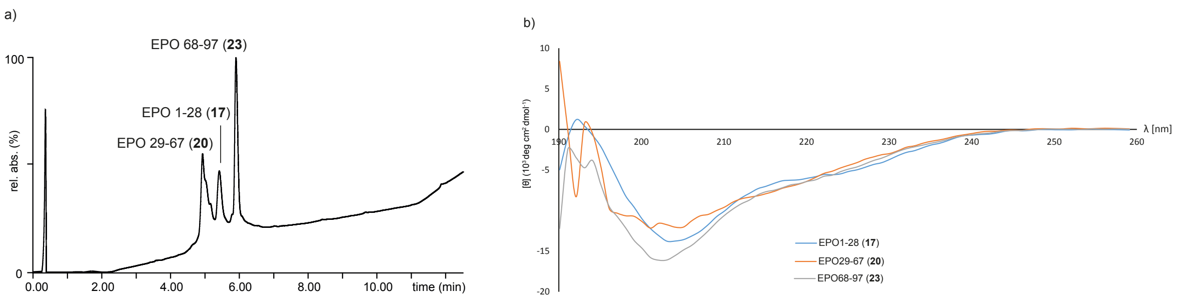

Figure S39a: a) Comparison of the hydrophilicity of EPO glycopeptide hydrazides 1-28 (**17**), 29-67 (**20**) and 68-97 (**23**) by RP-HPLC-MS (column [A], 10-50 % MeCN/H_2O + 0.1 % HCOOH); b) Superposition of CD-spectra of EPO glycopeptide hydrazides 1-28 (**17**), 29-67 (**20**) and 68-97 (**23**).

Table S7: conditions tested for the optimization of the sialylation of glycopeptide **20**

				product distribution after 16 h of reaction time from integrated HPLC-trace (UV)		
	[**20**]	CMP-Neu5Ac	PdST6	**20**	**32-Sia**	**32**
1)	5 mM	4 + 4 + 2 eq.	130 ng/nmol	26 %	22 %	52 %
2)	4 mM	1 x 24 eq.	130 ng/nmol	14 %	20 %	66 %
3)	1 mM	1x 24 eq.	130 ng/nmol	18 %	14 %	68 %
4)	1 mM	6 x 4 eq./h	130 ng/nmol	12 %	19 %	69 %
5)	1 mM	1 x 24 eq.	260 ng/nmol	30 %	15 %	55 %
6)	0.5 mM	1 x 24 eq.	130 ng/nmol	6 %	7 %	87 %
7)	0.5 mM	1 x 12 eq.	130 ng/nmol	12 %	8 %	80 %

6.3 Synthesis of Sialylated EPO 68-97 Glycopeptide Hydrazide 33

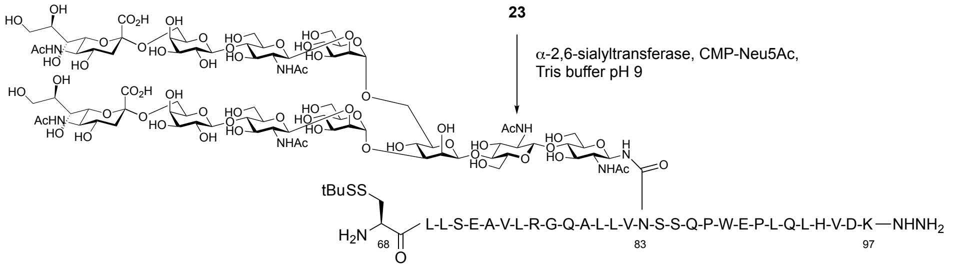

33 (82 %)

Figure S40: Synthesis of sialylated EPO 68-97 glycopeptide hydrazide **33**.

The glycopeptide hydrazide **23** (2.0 mg, 394.4 nmol, 1 equiv.) was dissolved in 29.65 µL of water and 31.56 µL of a stock solution containing 1.15 mg CMP-Neu5Ac (1.58 µmol, 4 equiv.) in 250 mM Tris, pH 9 was added. Subsequently, 17.68 µL of α-2,6-sialyltransferase (51.27 µg) from *P. damselae* were added. The solution was incubated at 30 °C. After 14h 1.15 mg of CMP-Neu5Ac (1.58 µmol, 4 equiv.) in 9µL of 250 mM Tris pH 9 were added. The solution was incubated for 3 and subsequently **33** was purified by RP-HPLC (column [G], 20-50 % MeCN/H_2O + 0.1 % HCOOH, 3 mL/min). The target fractions were neutralized with Na_2CO_3 (1 M, approximately 20 µL/mL of eluate) and lyophilized. The residue was dissolved in 1.2 mL of 10 % MeCN/H_2O and desalted (column [M], 10 % MeCN/H_2O, 0.5 mL/min). The target fraction was lyophilized.

Yield of **33**: 1.82 mg (322 nmol, 81.6 %). RP-HPLC-MS: column [A], 10-50 % MeCN/H_2O +

0.1 % HCOOH. ESI-MS of **33**: *m/z* (average isotopes) $C_{237}H_{389}N_{49}O_{104}S_2$ (5653.08) calculated: 1883.35 $[M-3H]^{3-}$, 1412.26 $[M-4H]^{4-}$; found: 1880.54 $[M-3H]^{3-}$, 1410.15 $[M-4H]^{4-}$.

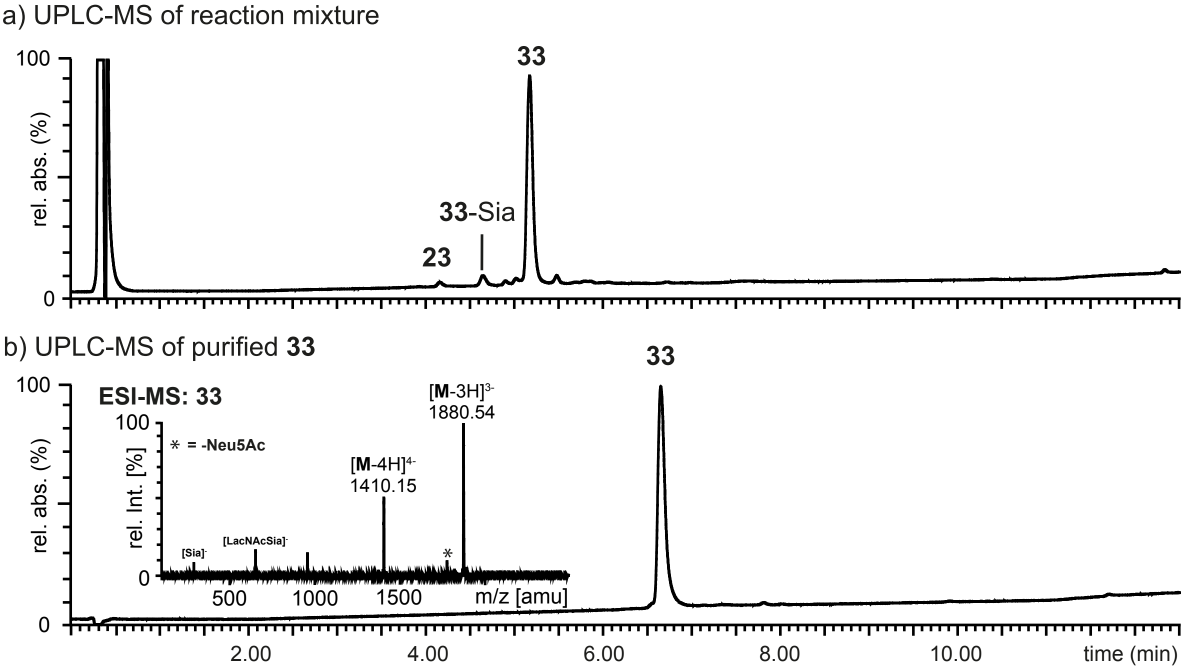

Figure S41: Enzymatic Sialylation of EPO 68-97 hydrazide **21**: RP-HPLC-MS of a) crude reaction mixture and b) EPO 68-97 sialoglycopeptide **33** after RP-HPLC.

6.4 Synthesis of EPO 1-28 Sialoglycopeptide Thioester 34

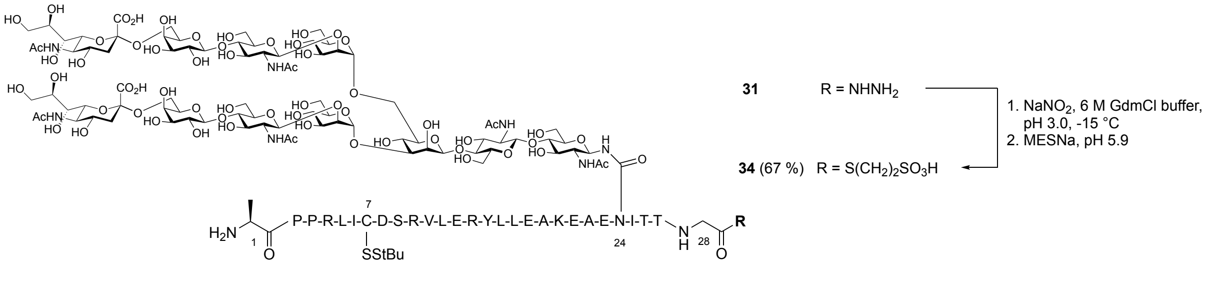

Figure S42: Synthesis of EPO 1-28 glycopeptide thioester **34**.

According to the general procedure for thioester formation, the sialoglycopeptide hydrazide **31** (3.55 mg, 649.4 nmol, 1 equiv.) was dissolved in 185.5 µL of 6 M GdmCl buffer and reacted with 8.44 µL of 0.5 M $NaNO_2$ (4.22 µmol, 6.5 equiv.). After 1 h, 42.2 µL of the MESNa stock solution (42.21 µmol, 65 equiv.) were added. The pH was adjusted to a value of 5.9 and the reaction mixture was purified after 1 h by RP-HPLC (column [G], 25-35 % MeCN/H_2O + 0.1 % HCOOH, 3 mL/min). The target fractions were immediately frozen and lyophilized.

Yield of **34**: 2.42 mg (433.9 nmol, 66.8 %). RP-HPLC-MS: column [A], 0-50 % MeCN/H_2O + 0.1 % HCOOH. ESI-MS of **34**: *m/z* (average isotopes) $C_{227}H_{377}N_{45}O_{107}S_4$ (5576.96) calculated: 1857.98 $[M\text{-}3H]^{3-}$, 1393.23 $[M\text{-}4H]^{4-}$, found: 1858.67 $[M\text{-}3H]^{3-}$, 1393.77 $[M\text{-}4H]^{4-}$.

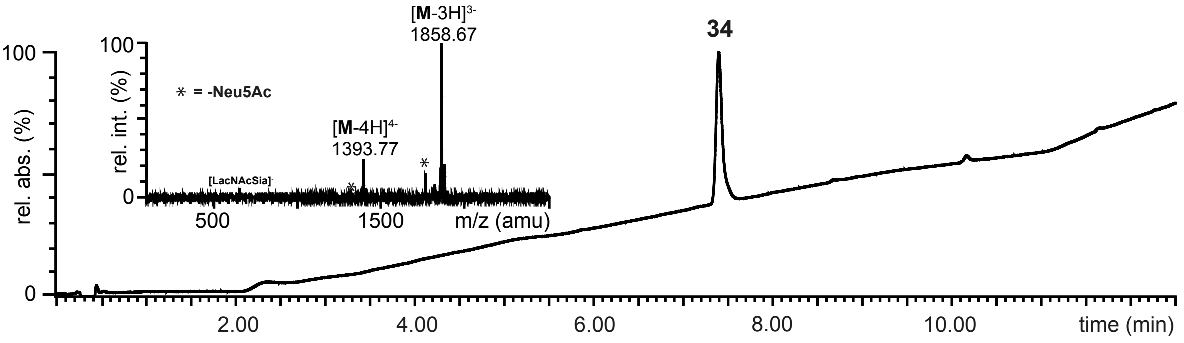

Figure S43: RP-HPLC-MS of purified EPO 1-28 sialoglycopeptide thioester **34**.

6.5 Synthesis of EPO 29-67 Sialoglycopeptide Thioester 35

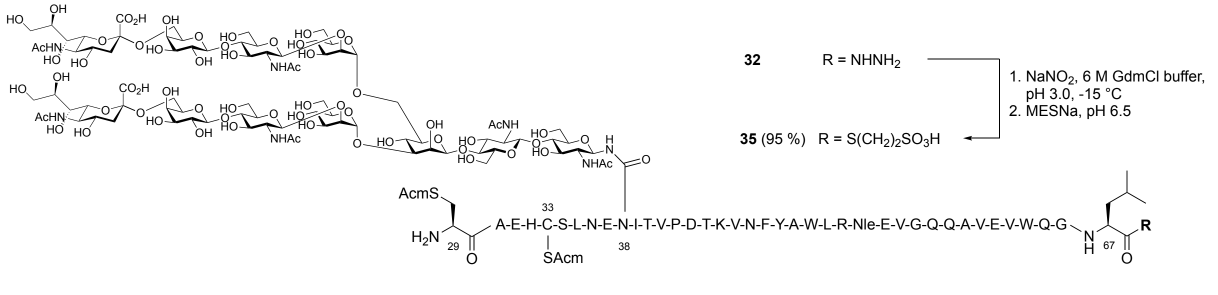

Figure S44: Synthesis of EPO 29-67 glycopeptide thioester **35**.

According to the general procedure for thioester formation, the sialoglycopeptide hydrazide **32** (3.97 mg, 580.5 nmol, 1 equiv.) was dissolved in 165.9 µL of 6 M GdmCl buffer and reacted with 7.55 µL of 0.5 M $NaNO_2$ (3.77 µmol, 6.5 equiv.). After 1 h, 37.73 µL of the MESNa stock solution (42.21 µmol, 65 equiv.) were added. The pH was adjusted to a value of 6.5 and the reaction mixture was purified after 1 h by gel filtration (column [M], 10 % MeCN/H_2O, 0.5 mL/min). The target fraction was lyophilized.

Yield of **35**: 3.83 mg (551.1 nmol, 94.9 %). RP-HPLC-MS: column [A], 10-40 % MeCN/H_2O + 0.1 % HCOOH. ESI-MS of **35**: *m/z* (average isotopes) $C_{291}H_{454}N_{62}O_{125}S_4$ (6949.38) calculated: 2315.45 $[M\text{-}3H]^{3-}$, 1736.34 $[M\text{-}4H]^{4-}$, 1388.87 $[M\text{-}5H]^{5-}$;
found: 2315.78 $[M\text{-}3H]^{3-}$, 1736.67 $[M\text{-}4H]^{4-}$, 1388.89 $[M\text{-}5H]^{5-}$.

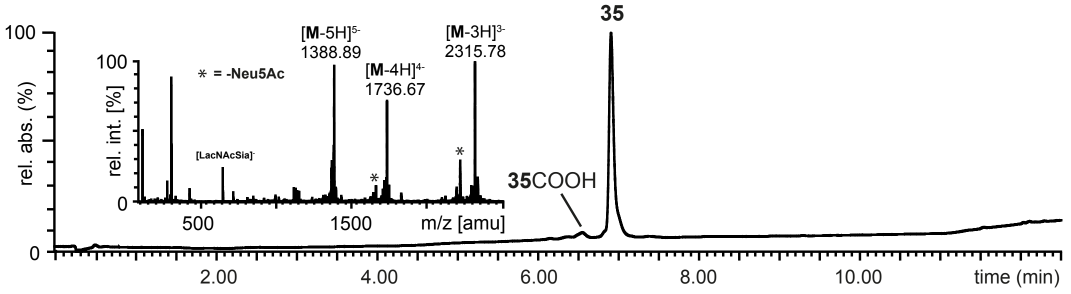

Figure S45: RP-HPLC-MS of purified EPO 29-67 sialoglycopeptide thioester **35**.

6.6 Synthesis of EPO 29-97 Sialoglycopeptide Hydrazide 36

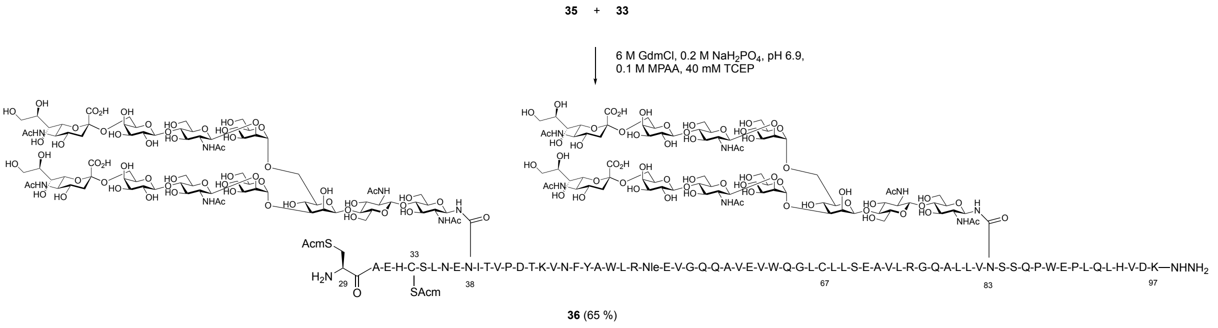

Figure S46: Synthesis of EPO 29-97 glycopeptide hydrazide **36**.

The ligation buffer (6 M GdmCl, 0.2 M NaH_2PO_4, 40 mM TCEP, 0.1 M MPAA) was mixed under anaerobic conditions and adjusted to pH = 6.9. The sialoglycopeptide hydrazide **33** (3.12 mg, 551.1 nmol, 1 equiv.) was dissolved in 275 µL of ligation buffer and added to the thioester **35** (3.12 mg, 551.1 µmol, 1 equiv.). The mixture was incubated under anaerobic conditions until complete conversion of the thioester (14 h, LC-MS control, LC-MS samples were prepared with 100 mM DTT in water).

Prior to purification, 4.63 mg of DTT in 24.4 µL of 6 M GdmCl, 0.2 M NaH_2PO_4, pH 6.9 was added to the reaction mixture. The glycopeptide hydrazide **36** was isolated by RP-HPLC (column [G], 20-50 % MeCN/H_2O + 0.1 % HCOOH, 3 mL/min). Target fractions were neutralized with 1 M Na_2CO_3 (approximately 20 µL/mL eluate) and lyophilized. The residue was taken up in 900 µL of 10 % MeCN/H_2O containing 100 mM DTT, and desalted (column [M], 10 % MeCN/H_2O, 0.5 mL/min). The target fraction was lyophilized.

Yield of **36**: 4.45 mg (359.7 nmol, 65.3 %). RP-HPLC-MS: column [A], 20-60 % MeCN/H_2O

+ 0.1 % HCOOH. HR-MS of **36**: *m/z* (exact mass) $C_{522}H_{829}N_{111}O_{226}S_3$ (12364.5951) calculated: 1374.8512 $[M+9H]^{9+}$, 1237.4668 $[M+10H]^{10+}$, 1125.0614 $[M+11H]^{11+}$, found: 1374.8576 $[M+9H]^{9+}$, 1237.4743 $[M+10H]^{10+}$, 1125.0667 $[M+11H]^{11+}$.

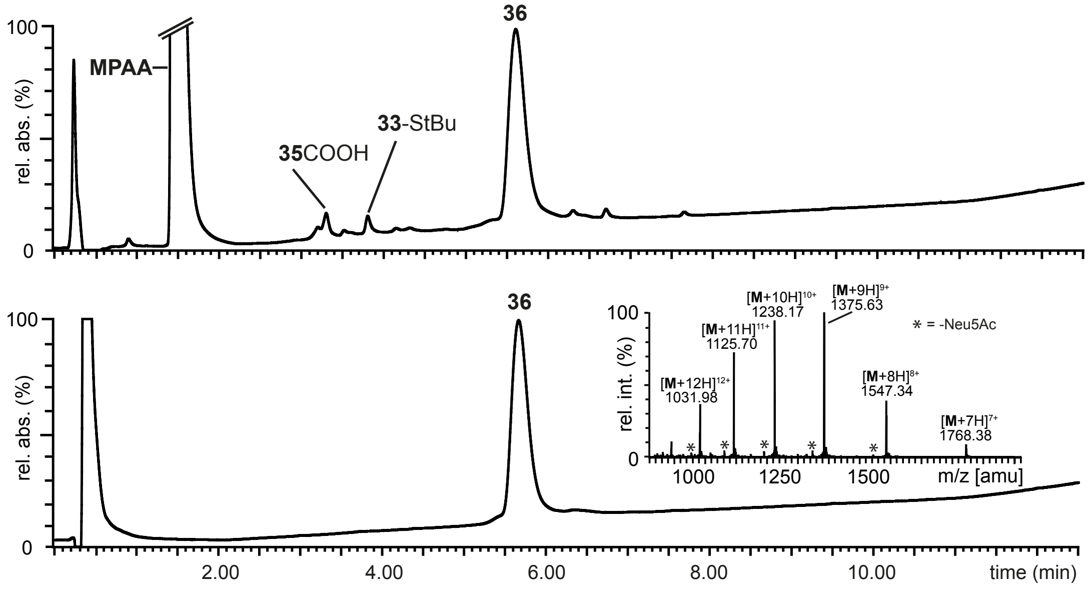

Figure S47: RP-HPLC-MS of EPO 29-97 sialoglycopeptide thioester **36** a) crude after 14 h, b) after RP-HPLC.

6.7 Synthesis of EPO 29-97 Sialoglycopeptide Thioester S15

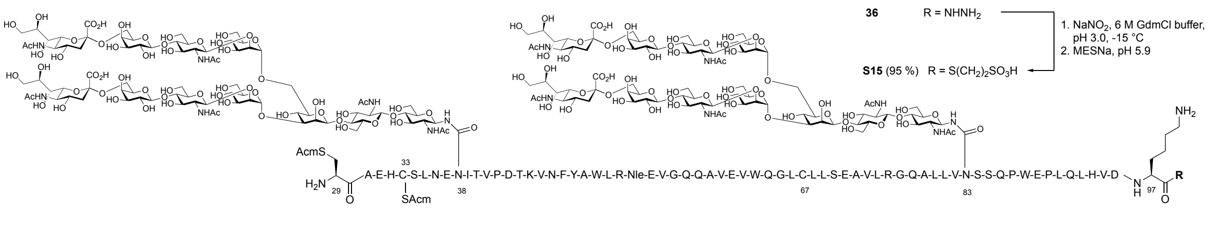

Figure S48: Synthesis of EPO 29-97 glycopeptide thioester **S15**.

According to the general procedure for thioester formation, the glycopeptide hydrazide **36** (5.50 mg, 444.6 nmol, 1 equiv.) was dissolved in 127.0 µL of 6 M GdmCl buffer and reacted with 5.78 µL of 0.5 M $NaNO_2$ (2.89 µmol, 6.5 equiv.). After 1 h, 28.9 µL of the MESNa stock solution (28.90 µmol, 65 equiv.) were added. The pH was adjusted to a value of 5.9 and the reaction mixture was purified after 1 h by gel filtration (column [M], 10 % MeCN/H_2O, 0.5 mL/min). The target fraction was lyophilized. LC-MS samples were prepared with 40 mM TCEP (water, pH 7).

Yield of **S15**: 5.29 mg (423.8 nmol, 95.3 %). RP-HPLC-MS: column [A], 20-60 % MeCN/H_2O + 0.1 % HCOOH. HR-MS of **S15**: *m/z* (exact mass) $C_{524}H_{831}N_{109}O_{229}S_5$ (12474.5335)

calculated: 1783.0835 $[M+7H]^{7+}$, 1560.3240 $[M+8H]^{8+}$, 1387.0666 $[M+9H]^{9+}$, 1248.4606 $[M+10H]^{10+}$, found: 1783.0852 $[M+7H]^{7+}$, 1560.3240 $[M+8H]^{8+}$, 1387.0648 $[M+9H]^{9+}$, 1248.4578 $[M+10H]^{10+}$.

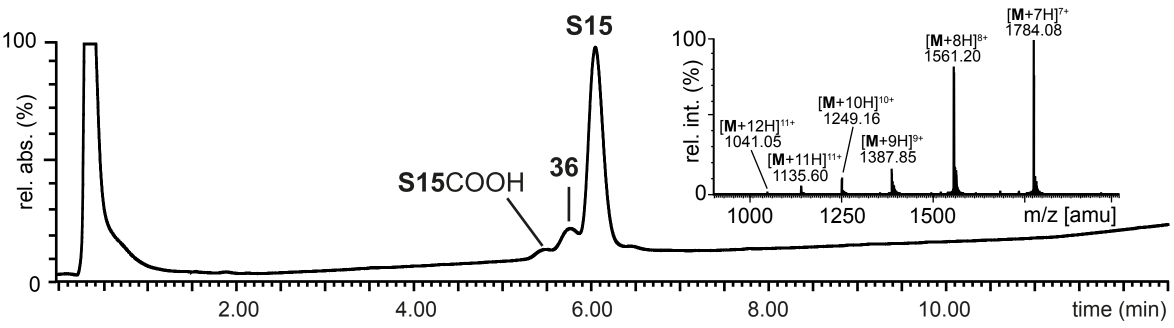

Figure S49: RP-HPLC-MS of EPO 29-97 sialoglycopeptide thioester **S15** after SEC.

6.8 Synthesis of EPO 29-166 Sialoglycopeptide 38

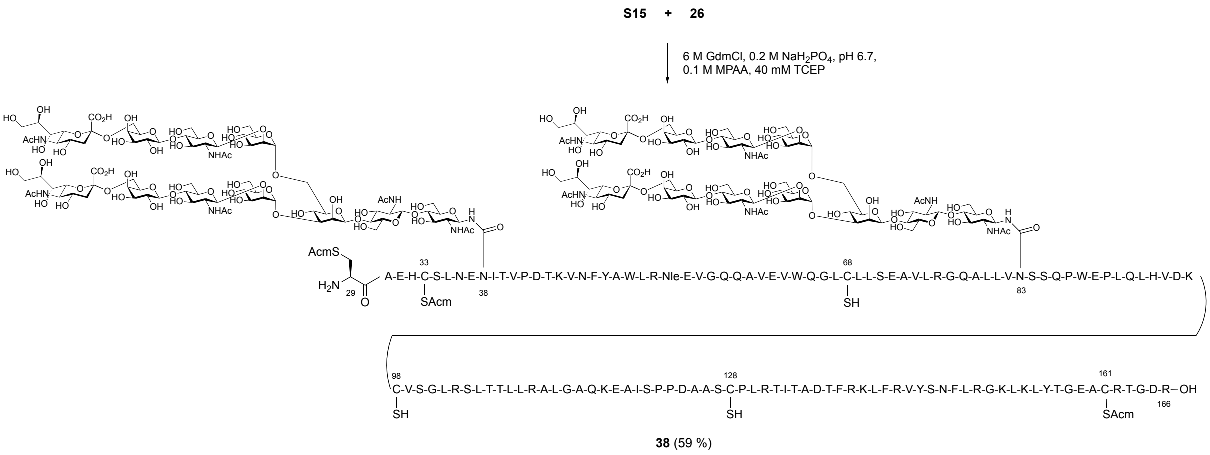

Figure S50: Synthesis of EPO 29-166 glycopeptide **38**.

The ligation buffer (6 M GdmCl, 0.2 M NaH_2PO_4, 40 mM TCEP, 0.1 M MPAA) was mixed under anaerobic conditions and adjusted to pH = 6.7.

The peptide **26** (1.12 mg, 147.1 nmol, 1.2 equiv.) was dissolved in 61.3 µL of ligation buffer and added to the thioester **S15** (1.53 mg, 122.6 nmol, 1 equiv.). The mixture was incubated under anaerobic conditions until complete conversion of the thioester (3d, LC-MS control, LC-MS samples were prepared with 100 mM DTT in water).

Subsequently, TCEP x HCl was added (0.44 mg, 1.53 µmol, 12.5 equiv.) and the reaction mixture was purified after 30 min by RP-HPLC (column [I], 30-45 % MeCN/H_2O + 0.1 %

HCOOH 3 mL/min). The target fractions were neutralized with 1 M Na_2CO_3 (approximately 20 µL/mL of eluate) and lyophilized. The residue was taken up in taken up in 1.2 mL of 10 % MeCN/H_2O containing 100 mM DTT, and desalted (column [M], 10 % MeCN/H_2O, 0.5 mL/min). The target fraction was lyophilized.

Yield of **38**: 1.44 mg (72.2 nmol, 58.9 %). RP-HPLC-MS: column [D], 20-60 % MeCN/H_2O + 0.1 % HCOOH. HR-MS of **38**: *m/z* (exact mass) $C_{856}H_{1379}N_{209}O_{323}S_6$ (19946.6230) calculated: 1995.4680 $[M+10H]^{10+}$, 1814.1534 $[M+11H]^{11+}$, 1663.0579 $[M+12H]^{12+}$, found: 1995.4706 $[M+10H]^{10+}$, 1814.1560 $[M+11H]^{11+}$, 1663.0594 $[M+12H]^{12+}$.

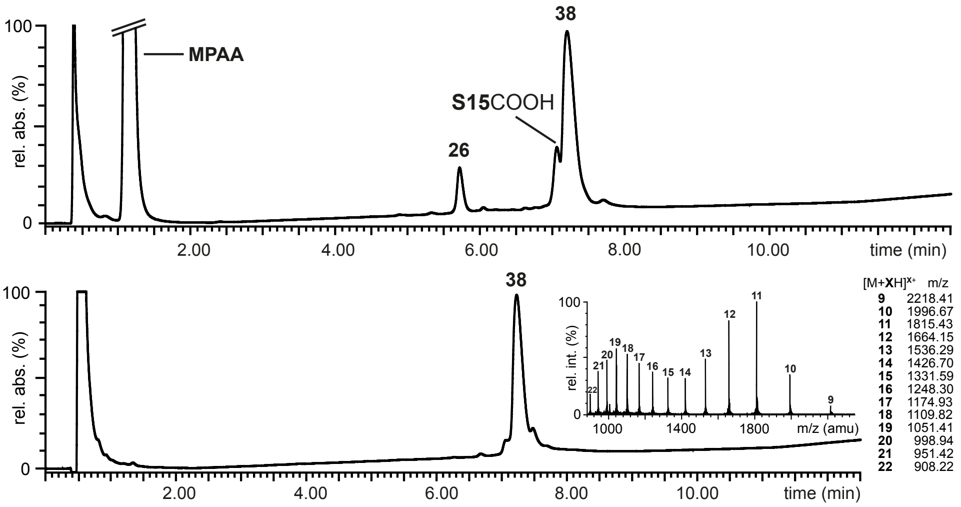

Figure S51: RP-HPLC-MS EPO 29-166 sialoglycopeptide **38** *via* NCL a) crude, b) after RP-HPLC.

6.9 Desulfurization of EPO 29-166 Sialoglycopeptide 38

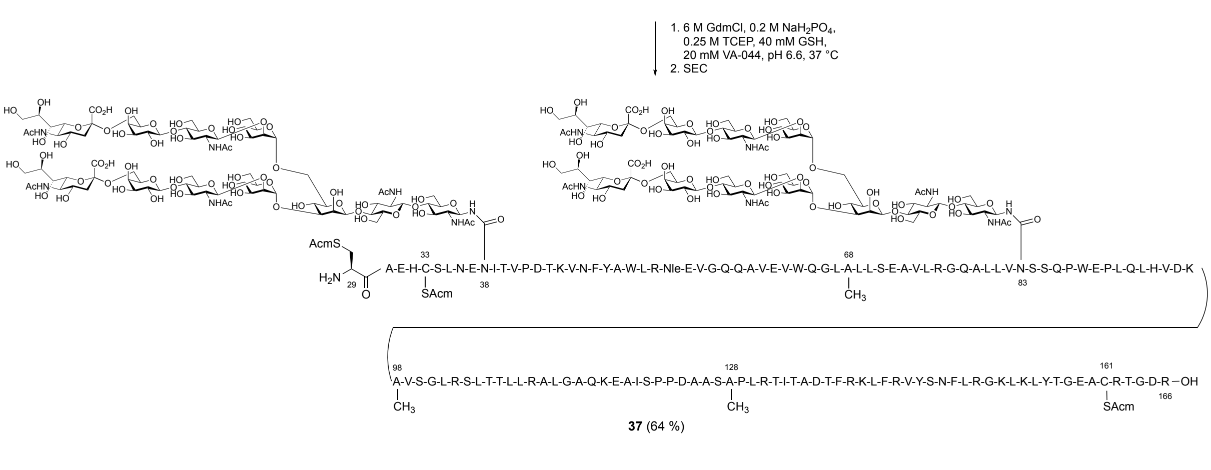

Figure S52: Desulfurization of EPO 29-166 glycopeptide **38**.

Under anaerobic conditions, a buffer (6 M GdmCl, 0.2 M NaH_2PO_4) was prepared and adjusted to pH = 7.0. A TCEP stock solution was prepared with this buffer (0.5 M TCEP x HCl) and the pH was adjusted to pH 7.0. Furthermore, a glutathione stock solution (160 mM glutathione, giving pH 4.7) and a VA-044 stock solution (80 mM VA-044, giving pH 6.9) were prepared with the GdmCl buffer. The glycopeptide **38** (2.01 mg, 100.7 nmol, 1 equiv.) was dissolved in the 100.7 µL of the TCEP stock solution (500 equiv.) followed by the glutathione and VA-044 stock solutions (50.4 µL each). The reaction mixture (final pH 6.6) was shaken under anaerobic conditions at 37 °C until completion (3 h according to HRMS, LC-MS samples were prepared with 150 mM DTT in water. Subsequently, the reaction mixture was diluted with 200 µL of 20 % MeCN/H_2O + 0.2 % HCOOH, desalted by gel filtration (column [M], 10 % MeCN/H_2O + 0.1 % TFA, 1.5 mL/min) and lyophilized.

Yield of **37**: 1.27 mg (63.9 nmol, 63.5 %). RP-HPLC: column [D], 20-60 % MeCN/H_2O + 0.1 % HCOOH. HR-MS of **37**: *m/z* (exact mass) $C_{856}H_{1379}N_{209}O_{323}S_3$ (19850.7068) calculated: 2206.6414 $[M+9H]^{9+}$, 1986.0780 $[M+10H]^{10+}$, 1805.6170 $[M+11H]^{11+}$, 1655.2328 $[M+12H]^{12+}$, found: 1986.0911 $[M+10H]^{10+}$, 1805.6192 $[M+11H]^{11+}$, 1655.2354 $[M+12H]^{12+}$.

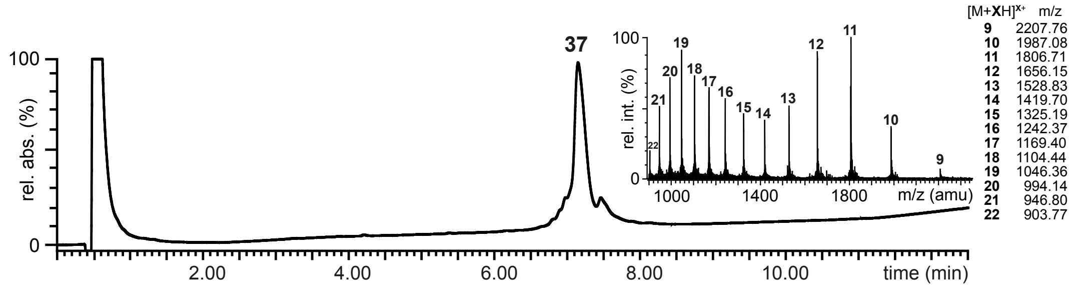

Figure S53: RP-HPLC-MS EPO 29-166 sialoglycopeptide **37** after SEC.

6.10 Cleavage of Acm groups from EPO 29-166 Sialoglycopeptide 37

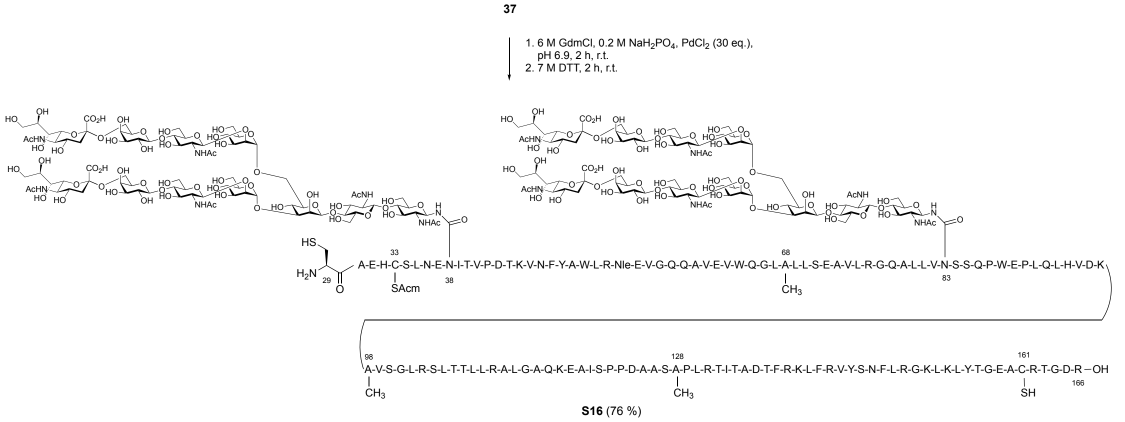

S16 (76 %)

Figure S54: Deprotection of EPO 29-166 glycopeptide **37**.

A buffer (6 M GdmCl, 0.2 M NaH_2PO_4) was prepared and adjusted to pH = 6.9. The buffer was degassed with argon for 30 min. Subsequently, anhydrous $PdCl_2$ (2.25 mg, 12.69 µmol) was suspended in 940 µL of this buffer and shaken for 1 h under argon for complete dissolution (13.5 mM Pd^{II}). 62.64 µL of the Pd^{II} stock solution (150.0 µg $PdCl_2$, 845.7 nmol, 30 equiv.) were added to the Acm-protected glycopeptide **37** (0.56 mg, 28.2 nmol, 1 equiv.). under argon. Under the exclusion of light the mixture was incubated for 2h. Subsequently, DTT (67.6 mg) was added to the reaction mixture after 2 h. The mixture was shaken for 2 h and purified by RP-HPLC (column [H], 10-60 % MeCN/H_2O + 0.1 % HCOOH, 1 mL/min). The target fractions were lyophilized. LC-MS samples were prepared with 100 mM DTT in water.

Yield of **S16**: 0.42 mg (21.38 nmol, 75.8 %). RP-HPLC-MS: column [D], 20-60 % MeCN/H_2O + 0.1 % HCOOH. HR-MS of **S16**: *m/z* (exact mass) $C_{847}H_{1364}N_{206}O_{320}S_3$ (19637.5955) calculated: 1964.7668 $[M+10H]^{10+}$, 1786.2432 $[M+11H]^{11+}$, 1637.4736 $[M+12H]^{12+}$, found: 1964.7778 $[M+10H]^{10+}$, 1786.2514 $[M+11H]^{11+}$, 1637.4817 $[M+12H]^{12+}$.

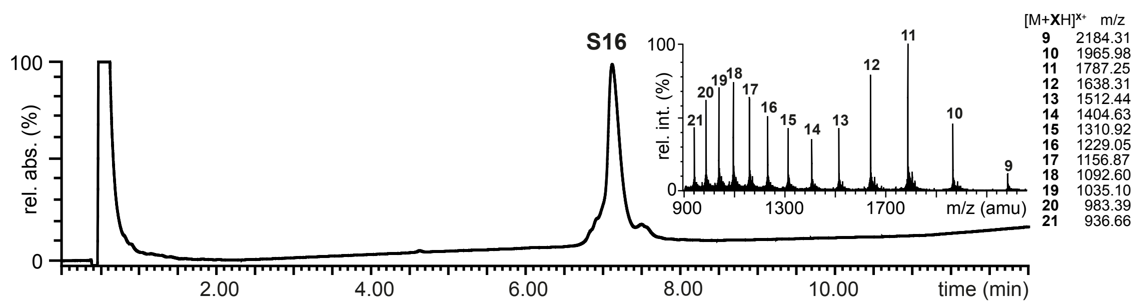

Figure S55: RP-HPLC-MS of EPO 29-166 sialoglycopeptide **S16** after RP-HPLC.

6.11 Synthesis and Refolding of EPO S

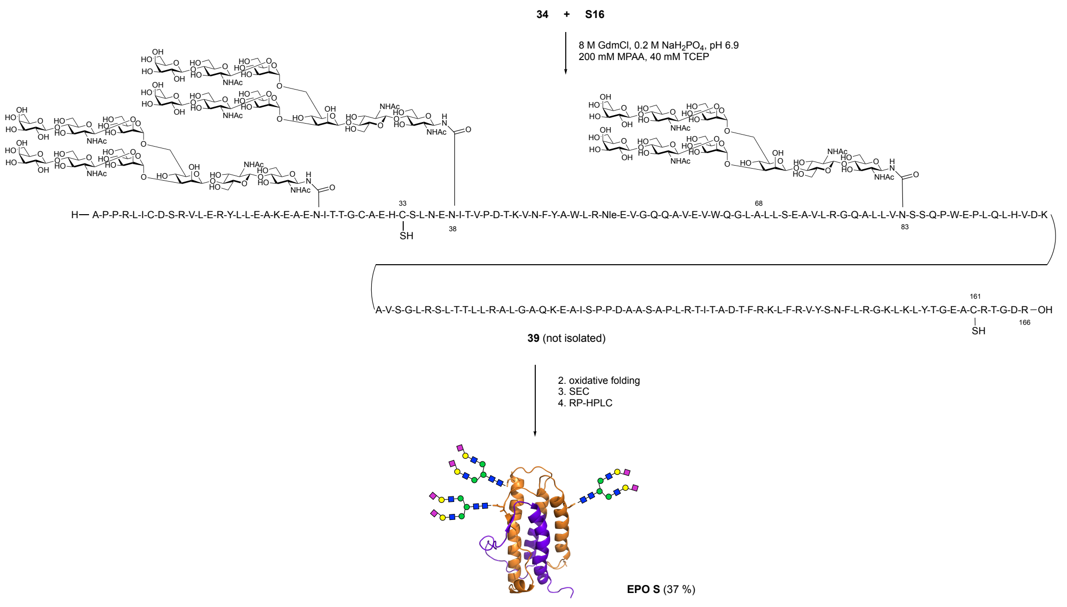

Figure S56: Synthesis of EPO **S**.

The ligation buffer (8 M GdmCl, 0.2 M NaH_2PO_4, 40 mM TCEP, 0.1 M MPAA) was mixed under anaerobic conditions and adjusted to pH = 6.9. The glycopeptide thioester **34** (0.53 mg, 95.42 nmol, 2.5 equiv.) was dissolved in 19.1 µL of ligation buffer and added to glycopeptide **S16** (0.75 mg, 38.17 nmol, 1 equiv.). The mixture was incubated under anaerobic conditions until complete conversion of the thioester (6 d, LC-MS control). The ligation mixture was diluted with 9.54 mL of the dilution buffer (Table S5) and transferred to a tubular dialysis membrane (ZelluTrans/Roth V series, cutoff = 5 kDa, 40 mm flat width, thickness 20-30 µm, regenerated cellulose), and then dialyzed sequentially against the buffers shown in Table S5 (390 mL each) with constant agitation. The dialysis conditions are listed in Table S6.

After completion of the dialysis the protein solution was concentrated to a volume of 1.6 mL (Amicon 8010 stirred cell with membrane UP010 P from MICRODYN-NADIR, cutoff = 10 kDa). Monomeric **EPO S** was isolated by size exclusion chromatography (column [P], 0.15 M NaCl, 50 mM NaH_2PO_4, 0.5 M L-arginine, pH 7.5, 1 mL/min). The eluate was concentrated by ultrafiltration to a volume of 1.6 mL, purified by RP-HPLC (column [H], 30-50 % MeCN/H_2O + 0.1 % TFA, 1 mL/min) and lyophilized.

Yield of **EPO S**: 0.35 mg (14.0 nmol, 36.7 %). RP-HPLC-MS: column [D], 20-60 % MeCN/H_2O + 0.1 % HCOOH. HR-MS of **EPO S**: *m/z* (exact mass) $C_{1068}H_{1723}N_{251}O_{424}S_4$ (24976.9862) calculated: 1785.0777 $[M+14H]^{14+}$, 1666.1397 $[M+15H]^{15+}$, 1562.0689 $[M+16H]^{16+}$, found: 1785.0852 $[M+14H]^{14+}$, 1666.1468 $[M+15H]^{15+}$, 1562.0761 $[M+16H]^{16+}$.

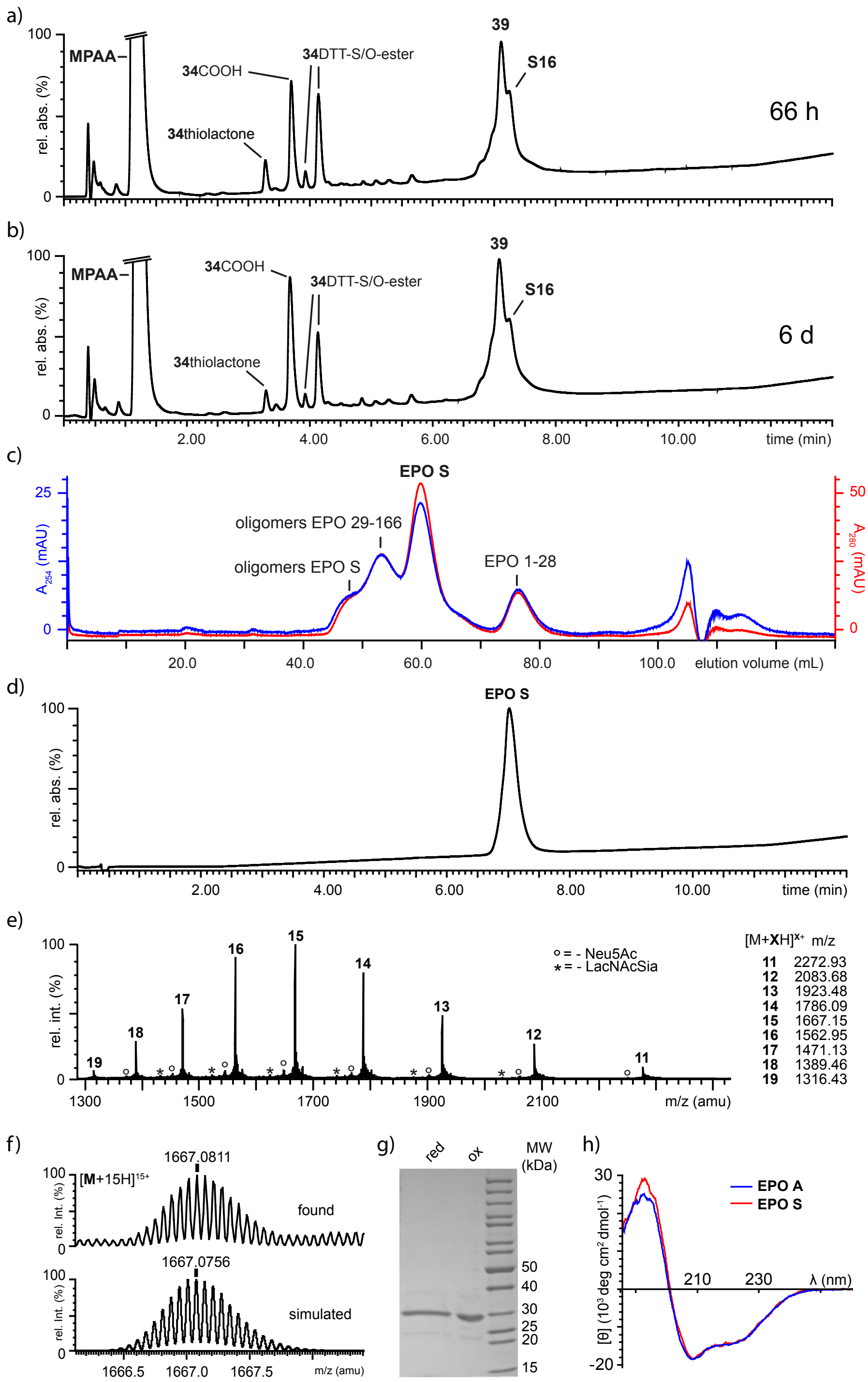

Figure S57: Synthesis and purification of **EPO S**: a) RP-HPLC-MS of crude NCL mixture after 66 h, b) RP-HPLC-MS of crude NCL mixture after 6 d, c) Separation mono- and oligomers by gelfiltration (Superdex 75), d) RP-HPLC-MS of **EPO S**, e) HRMS of **EPO S,** f)) found and simulated HR-isotope patterns of $[M+15H]^{15+}$ charge state, g) SDS-PAGE of **EPO S** reduced and oxidized, and h) superposition of CD-spectra of **EPO A** and **S**.

The CD spectrum was recorded with 250 µL of **EPO S** (0.16 mg **EPO S**/mL) in ultrapure water at 23 °C.

7. Enzymatic Sialylation of Glycopeptides

7.1 Sialylation of EPO 29-97 Glycopeptide Hydrazide 25

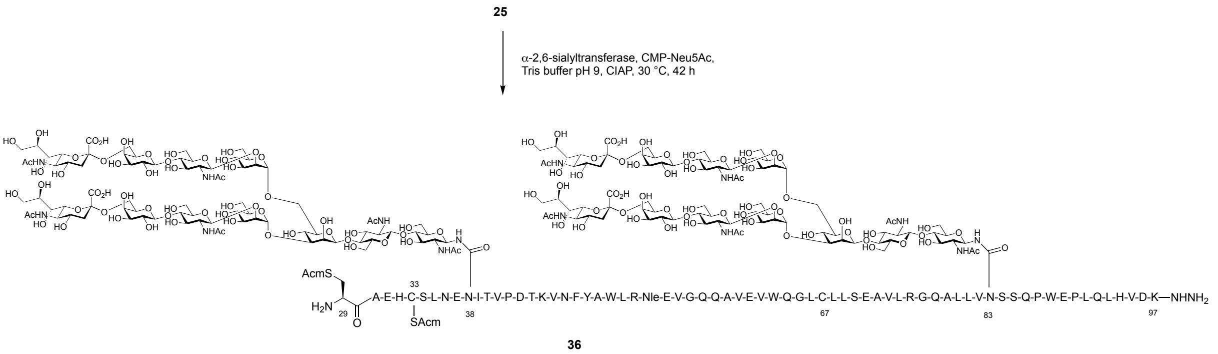

Figure S58: Sialylation of EPO 29-97 glycopeptide hydrazide **25**.

The glycopeptide hydrazide **25** (0.25 mg, 22.3 nmol, 1 equiv.) was dissolved in 1.68 µL of water. C'IAP (0.40 µL, 8.92 U) and 1.7 µL of a stock solution containing CMP-Neu5Ac (133.6 µg, 178.5 nmol, 8 equiv.) in 250 mM Tris buffer, pH 9. Subsequently, 1 µL of α-2,6-sialyltransferase from *P. damselae* was added (2.9 µg). The solution was incubated at 30 °C (*c* **25** = 5 mM). After 12 h, an aliquot of 0.5 µL was diluted with 4.5 µL of 250 mM DTT in 100 mM Tris, pH 9 and analyzed by LC-MS. Subsequently, 8 equiv. of CMP-Neu5Ac were added. After incubation for 5 and 13 h further CMP-Neu5Ac (16 equiv. each) was added.
RP-HPLC-MS: column [B], 20-60 % MeCN/H_2O + 0.1 % HCOOH.

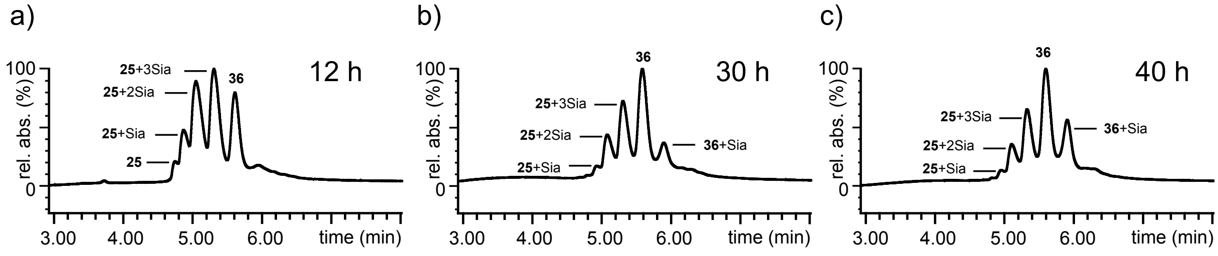

Figure S59: Enzymatic sialylation of EPO 29-97 hydrazide **25** : RP-HPLC-MS a) after 12 h, b) after 30 h and c) after 40 h.

7.2 Enzymatic α-2,6-Sialylation of EPO 29-166 Glycopeptide 28

28

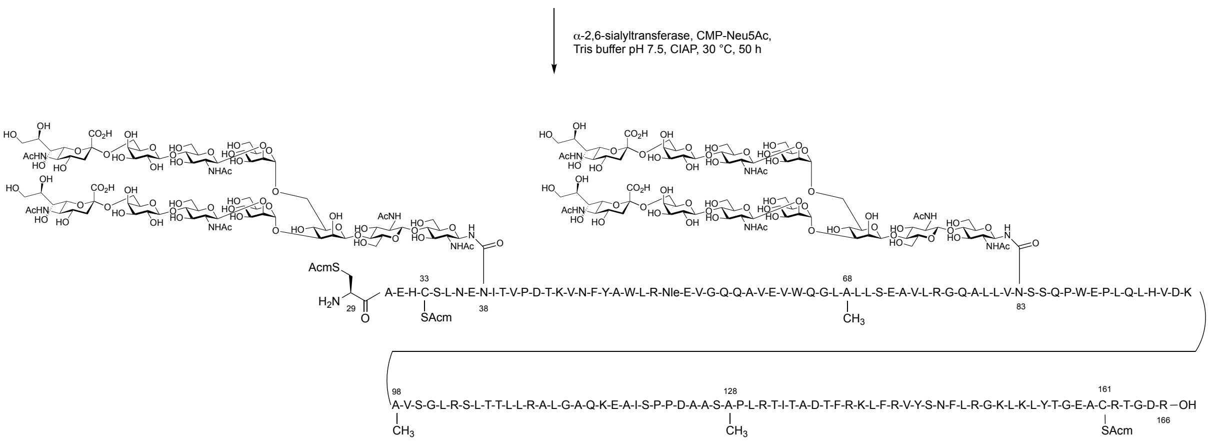

37

Figure S60: Sialylation of EPO 28-166 glycopeptide **28**.

The glycopeptide **28** (50 µg, 2.67 nmol, 1 equiv.) was dissolved in 4.12 µL of water containing CMP-Neu5Ac (16.0 µg, 21.39 nmol, 8 equiv.) Subsequently, 0.51 µL of 250 mM Tris buffer pH 7.5 were added, followed by addition of 0.48 µL of CIAP (2 U/µL) and 0.24 µL of α-2,6-sialyltransferase from *P. damselae* was added (0.7 µg). The solution was incubated at 30 °C (*c* **25** = 0.5 mM). After 1.5 h, an aliquot of 0.21 µL was diluted with 19.79 µL H_2O and analyzed by HRMS. Subsequently, and after 1.5 h further CMP-Neu5Ac (8 equiv. each) were added. After a total reaction time of 31 h further CMP-Neu5Ac (16 equiv.) was added.

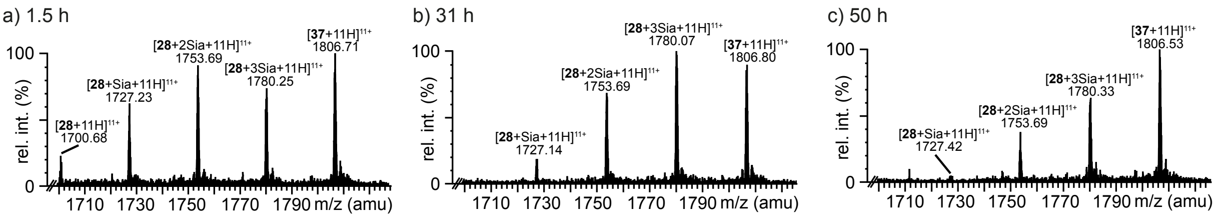

Figure S61: Enzymatic Sialylation of EPO 29-166 28: RP-HPLC-MS of reaction mixture a) after 1.5 h (8 equiv. of CMPNeu5Ac), b) after 31 h (24 equiv. of CMPNeu5Ac) and c) after 50 h (40 equiv. of CMPNeu5Ac).

7.3 Preparative Sialylation of EPO A

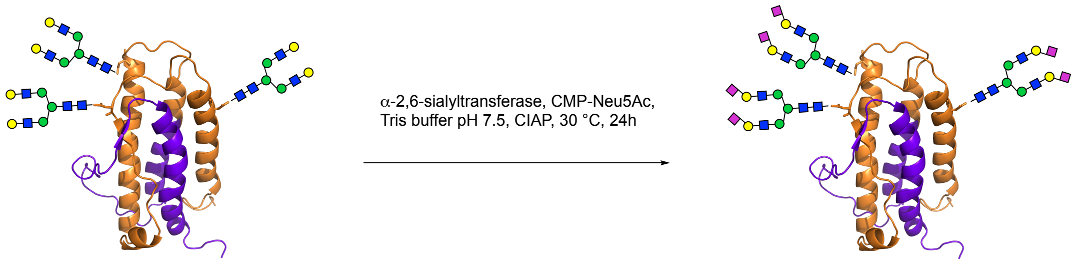

EPO A **EPO S** (49 %)

Figure S62: Sialylation of folded **EPO A**.

Purified **EPO A** (230 µg, 9.89 nmol, 1 equiv.) was dissolved in 13.7 µL of 25 mM Tris buffer, pH 7.5 and mixed with 1.79 µL of CIAP (2 U/µL). Subsequently, 1.33 µL of α-2,6-sialyltransferase from *P. damselae* (3.86 µg) and 2.96 µL of an aqueous stock solution containing CMP-Neu5Ac (88.9 µg, 118.7 nmol, 12 equiv.) were added. The reaction mixture was incubated at 30 °C. After 1.5 h, 3h and 4.5h three further additions of 0.15 µL of a stock solution containing 88.9 µg of CMP-Neu5Ac, 118.7 nmol, 12 equiv. in 25 mM Tris buffer, pH 7.5 were made. The reaction mixture was incubated for another 17.5 h at 30 °C, diluted to a volume of 200 µL with H_2O and purified by RP-HPLC (column [E], 5-95 % MeCN/H_2O + 0.1 % HCOOH, 0.25 mL/min). The eluate was immediately frozen and lyophilized.

Yield of **EPO S**: 0.12 mg (4.80 nmol, 48.5 %). RP-HPLC-MS: column [D], 20-60 % MeCN/H_2O + 0.1 % HCOOH.

HR-MS of **EPO S**: *m/z* (exact mass) $C_{1068}H_{1723}N_{251}O_{424}S_4$ (24976.9862) calculated: 1785.0777 $[M+14H]^{14+}$, 1666.1397 $[M+15H]^{15+}$, 1562.0689 $[M+16H]^{16+}$, found: 1785.0863 $[M+14H]^{14+}$, 1666.1463 $[M+15H]^{15+}$, 1562.0728 $[M+16H]^{16+}$.

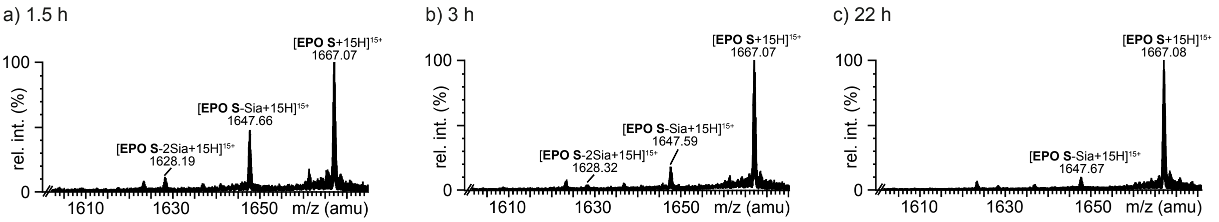

Figure S63: Enzymatic sialylation of **EPO A**: RP-HPLC-HRMS of crude reaction mixture a) after 1.5 h (12 equiv. CMPNeu5Ac), b) after 3 h (24 equiv. of CMPNeu5Ac) and c) after 22 h (48 equiv. of CMPNeu5Ac).

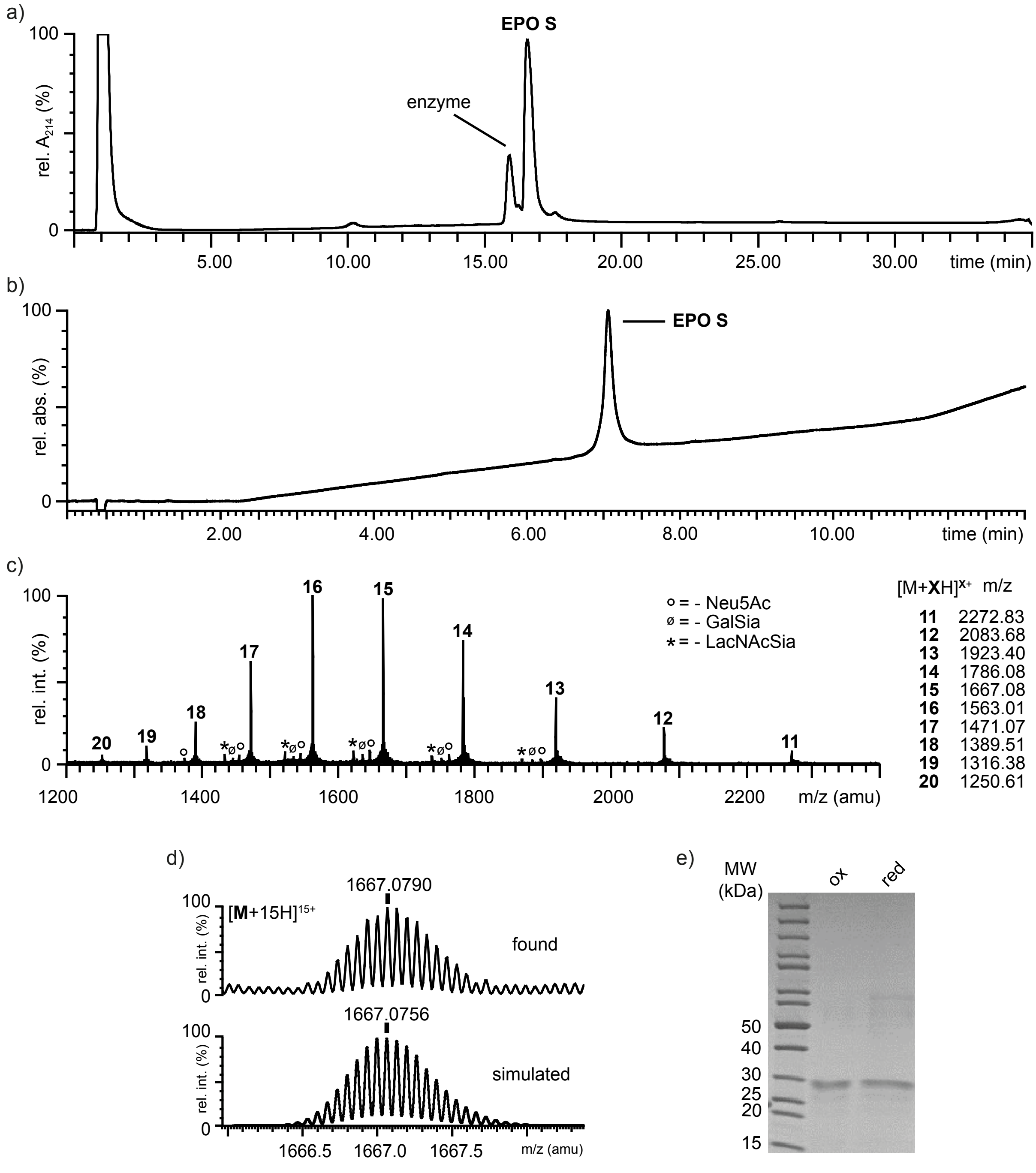

Figure S64: Sialylated **EPO S**: a) RP-HPLC-MS of crude enzymatic sialylation mixture after 22 h, b) RP-HPLC-MS of purified **EPO S**, c) HRMS of **EPO S,** d) found and simulated HR-isotope patterns of $[M+15H]^{15+}$ charge state, e) SDS-PAGE.

8. Expression of EPOR and Complexations with EPO Glycoforms

8.1 Overexpression and Purification of EPOR

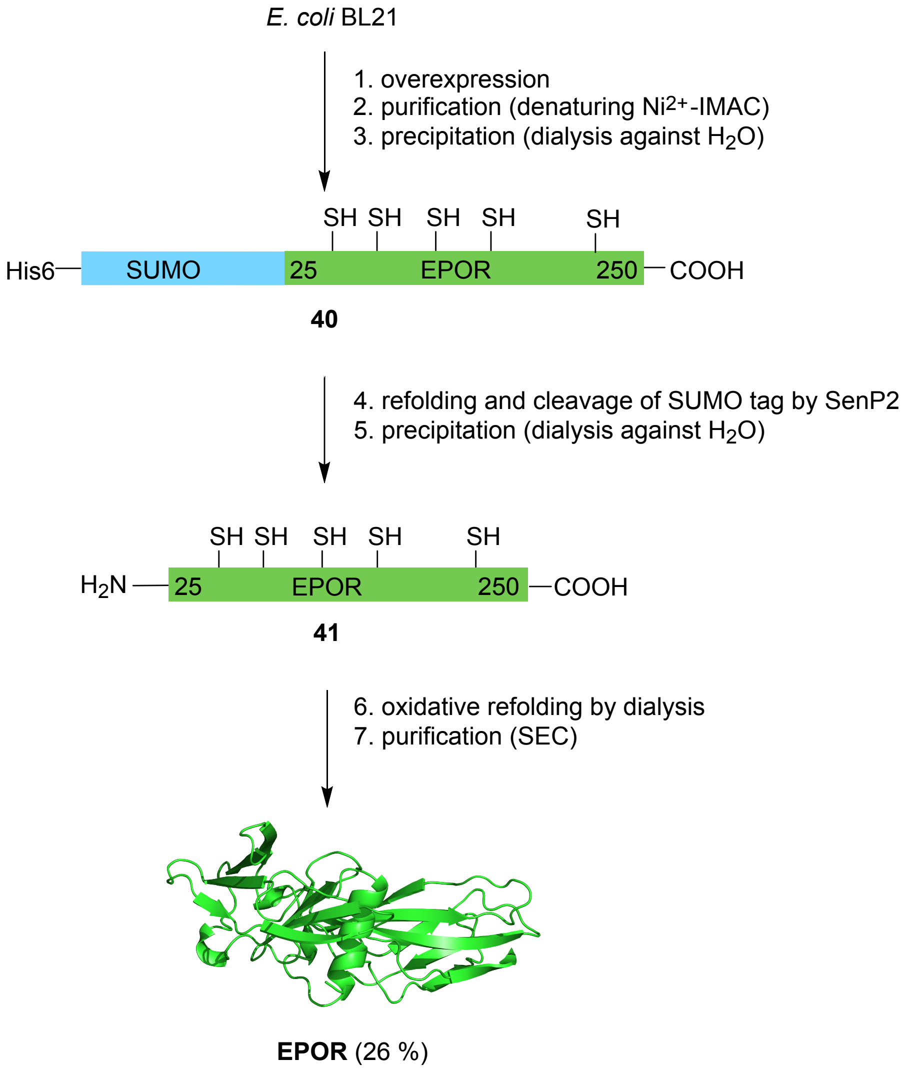

Figure S65: Recombinant expression and refolding of **EPOR**.

Amino acid sequence of His$_6$-SUMO-EPOR(25-250) **40**:

0 MGSSHHHHHH GSGLVPRGSA SMSDSEVNQE AKPEVKPEVK PETHINLKVS

50 DGSSEIFFKI KKTTPLRRLM EAFAKRQGKE MDSLRFLYDG IRIQADQTPE

100 DLDMEDNDII EAHREQIGGA PPPNLPDPKF ESKAALLAAR GPEELLCFTE

150 RLEDLVCFWE EAASAGVGPG NYSFSYQLED EPWKLCRLHQ APTARGAVRF

200 WCSLPTADTS SFVPLELRVT AASGAPRYHR VIHINEVVLL DAPVGLVARL

250 ADESGHVVLR WLPPPETPMT SHIRYEVDVS AGNGAGSVQR VEILEGRTEC

300 VLSNLRGRTR YTFAVRARMA EPSFGGFWSA WSEPVSLLTP SDLDP

LB-medium: 10 g/L tryptone, 5 g/L yeast extract, 10 g/L NaCl, pH 7
lysis buffer: 0.5 M NaCl, pH 8.5
binding buffer: 6 M GdmCl, 20 mM NaH_2PO_4, 20 mM imidazole, 0.5 M NaCl, pH 7.4
elution buffer: 6 M GdmCl, 20 mM NaH_2PO_4, 250 mM imidazole, 0.5 M NaCl, pH 7.4

The EPO receptor was expressed as a His_6-SUMO-EPOR fusion protein (**40**). The vector pET11a_His_6-SUMO-EBP25-250 was obtained from Genscript. *E. coli* BL21 (DE3) containing this vector were grown in 5 L flasks containing 1.25 L of LB-medium at 37 °C. The overexpression of **40** was induced after reaching an OD_{600} of 0.6 by addition of 1.25 mL of 1 M IPTG (final concentration 1 mM). The cells were harvested by centrifugation (10000 g, 4 °C, 10 min) after 4 h of incubation. Yield: 4.67 g of cell pellet from 1.25 L of LB-medium.

The cells were suspended in 93 mL of lysis buffer and lysed with a Sonopuls ultrasonic homogenizer HD 2070 (15x 20 s, 50 % cycle, 60 % power). The crude lysate was pelleted by centrifugation (5000 g, 4 °C, 10 min). The pellet was resuspended in 93 mL of lysis buffer and pelleted by centrifugation (2 cycles). The pelleted inclusion bodies were suspended in 47 mL of binding buffer (10 mL/g). The suspension was centrifuged (4 °C, 40000 g, 30 min) and the supernatant filtered through a 0.45 µm PVDF filter (Roth, Germany). The filtrate was loaded onto a Protino Ni^{2+}-NTA-Agarose column (20 mL, GE Healthcare, Sweden) with a peristaltic pump (1 mL/min, P-1, GE Healthcare, Sweden). The column was washed with binding buffer (3 mL/min) until the absorption at 280 nm was constant. His_6-SUMO-EPOR **40** was eluted with elution buffer (3 mL/min), precipitated by dialysis (against 1 L of H_2O, 3x) and collected by centrifugation (25000 g, 4 °C, 3 min). Yield: 56.43 mg of **40** from 1.25 L of LB-medium.
RP-HPLC-MS of **40**: column [D], 20-60 % MeCN/H_2O + 0.1 % HCOOH.
a)

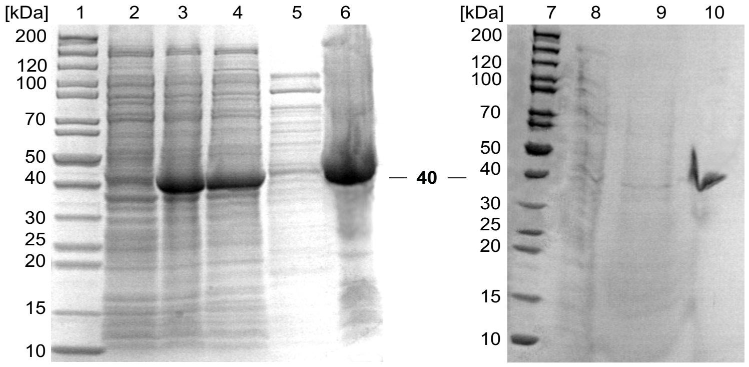

b)

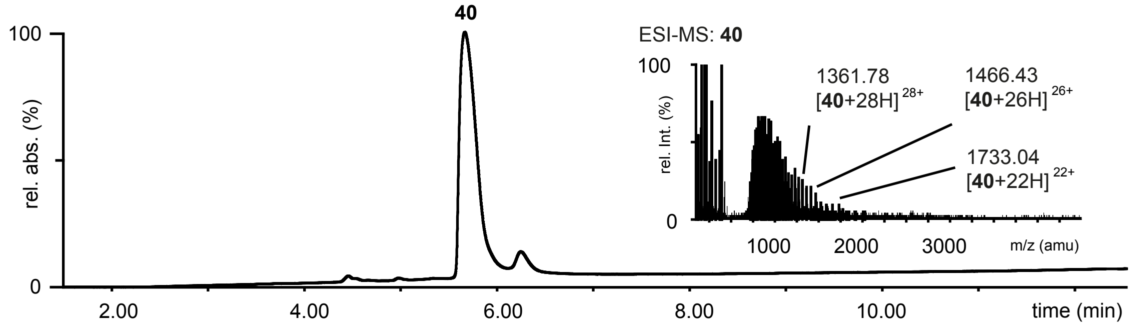

Figure S66: a) SDS-PAGE of the overexpression and purification of **40**. Lane 1: molecular weight standard; lane 2: cells before addition of IPTG; lane 3: cells 2 h post addition of IPTG; lane 4: cells 4 h post addition of IPTG; lane 5: soluble fraction after cell lysis; lane 6: insoluble fraction after cell lysis; lane 7: molecular weight standard; lane 8: breakthrough (Ni^{2+}-IMAC); lane 9: washing step (Ni^{2+}-IMAC); lane 10: eluate of **40** (Ni^{2+}-IMAC), b) RP-HPLC-MS of **40**.

8.2 Refolding and Purification of EPOR

dissolving buffer I: 0.1 M Tris, 3.5 M urea, 10 mM Lys, 10 mM DTT, pH 8.5
dissolving buffer II: 0.1 M Tris, 6 M urea, 10 mM Lys, 10 mM DTT, pH 8.5
refolding buffer I: 50 mM Tris, 10 mM L-lysine, pH 8.5
refolding buffer II: 0.1 M Tris, 4.5 M urea, 10 mM Lys, 2 mM MPAA, 0.4 mM GSH, pH 8.5
refolding buffer III: 0.1 M Tris, 3 M urea, 10 mM Lys, 2 mM MPAA, 0.4 mM GSH, pH 8.5
refolding buffer IV: 0.1 M Tris, 1.5 M urea, 10 mM Lys, 2 mM MPAA, 0.4 mM GSH, pH 8.5
refolding buffer V: 0.1 M Tris, 0.5 M urea, 10 mM Lys, 2 mM MPAA, 0.4 mM GSH, pH 8.5
refolding buffer VI: 50 mM Tris, 200 mM NaCl, pH 7.5

6 mg of His_6-SUMO-EPOR(25-250) **40** (1 eq, 0.16 µmol) and 1.9 mg of L-cysteine (100 eq, 15.8 µmol) were dissolved (600 rpm, 1 h) in 0.3 mL of dissolving buffer I. After addition of 2.7 mL of refolding buffer I, the cleavage of the His_6-SUMO tag was induced by addition of 7.3 µL of SUMO protease SenP2 (11.08 mg/mL). After complete cleavage (1 d) the liberated peptide EPOR(25-250) was precipitated by dialysis (1 L of H_2O, 3 x), collected by centrifugation (25000 g, 4 °C, 3 min), washed with H_2O (1 mL, 3 x) and lyophilized. Yield of crude EPOR(25-250): 5.79 mg (0.23 µmol, 89 %).
3 mg of crude EPOR(25-250) were dissolved in 30 mL of dissolving buffer II in an anaerobic chamber. After 6 h the solution was removed from the anaerobic chamber and dialysed against refolding buffers II - VI (750 mL, 12 h each). The refolding solution was concentrated (Amicon

stirred cell, 10 kDa cut off, 50 mL) and purified by gel filtration (HiLoad 16/600 Superdex 75 pg, 60.5 x 1.6 cm, GE Healthcare, 50 mM Tris/200 mM NaCl, pH 7.5, 1 mL/min). The monomeric fraction (69 min) was concentrated (Amicon centrifugal filters ultracel, 10 kDa cut off, 0.5 mL, 14000 g). The yield was determined by absorption at 280 nm and the purity was controlled by LC-MS and HR-MS.

RP-HPLC-MS of **EPOR**: column [D], 20-60 % MeCN/H_2O + 0.1 % HCOOH.

HR-MS of **EPOR**: direct injection in water. 150 µg of **EPOR** in 100 µL 50 mM Tris/ 200 mM NaCl, pH 7.5 were loaded onto a PD Mini Trap G25 column (GE healthcare) followed by 400 µL of water. Elution with 1 mL of water gave **EPOR** in a concentration of 0.1 mg/mL (A^{280}).

Yield of **EPOR**: 0.77 mg (0.031 µmol, 26 %) ESI-MS: m/z (average isotopes): $C_{1113}H_{1715}N_{307}O_{325}S_7$, (24821.21); calculated: $(M+24H)^{24+}$ 1035.22, $(M+23H)^{23+}$ 1080.18, $(M+22H)^{22+}$ 1129.24; found: 1035.31, 1080.29, 1129.34. ESI-HRMS: m/z (exact mass): $C_{1113}H_{1715}N_{307}O_{325}S_7$, (24805.52); calculated: $(M+24H)^{24+}$ 1035.1554, $(M+23H)^{23+}$ 1080.1184, $(M+22H)^{22+}$ 1129.1689; found: 1035.1911, 1080.1123, 1129.1627.

The CD spectrum was recorded with 200 µL of **EPOR** (0.13 mg **EPOR**/mL) in 50 mM Tris, 200 mM NaCl, pH 7.5 at 20 °C.

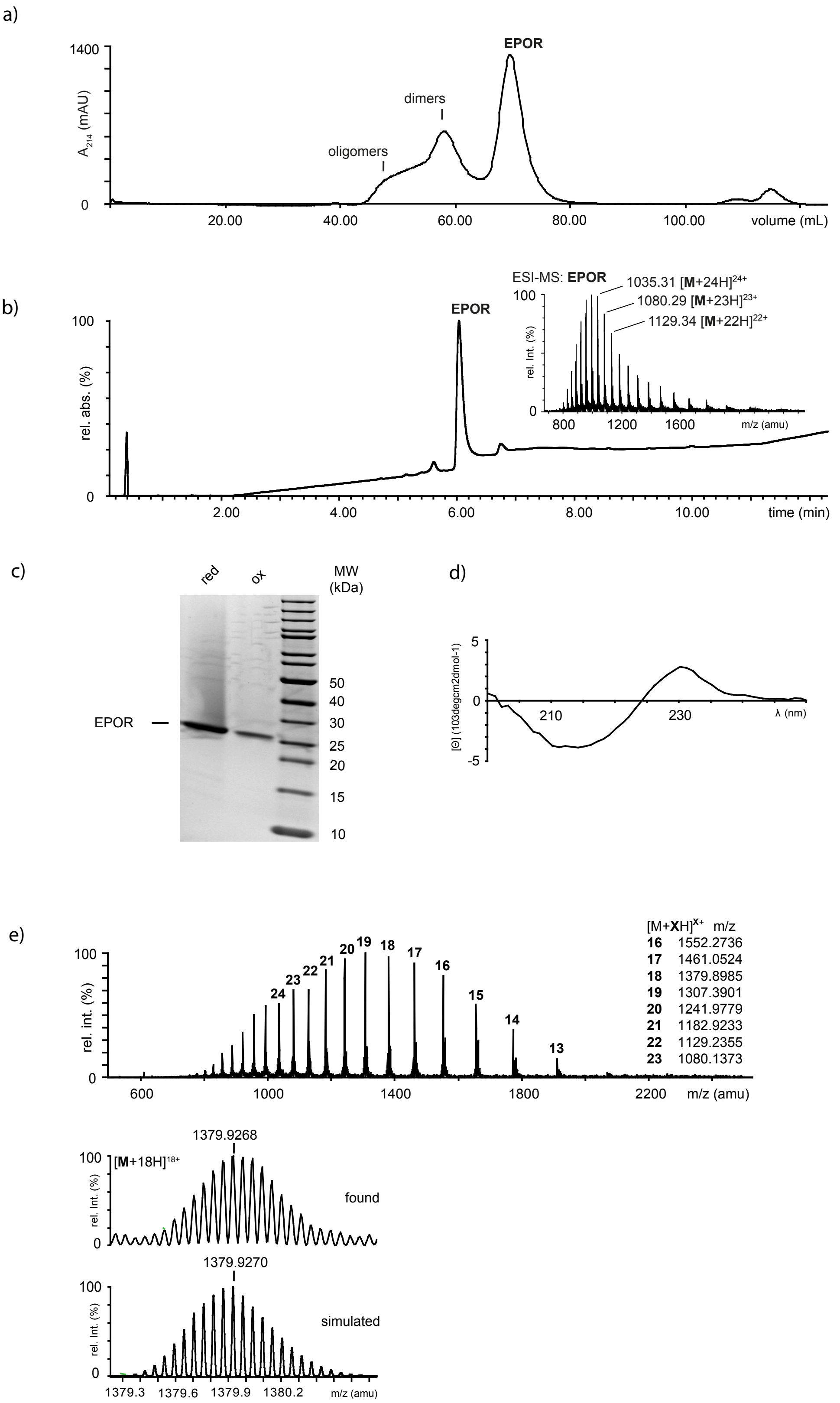

Figure S67: Recombinant expression of **EPOR**: a) SEC of refolding mixture b) RP-HPLC-MS of purified **EPOR** b) RP-HPLC-MS of purified **EPO S**, c) SDS-PAGE of EPOR reduced (red) or without prior reduction ox) d) CD-spectrum of **EPOR**. e) HRMS of **EPO S,** found and simulated HR-isotope patterns of $[M+18H]^{18+}$ charge state.

8.3 Complexation of EPO glycoforms with EPOR

The biological recognition of the synthetic EPO glycoforms and commercial reference EPO from CHO cells (PeproTech, Germany) was tested by complexation with the expressed EPO receptor **EPOR** and analyzed by size exclusion chromatography (Superdex 75 PC, 3.2 mm x 300 mm, 13 µm, GE Healthcare, 75 µL/min, PBS buffer pH 7.4, Agilent 1100 HPLC).

MW (calcd.): **EPOR** 24825.17 Da, **EPO A** 23244.57, **EPO S** 24992.11, **EPO$_{CHO}$** 37000.

3.7 µL of **EPOR** (8.5 µg, 0.34 nmol, 2.3 mg **EPOR**/mL in 50 mM Tris/200 mM NaCl, pH 7.5) were incubated for 30 minutes at room temperature with either 8 µL of **EPO A** (8 µg, 0.34 nmol, 1 mg **EPO A**/mL in water) or 9.5 µL of **EPO S** (8.6 µg, 0.34 nmol, 0.9 mg **EPO S**/mL in water). Furthermore 1.4 µL of **EPOR** (3.2 µg, 0.13 nmol, 2.3 mg **EPOR**/mL in 50 mM Tris/200 mM NaCl, pH 7.5) were incubated for 30 minutes at room temperature with 4.8 µL of **EPO$_{CHO}$** (4.8 µg, 0.13 nmol, 1 mg **EPO$_{CHO}$**/mL in water). The total volume of each complexation was adjusted to 12 µL with PBS pH 7.4 and 10 µL of the mixtures were analyzed by SEC. Fractions containing the respective **EPO**/**EPOR** complexes (~100 µL total) were collected and analysed by SDS-PAGE (10-20 % Tris/Tricine-gradient gel). For LC-MS analysis 50 µL of each **EPO**/**EPOR** complex fraction were incubated with 10 µL of MeCN containing 3 % TFA for 1 h prior to analysis (column [D], 20-60 % MeCN/H_2O + 0.1 % HCOOH).

References:

[1] A. Reif, S. Siebenhaar, A. Tröster, M. Schmälzlein, C. Lechner, P. Velisetty, K. Gottwald, C. Pöhner, I. Boos, V. Schubert, S. Rose-John, C. Unverzagt, *Angew. Chem. Int. Ed.* **2014**, 53, 12125 –12131.

[2] M. Kiyozumi, K. Kato, T. Komori, A. Yamamoto, T. Kawasaki, H. Tsukamoto, *Carbohydr. Res.* **1970**, 14, 355 – 364.

[3] M. Sun, Y. Li, H. A. Chokhawala, R. Henning, X. Chen, *Biotechnol. Lett.* **2008**, 30, 671–676.

[4] S. Eissler, M. Kley, D. Bächle, G. Loidl, T. Meier, D. Samson, *J. Pept. Sci.* **2017**, 23, 757–762.

[5] W. C. Chan, P. D. White, *Eds.*, *Fmoc Solid Phase Peptide Synthesis: A Practical Approach*, Oxford University Press, New York, **2000**.

[6] C.-D. Chang, J. Meienhofer, *Int. J. Pept. Protein Res.* **2009**, 11, 246–249.

[7] E. Atherton, R. C. Sheppard, P. Ward, *J. Chem. Soc. Perkin 1* **1985**, 2065.

[8] C. Heinlein, D. Varon Silva, A. Tröster, J. Schmidt, A. Gross, C. Unverzagt, *Angew. Chem. Int. Ed.* **2011**, 50, 6406 (see supplement pages 17-18).

[9] V. Ullmann, M. Rädisch, I. Boos, J. Freund, C. Pöhner, S. Schwarzinger, C. Unverzagt, *Angew. Chem. Int. Ed.* **2012**, 51, 11566-11570.

[10] M. Hessefort, H. Hessefort, S. Seeleithner, A. Gross, M. Lott, D. Rau, L. Kern, C. Unverzagt, *J. Pept. Sci.* **2021**, 27, e3283.
